# Supplementary material for: Template switching during DNA replication is a prevalent source of adaptive gene amplification
Source: eLife. 2025 Feb 3;13:RP98934. doi: 10.7554/eLife.98934 (PMC11790251; doi:10.7554/eLife.98934)

Wildtype population 1

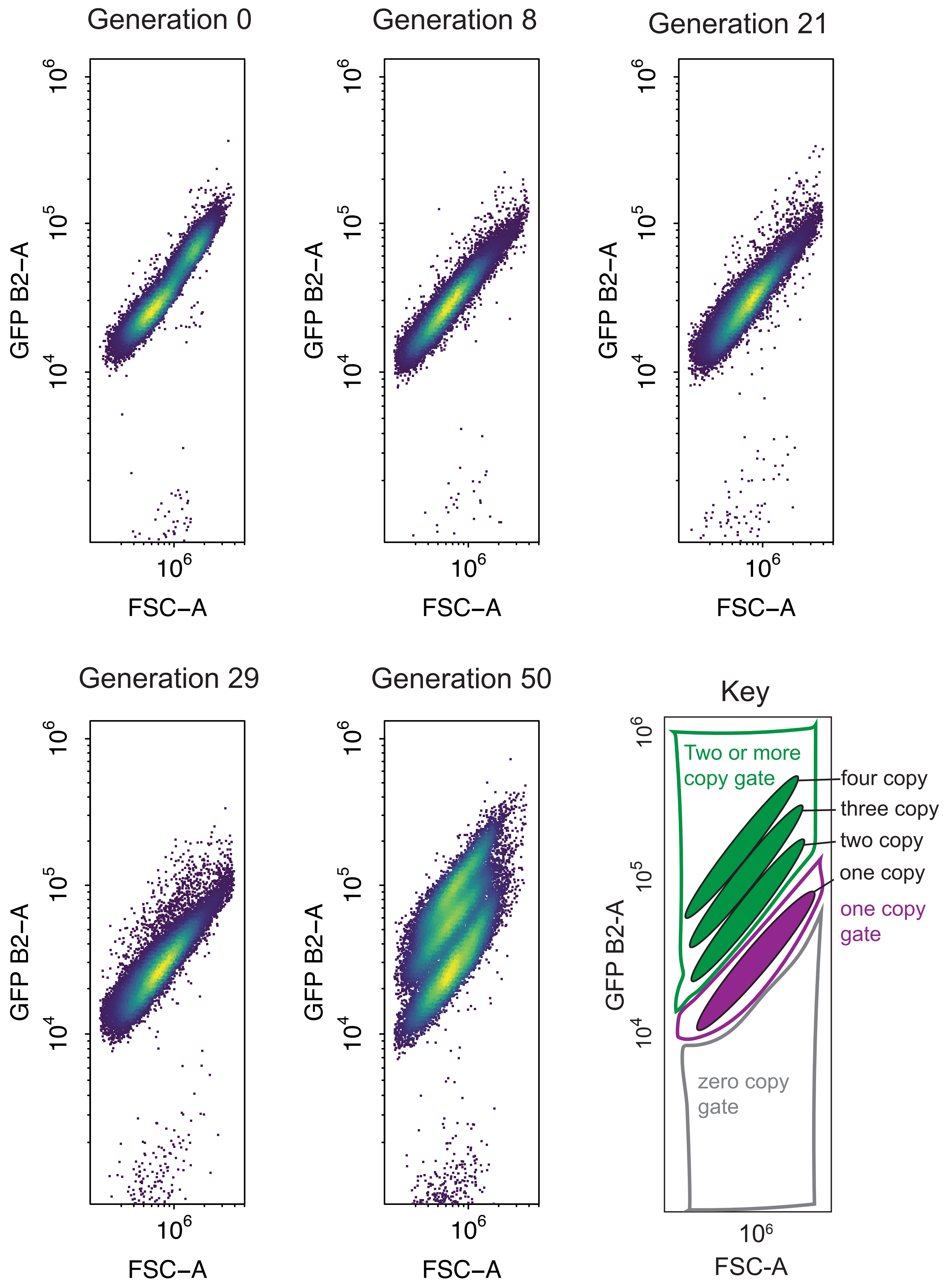

Wildtype population 1

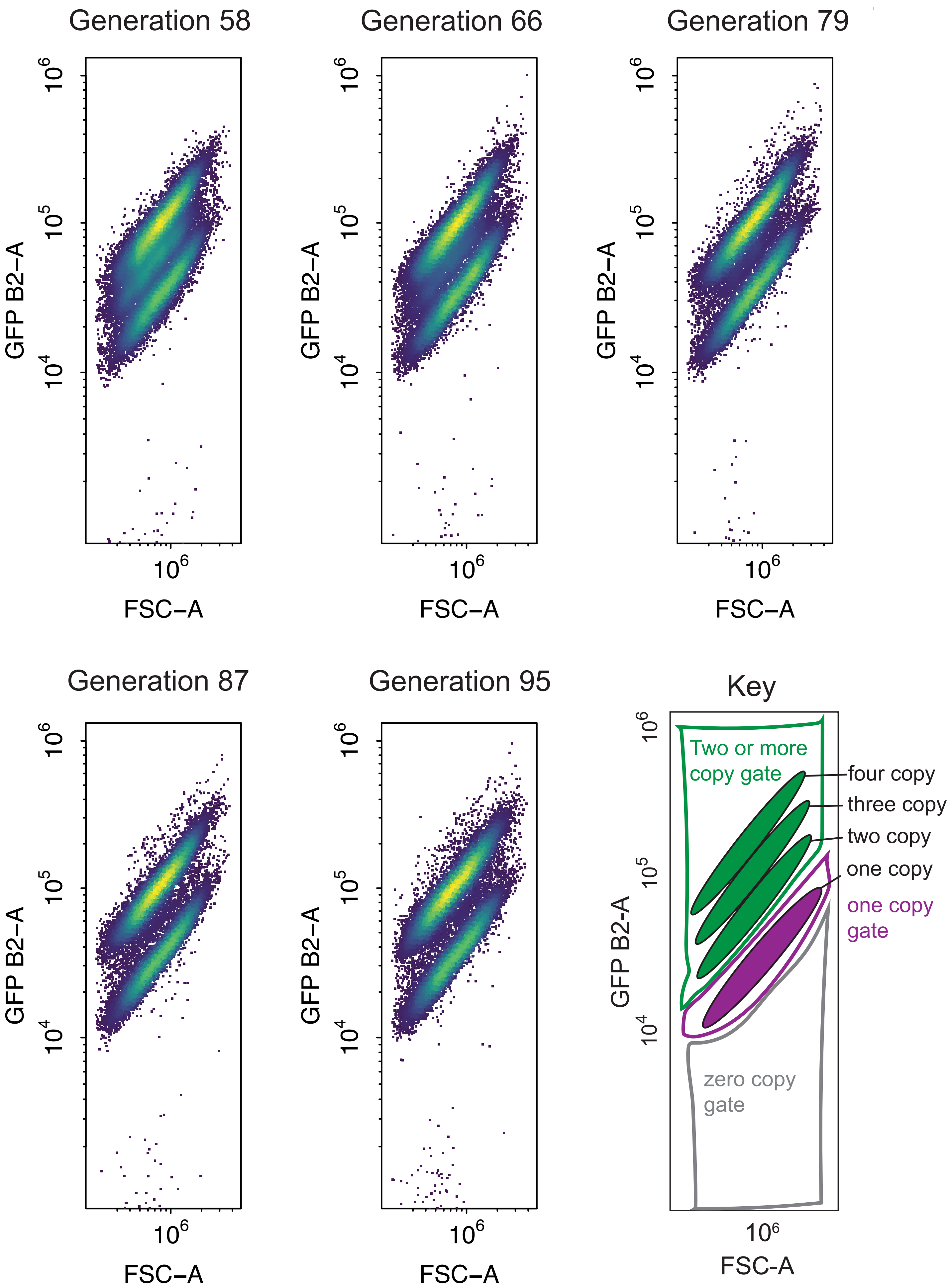

Wildtype population 1

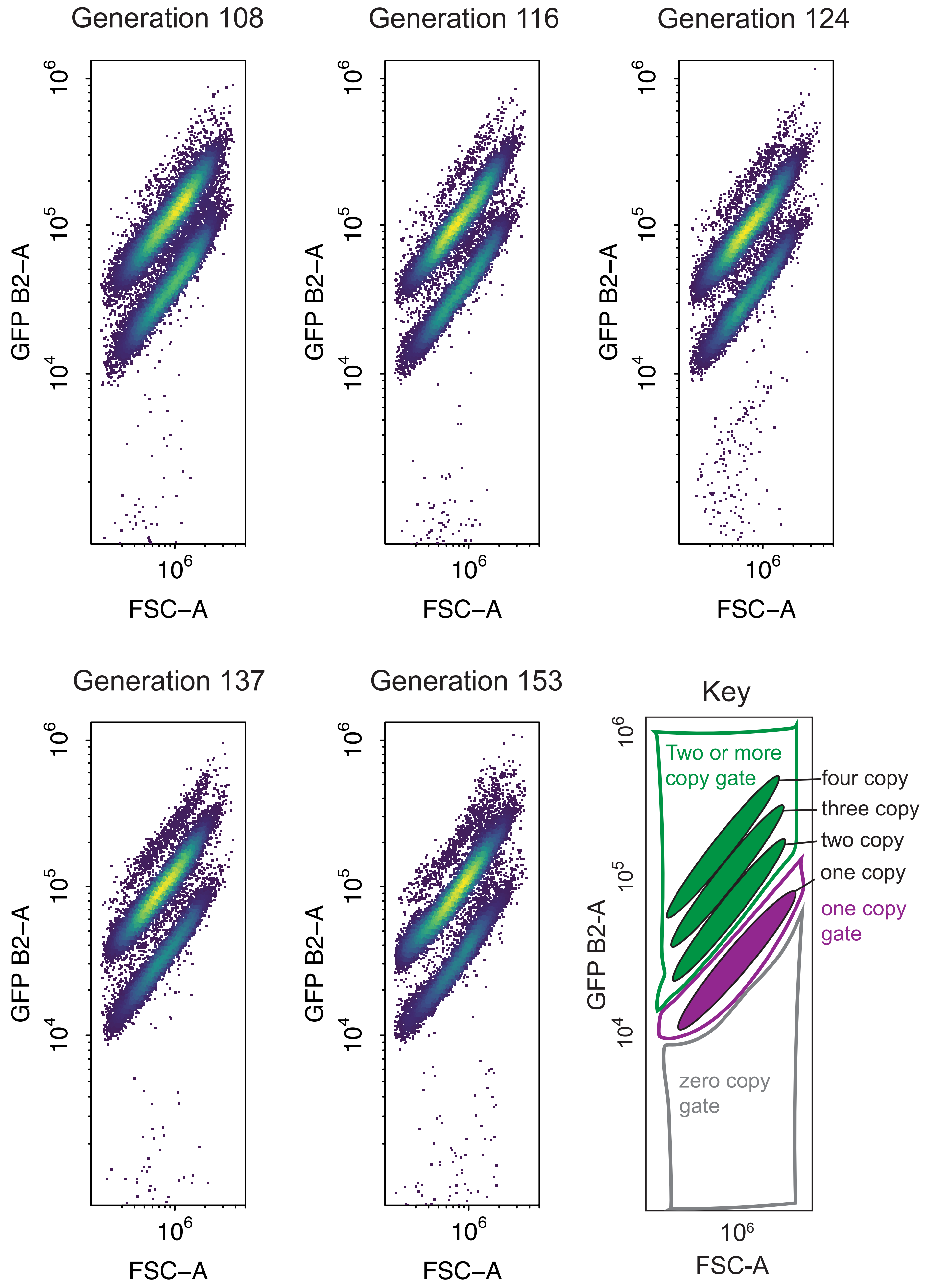

Wildtype population 2

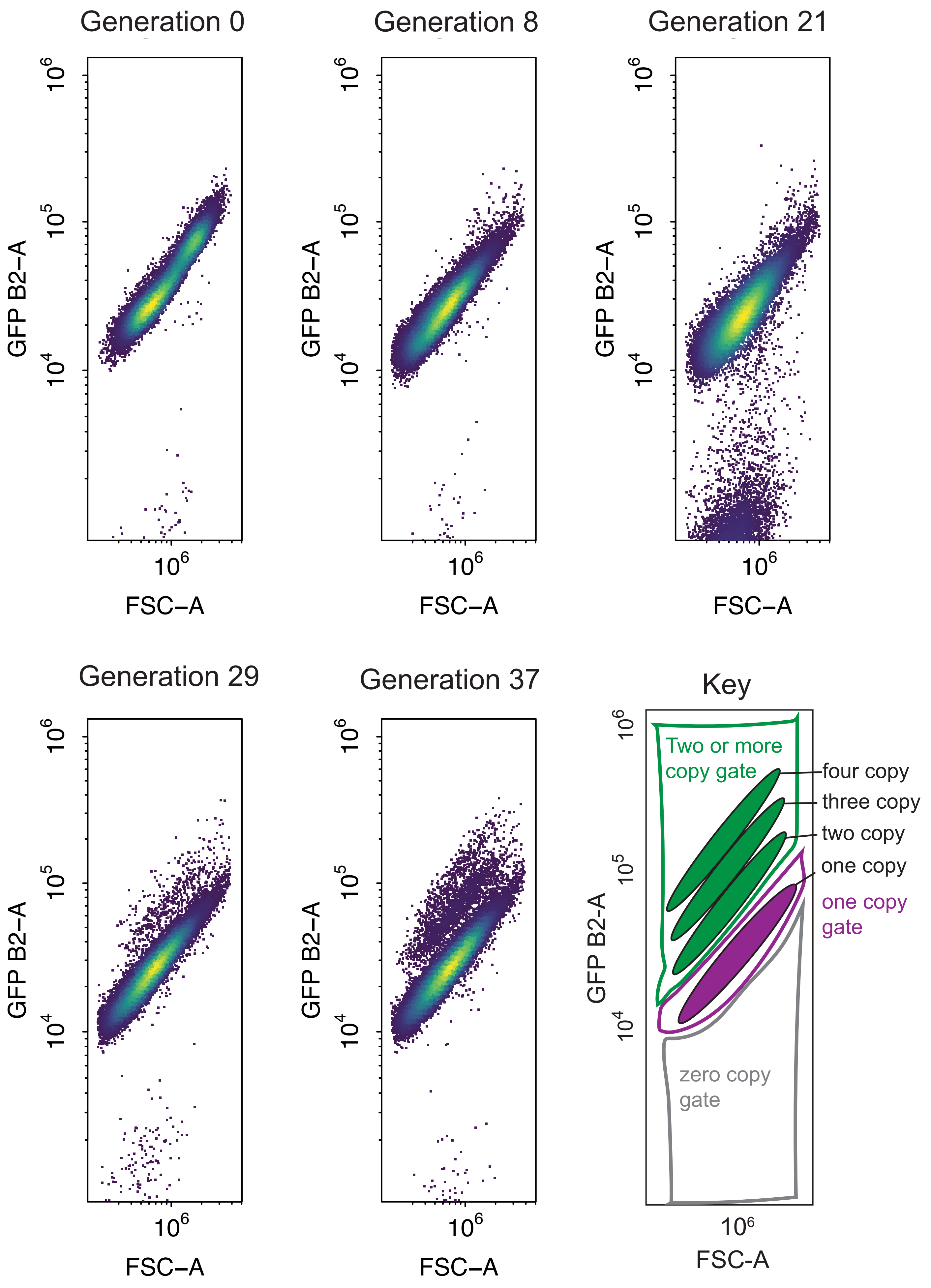

Wildtype population 2

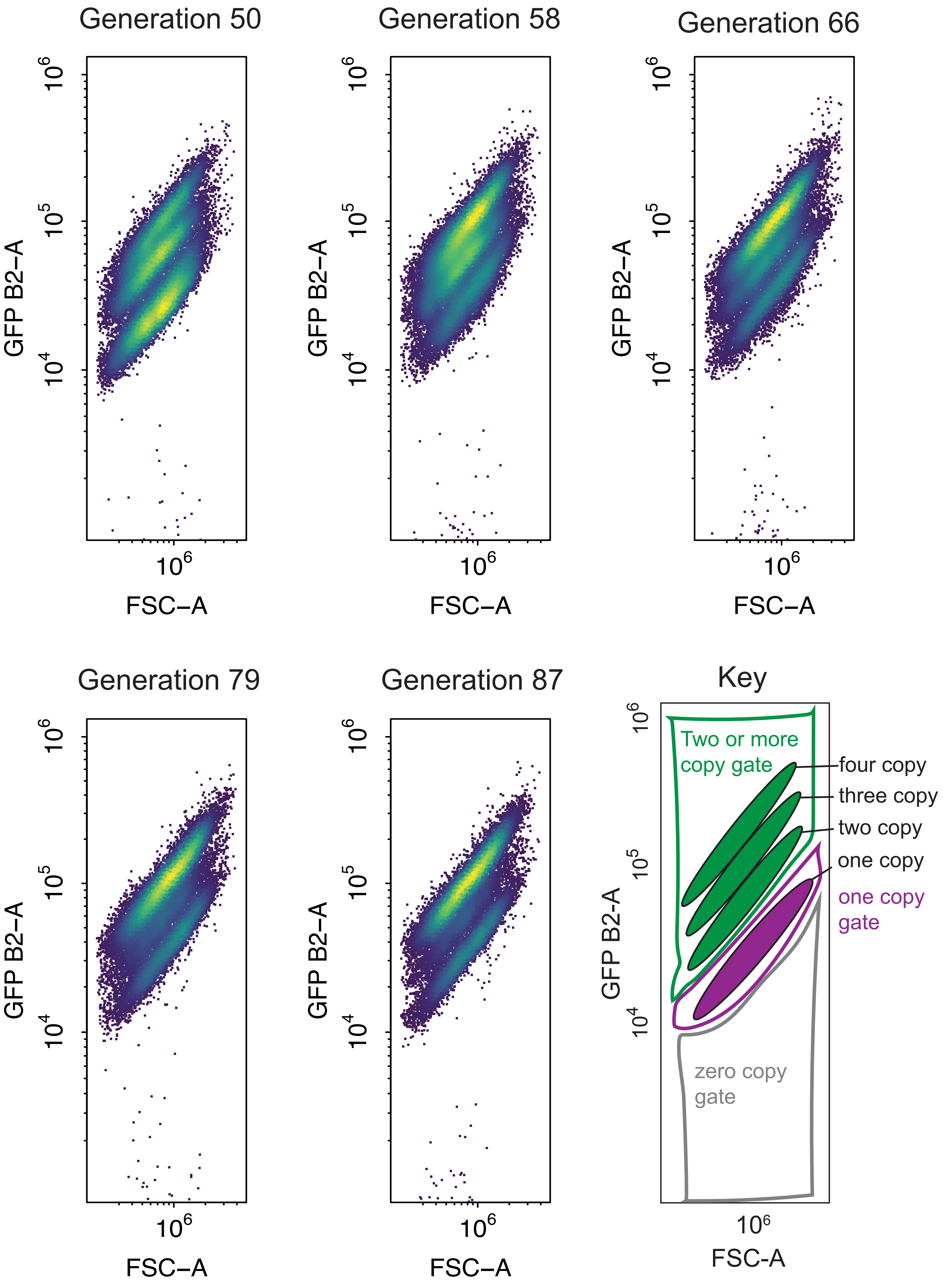

Wildtype population 2

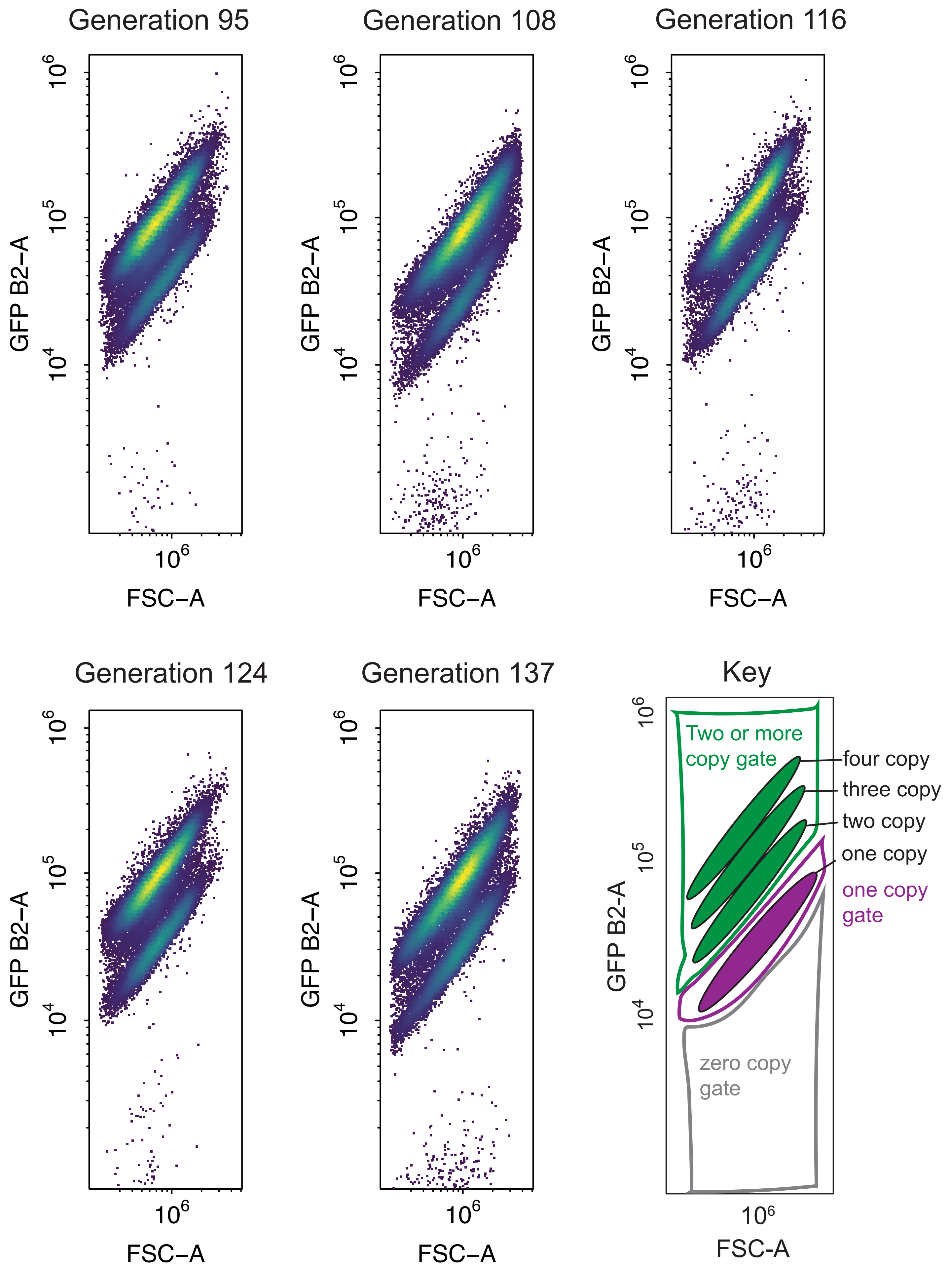

Wildtype population 3

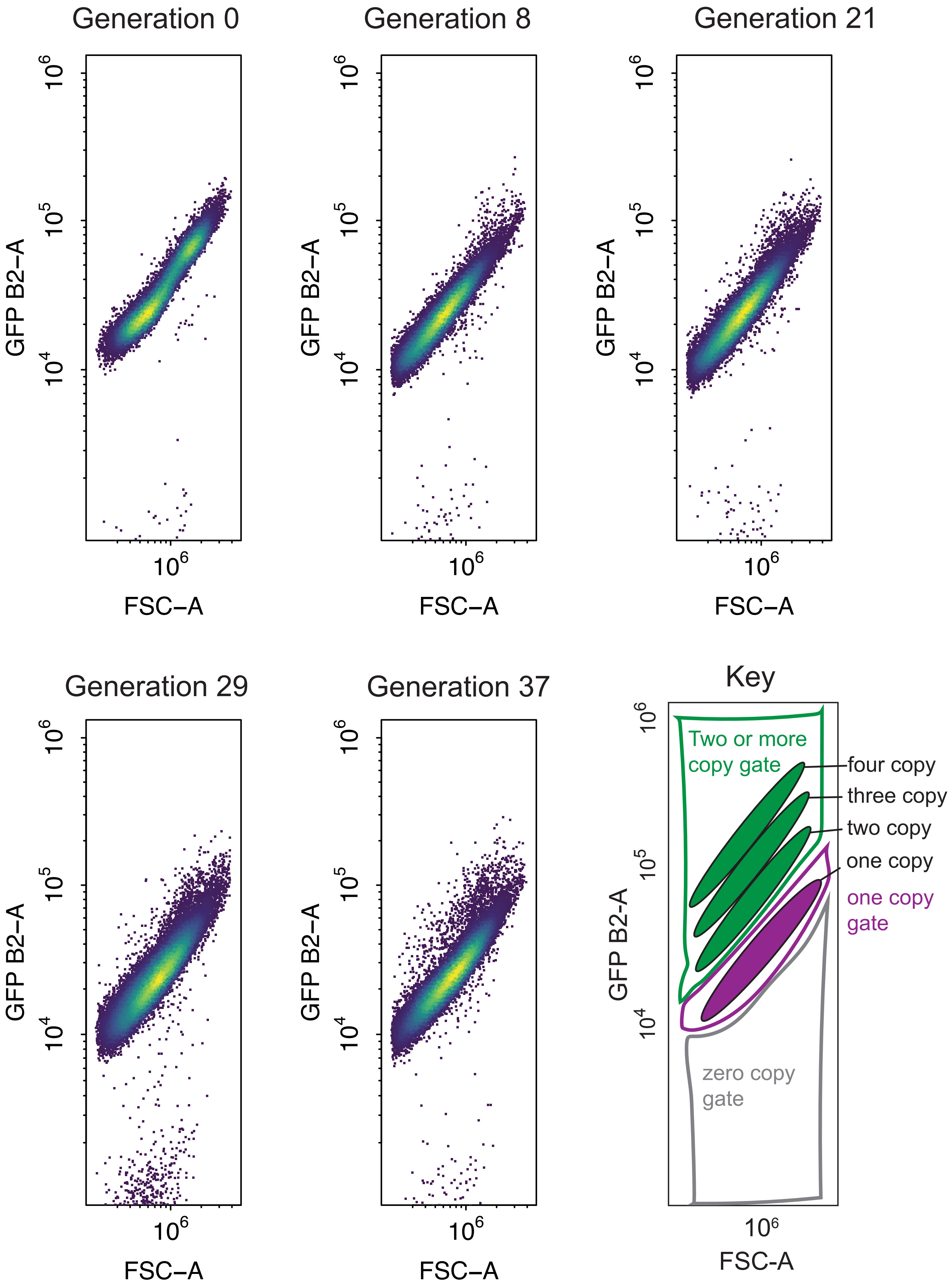

Wildtype population 3

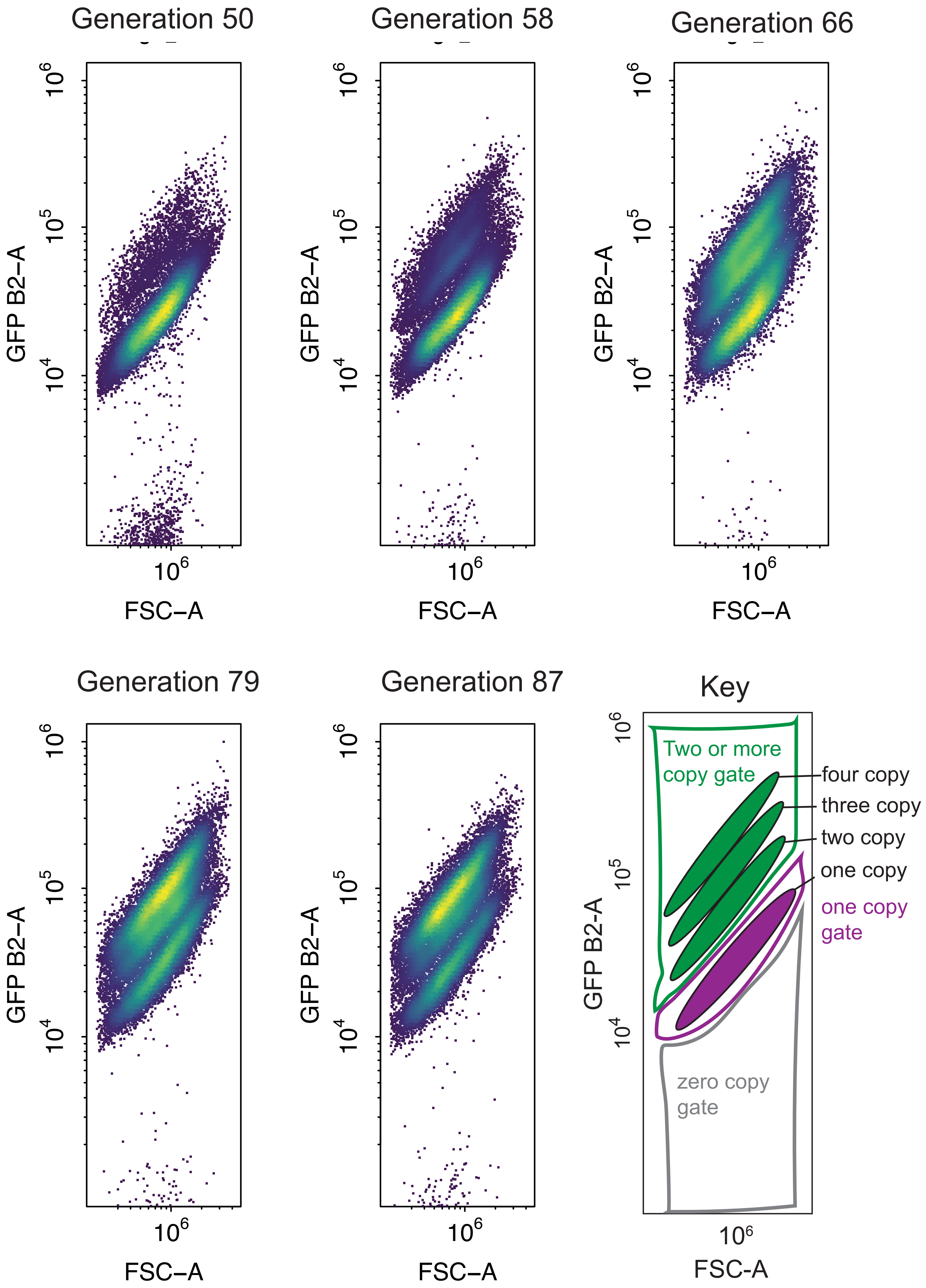

Wildtype population 3

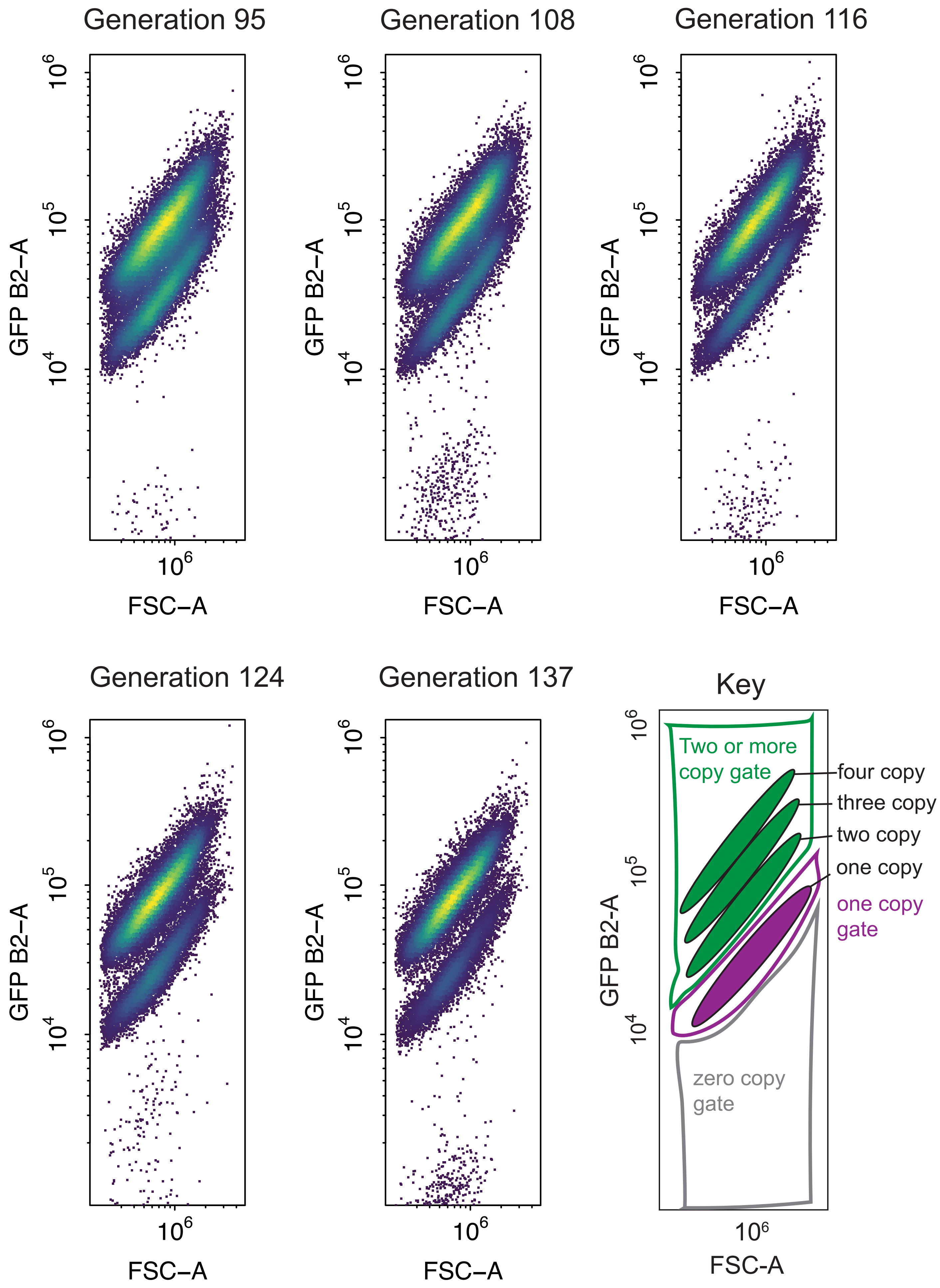

Wildtype population 4

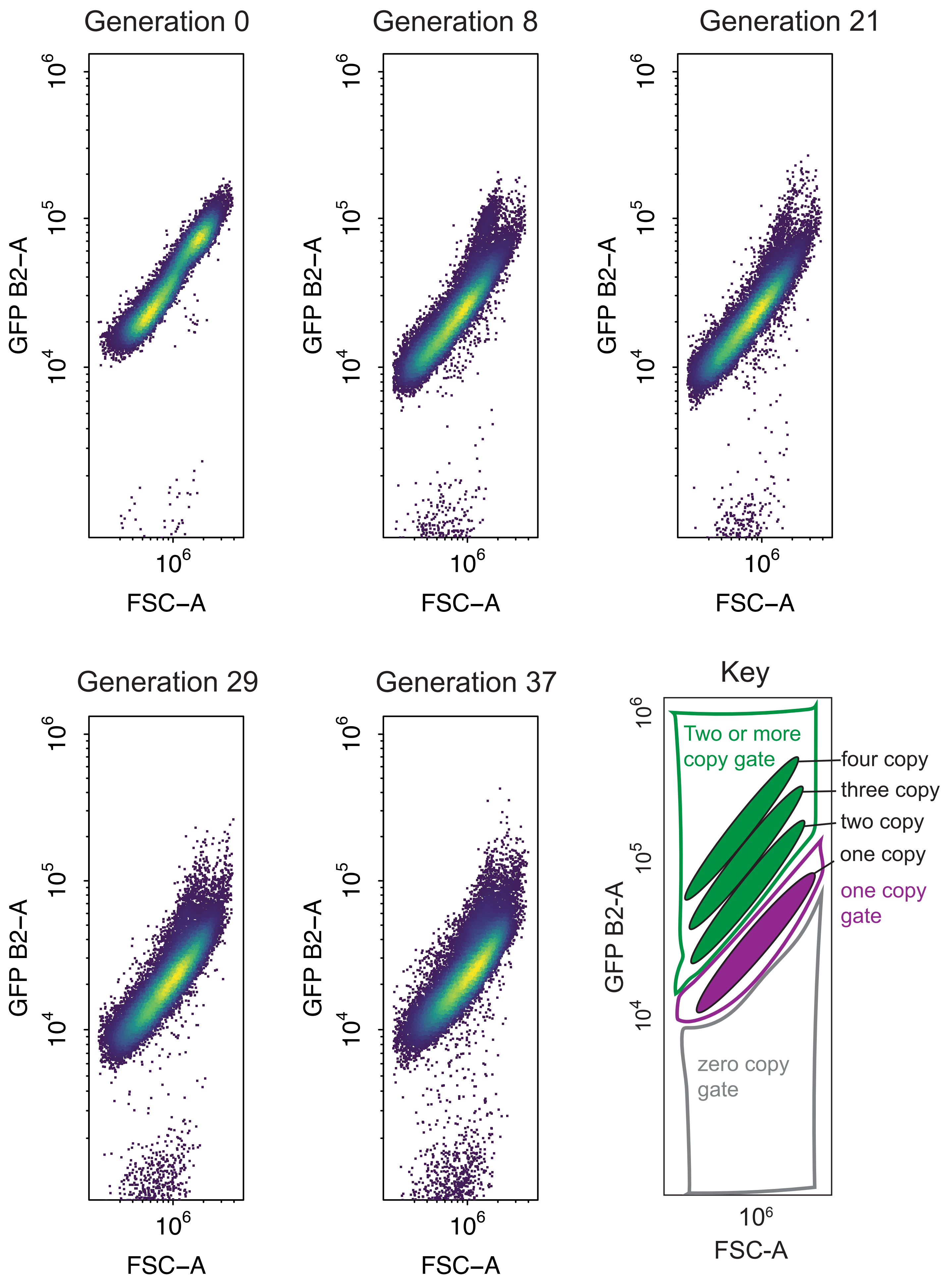

Wildtype population 4

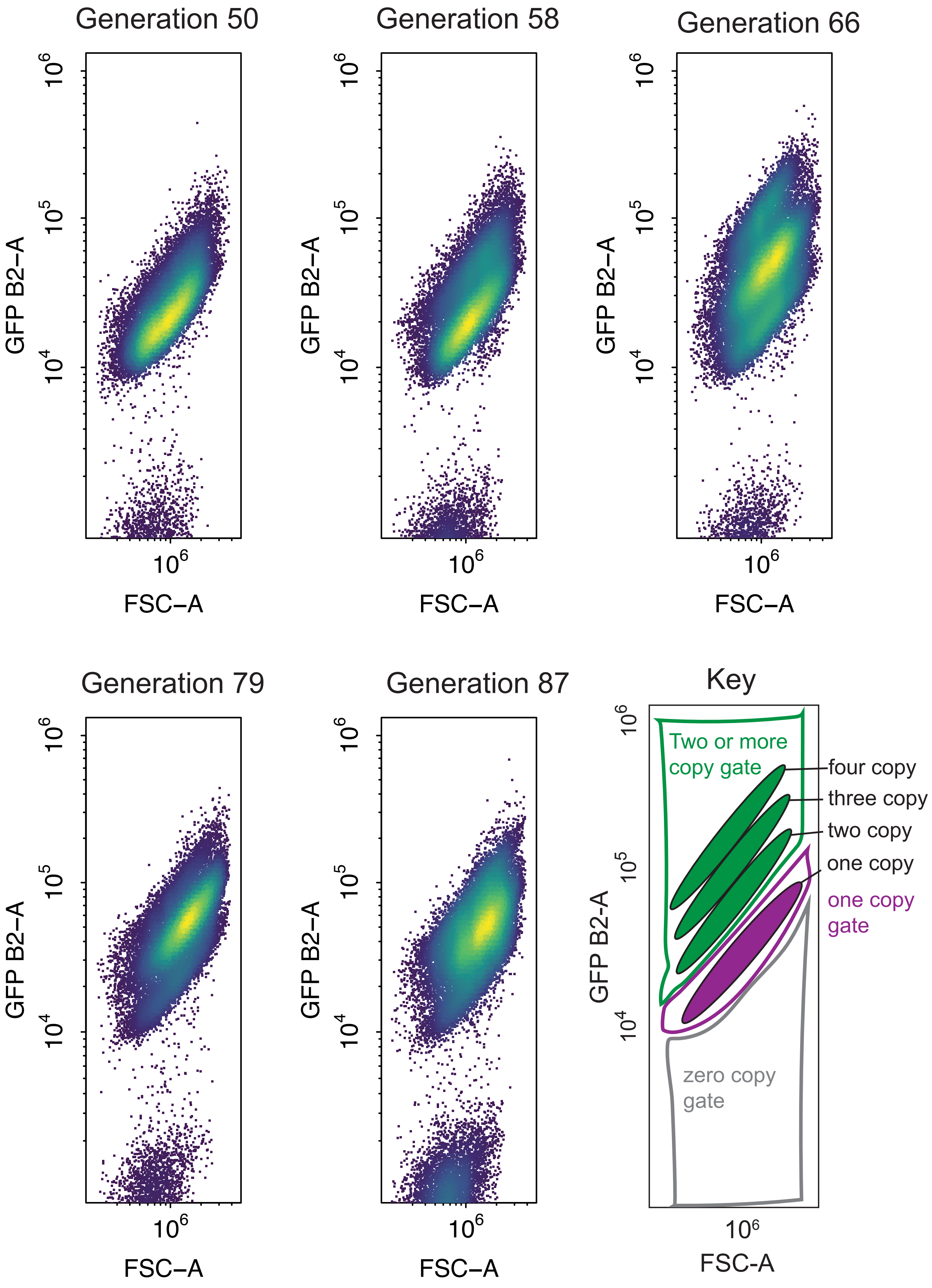

Wildtype population 4

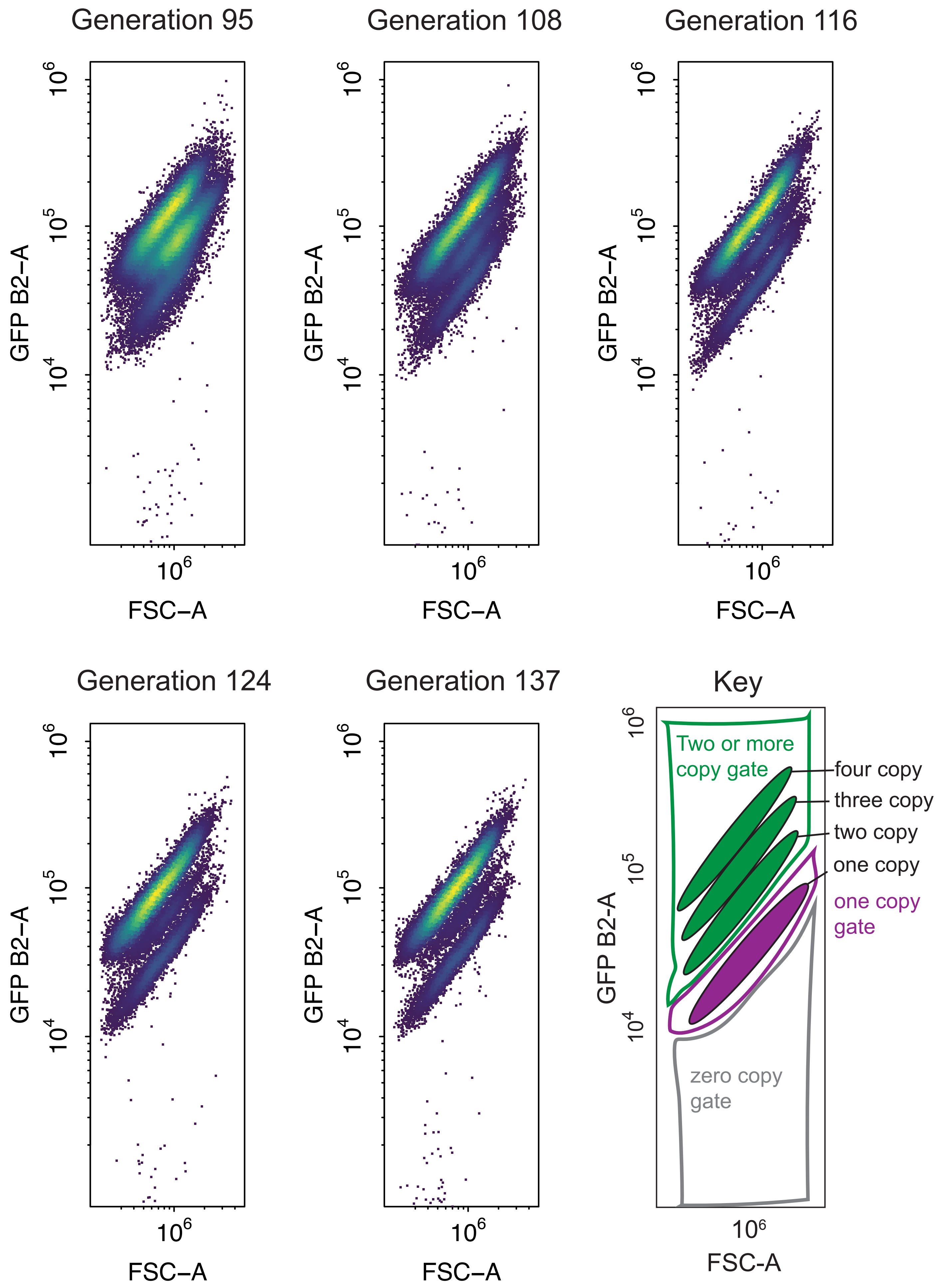

Wildtype population 5

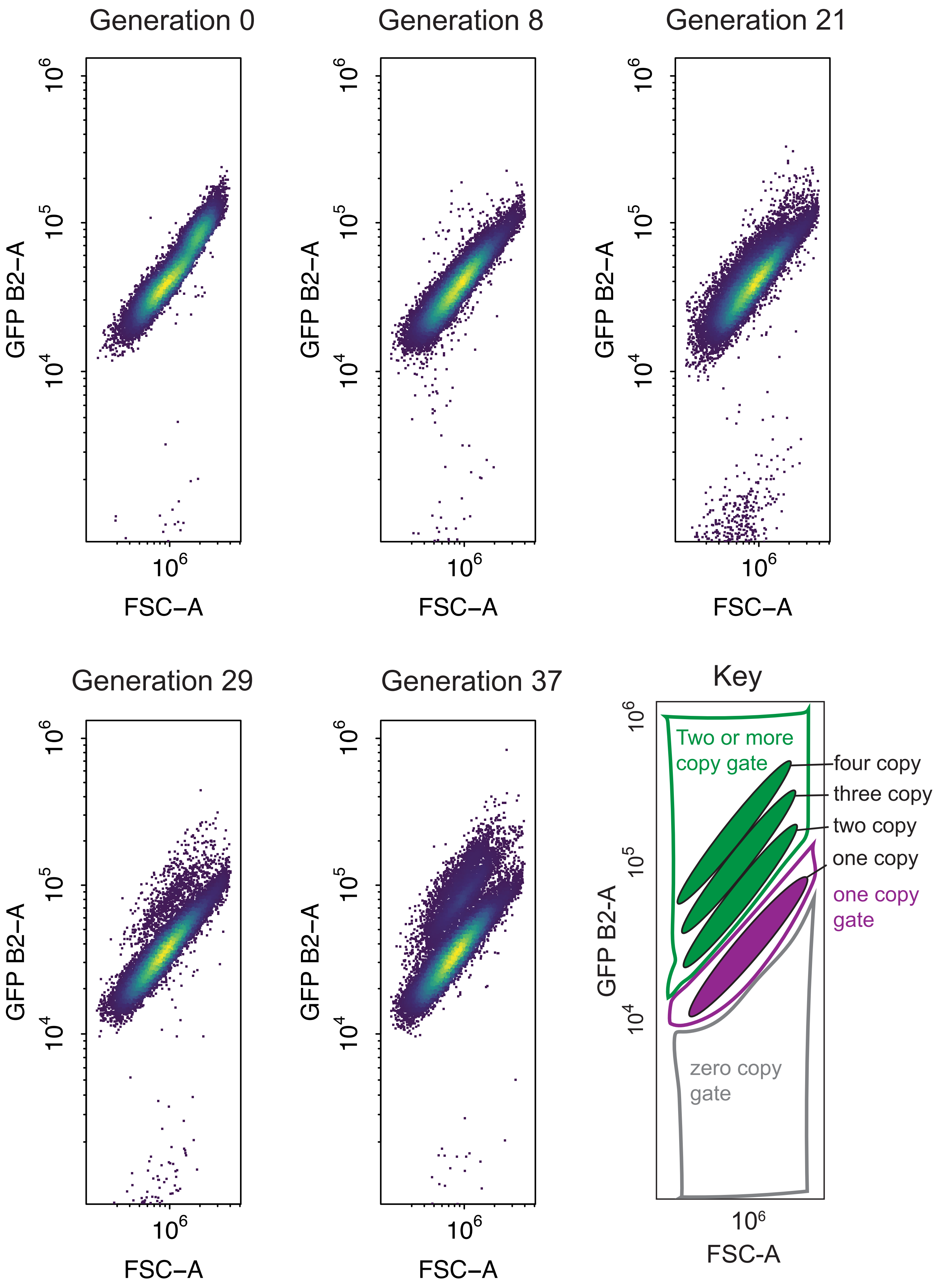

Wildtype population 5

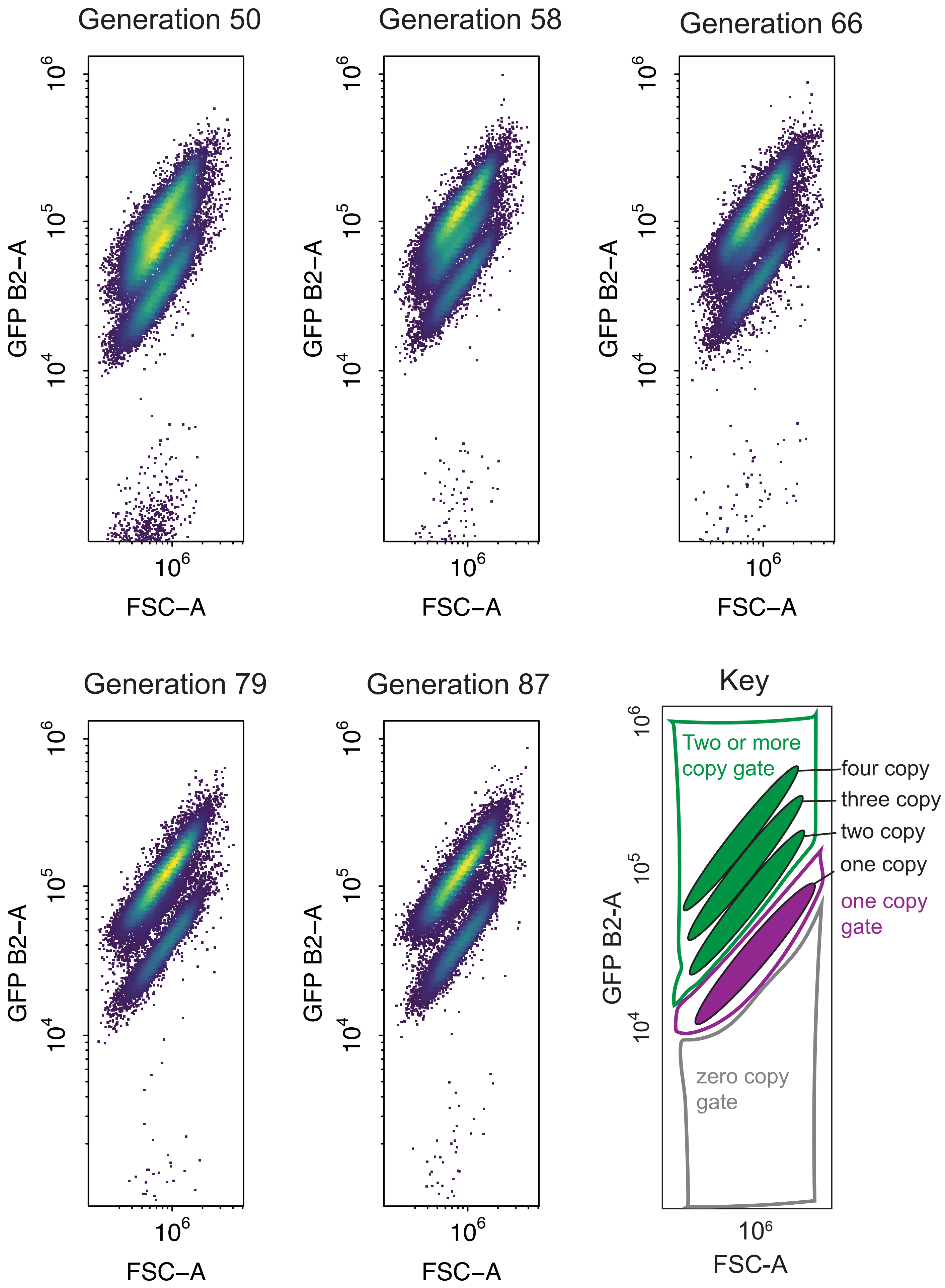

Wildtype population 5

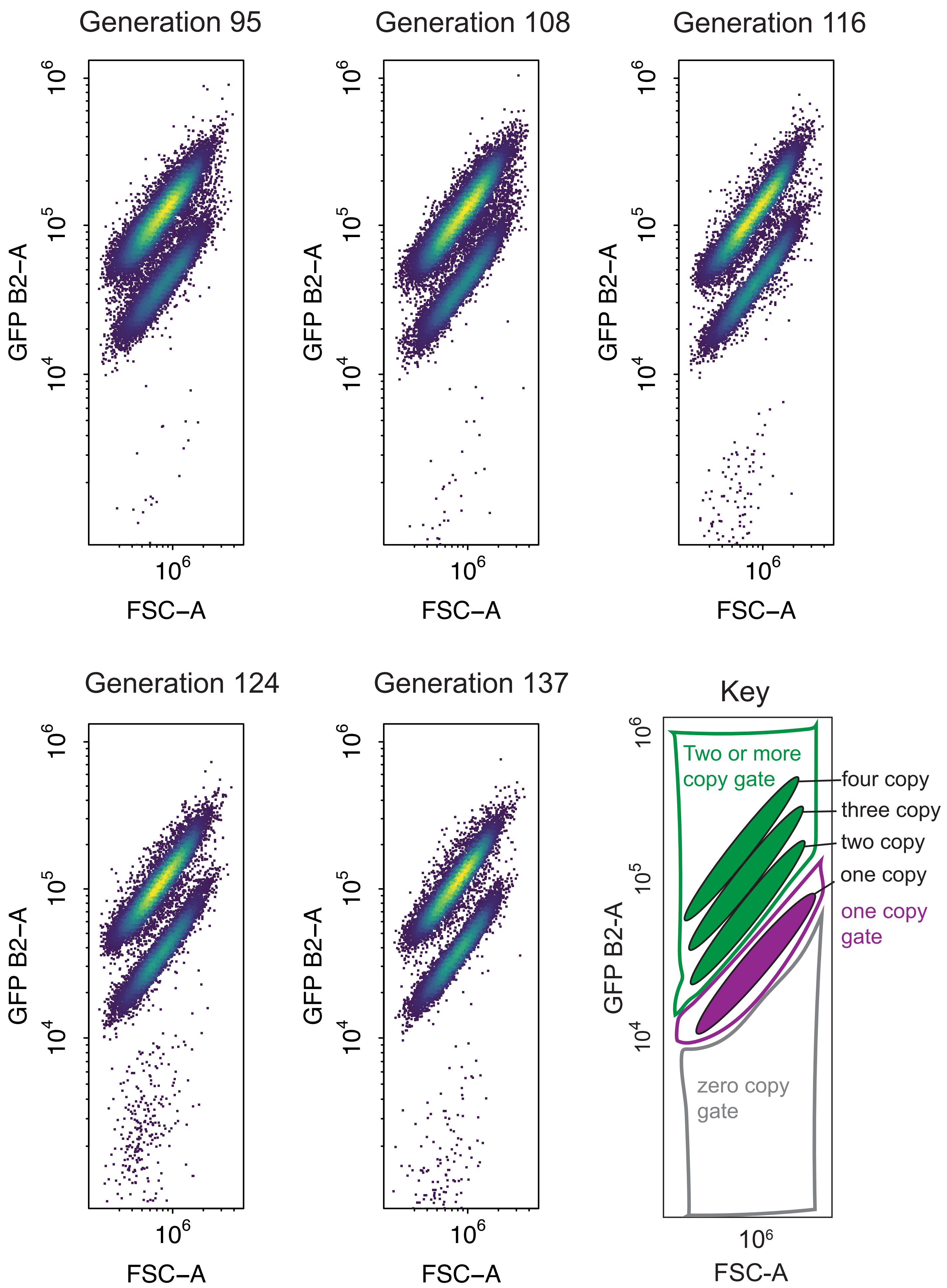

LTRΔ population 1

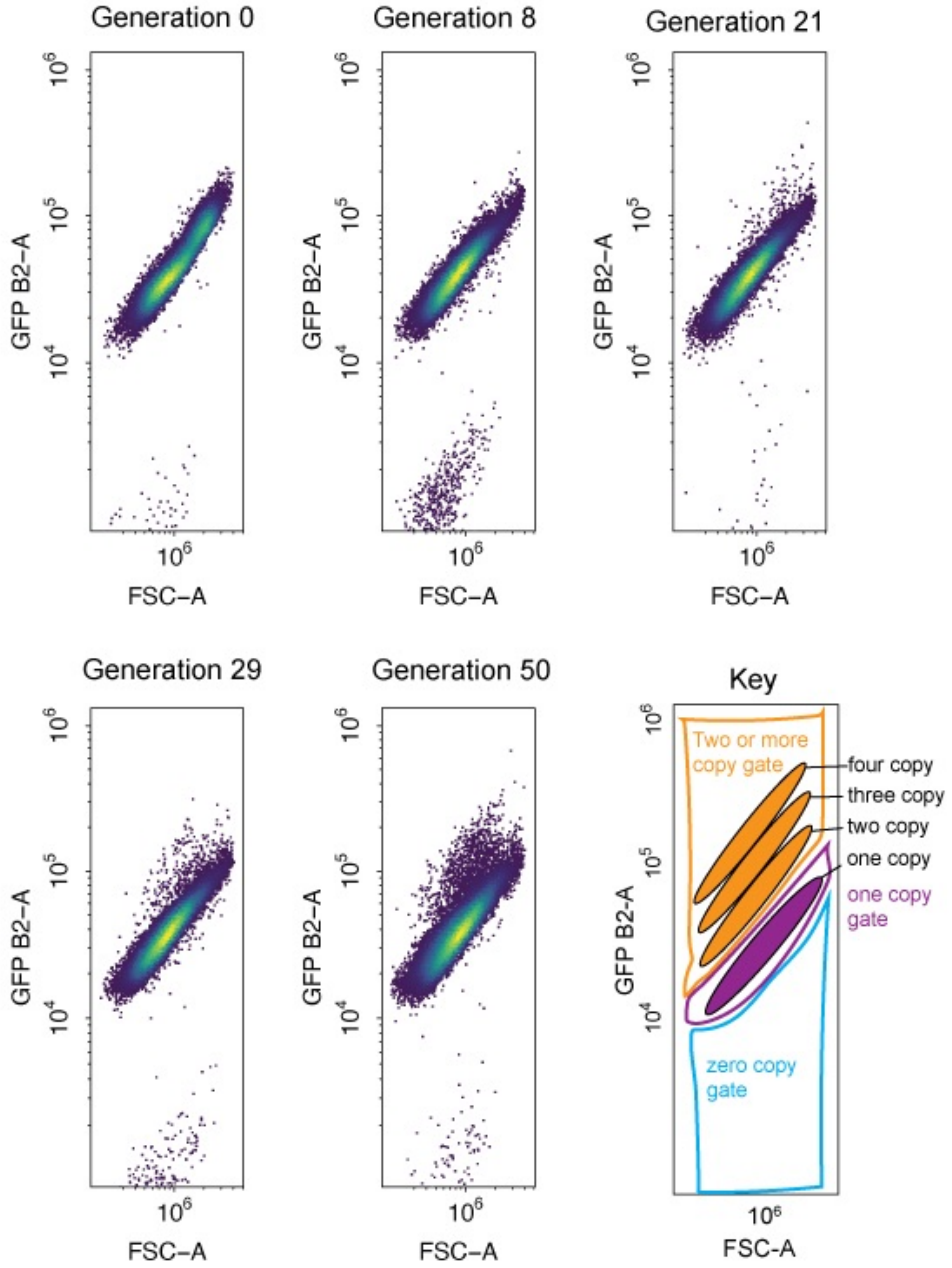

LTRΔ population 1

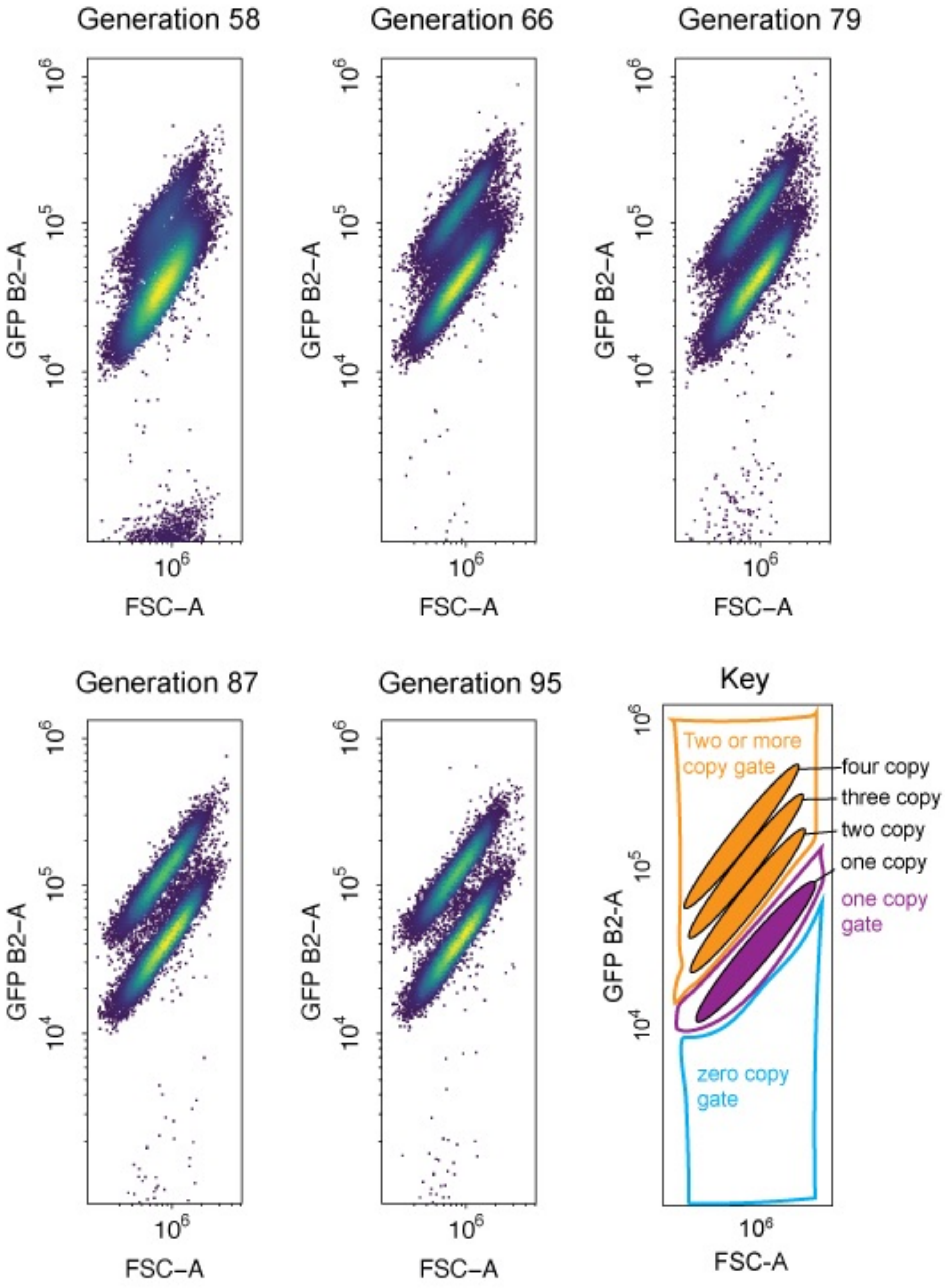

LTRΔ population 1

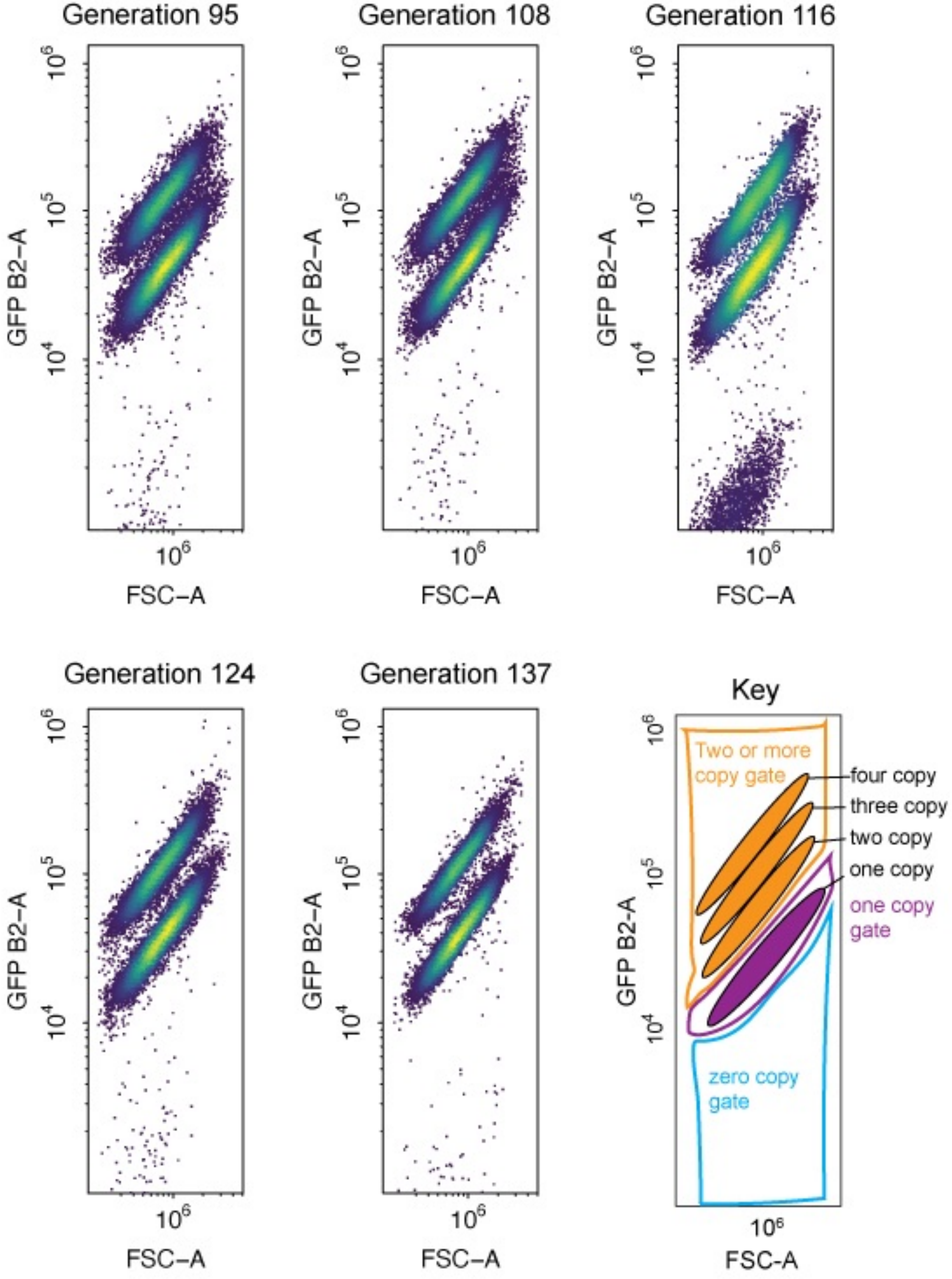

LTR∆ population 3

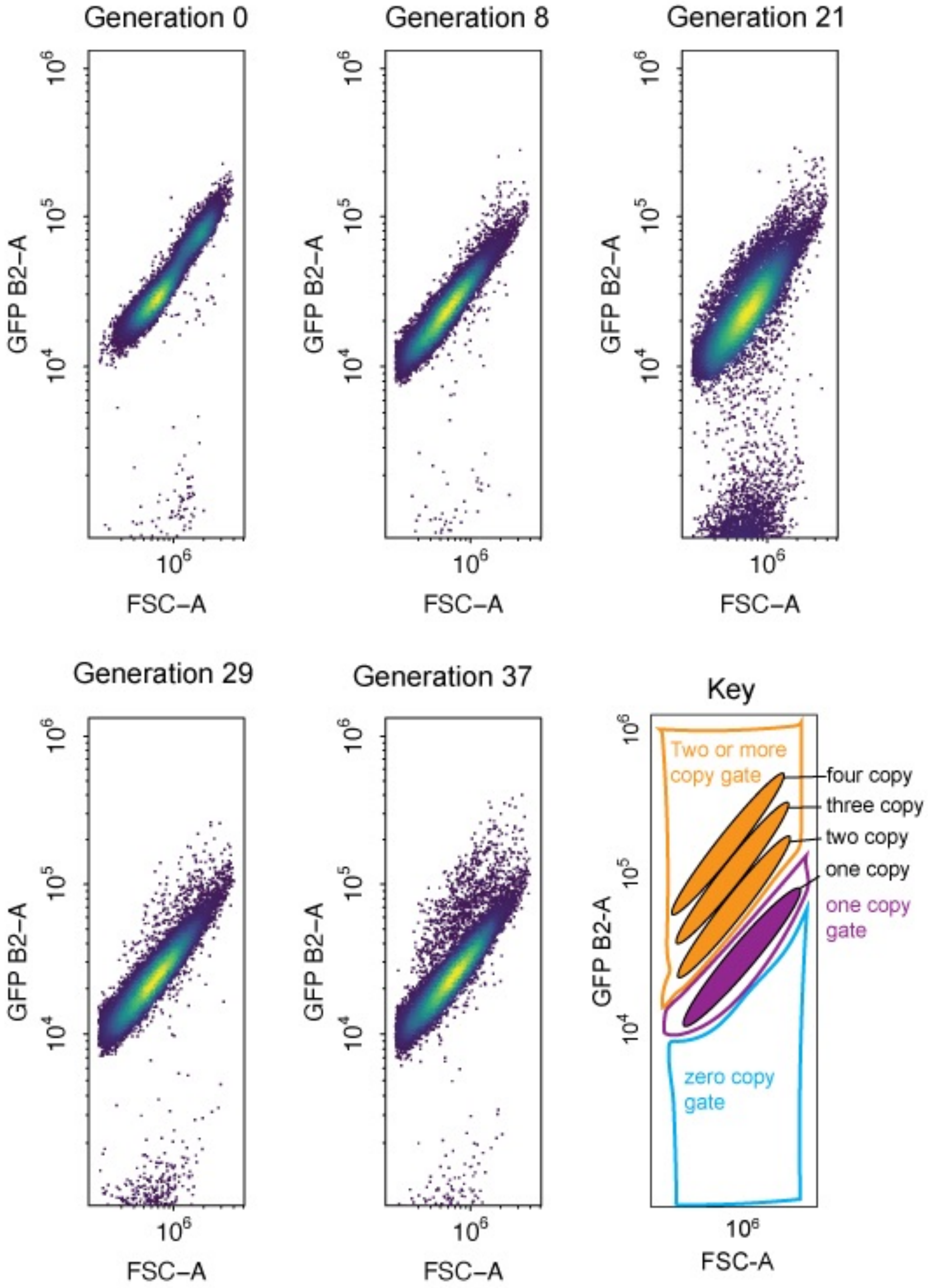

LTRΔ population 3

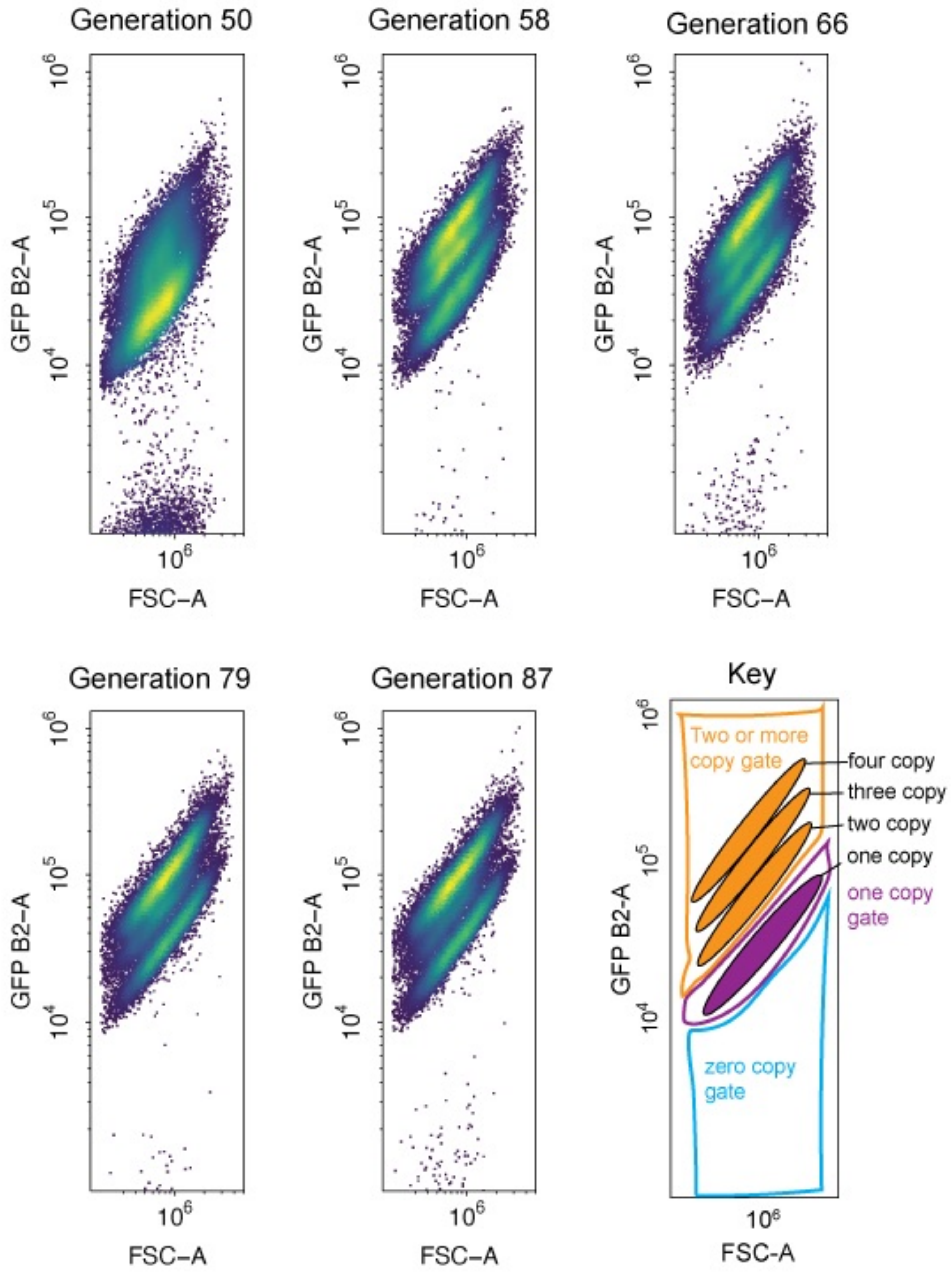

LTRΔ population 3

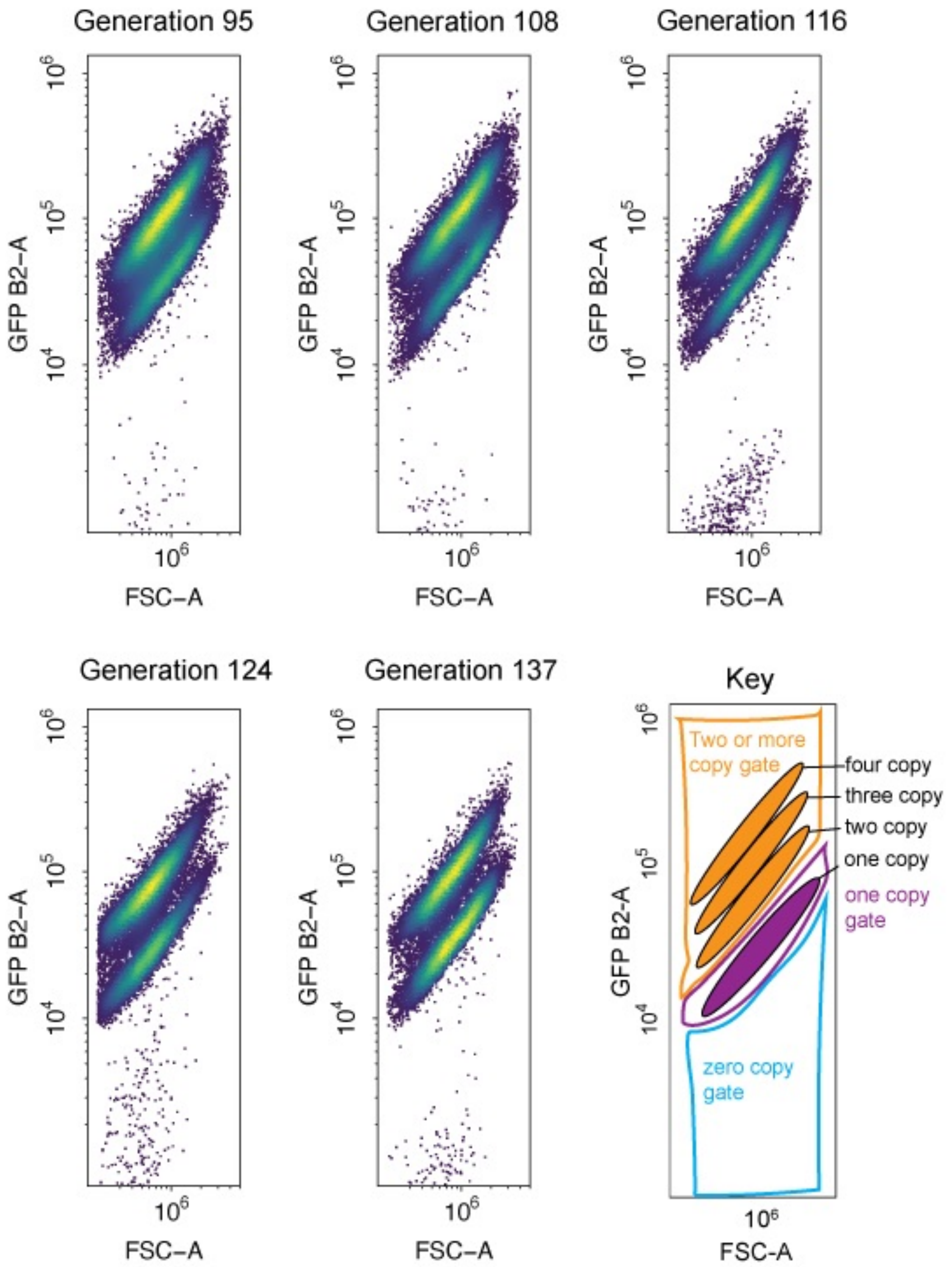

LTRΔ population 4

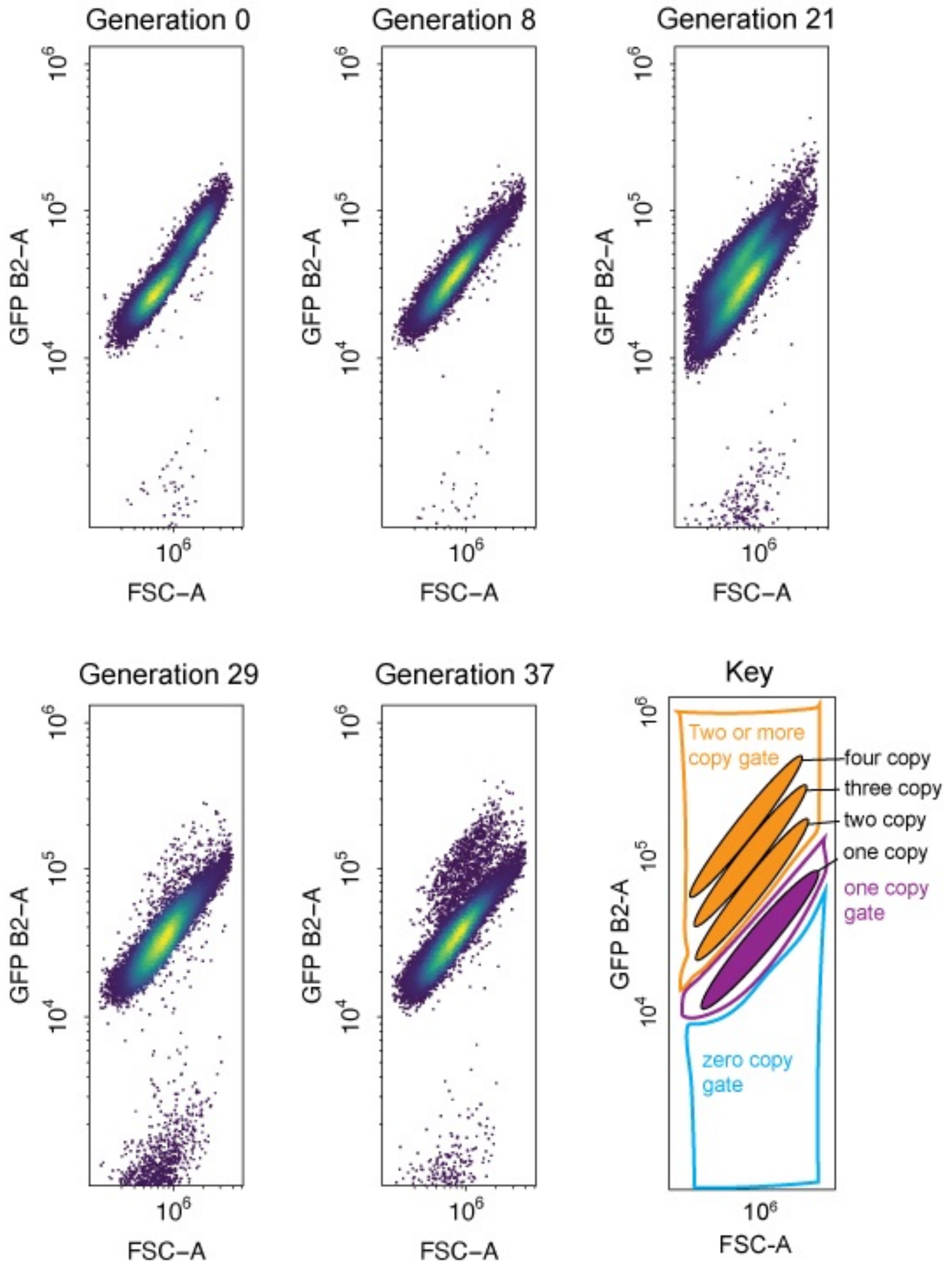

LTRΔ population 4

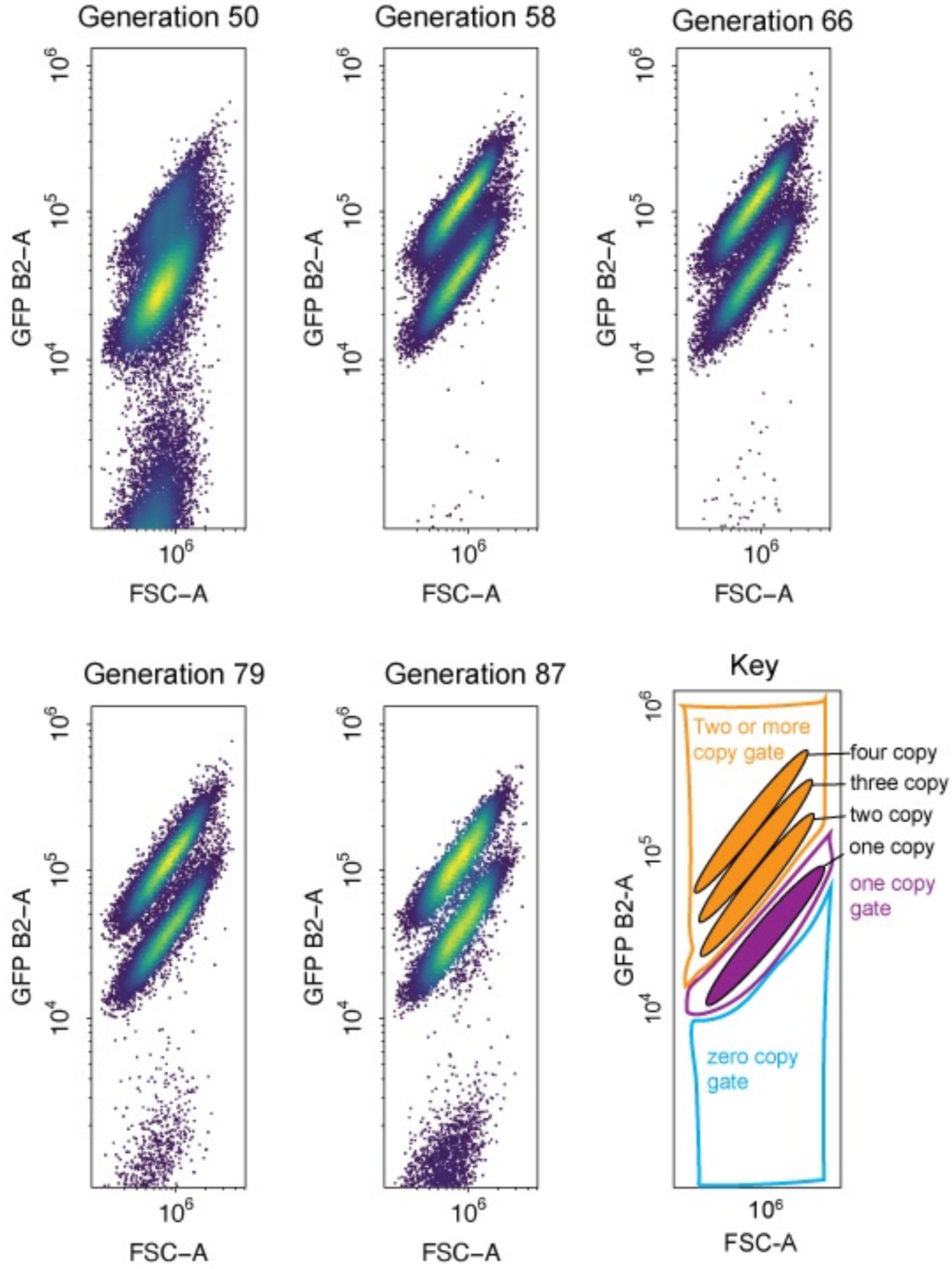

LTRΔ population 4

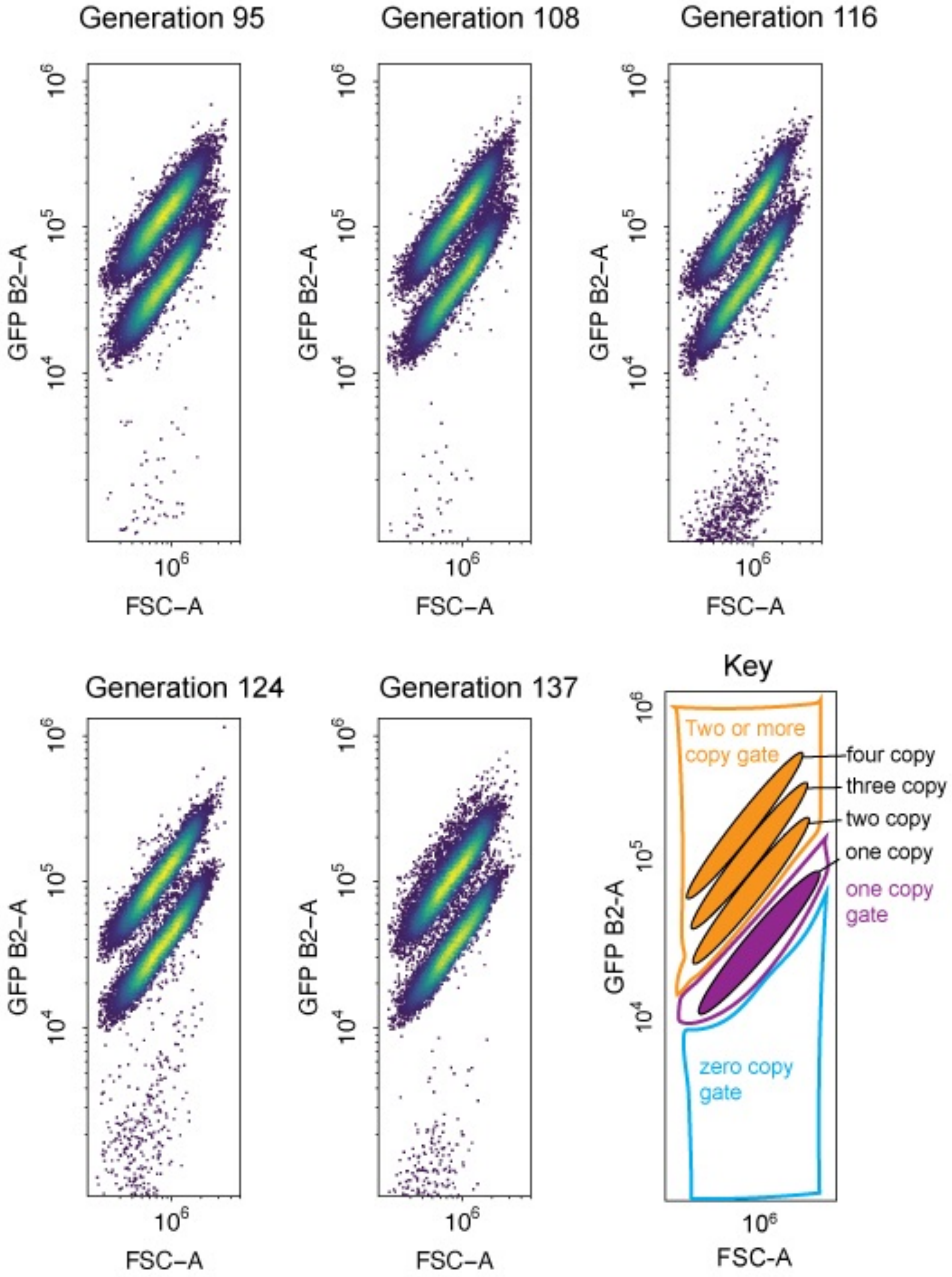

LTRΔ population 5

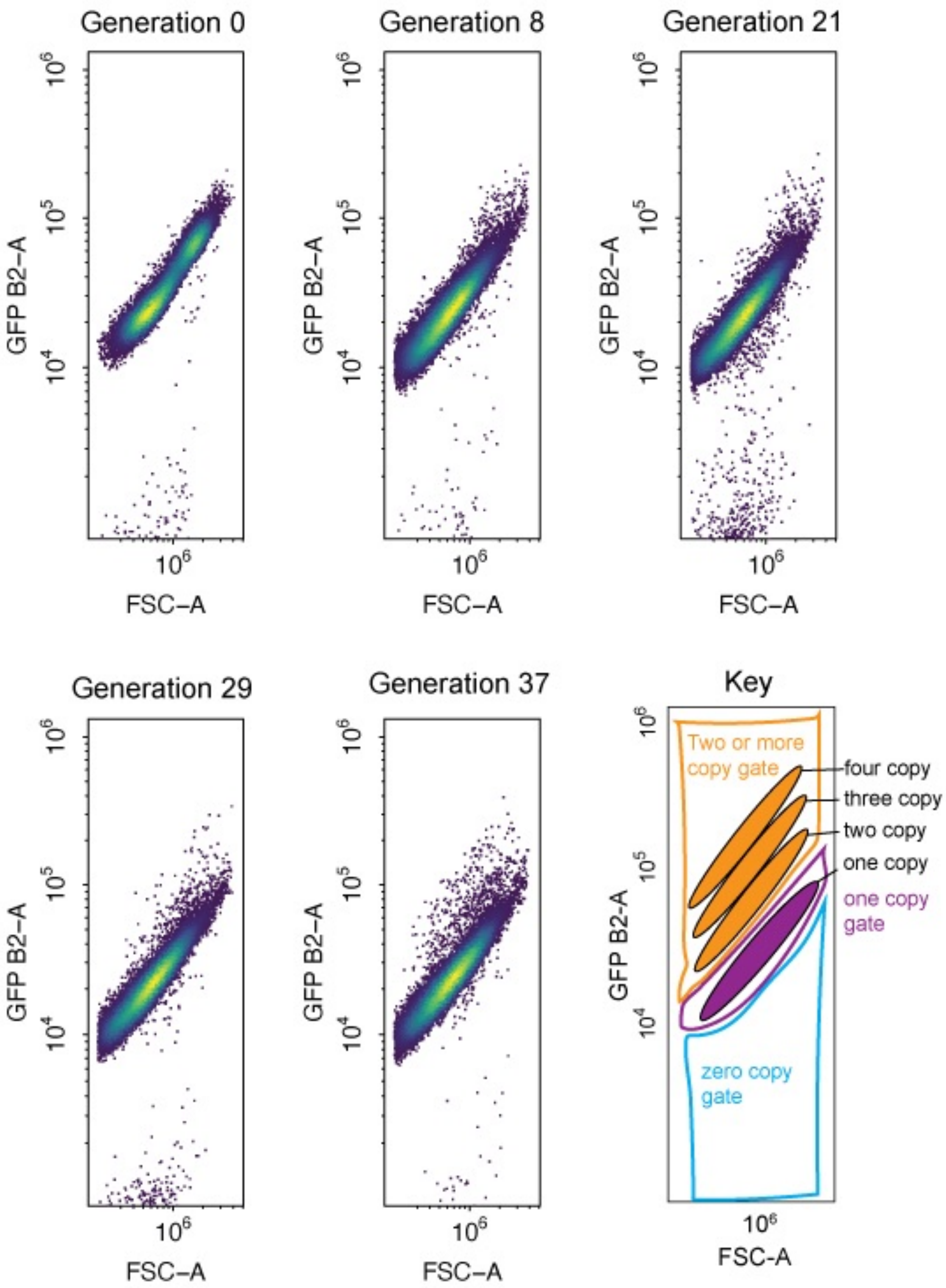

LTRΔ population 5

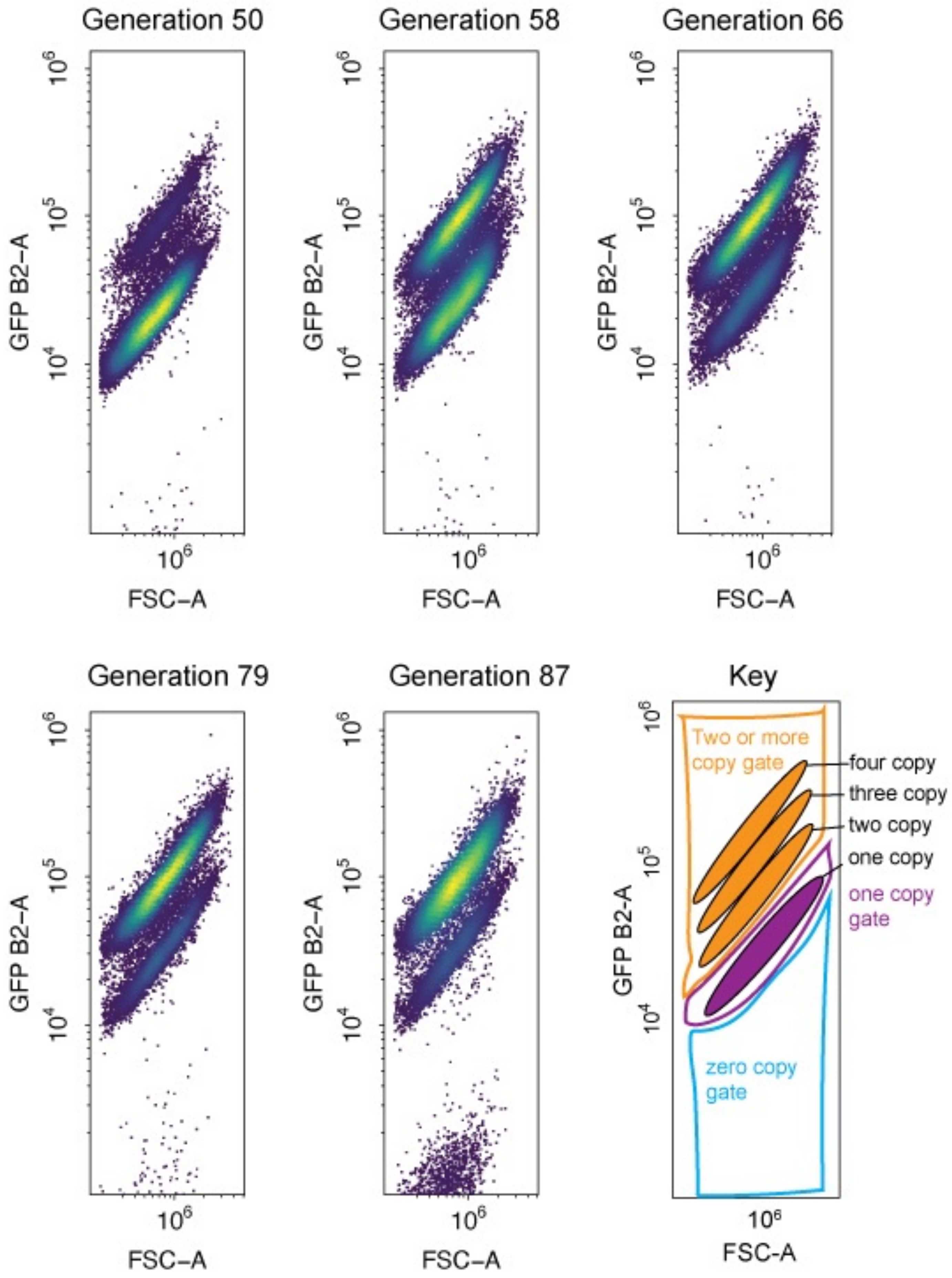

LTRΔ population 5

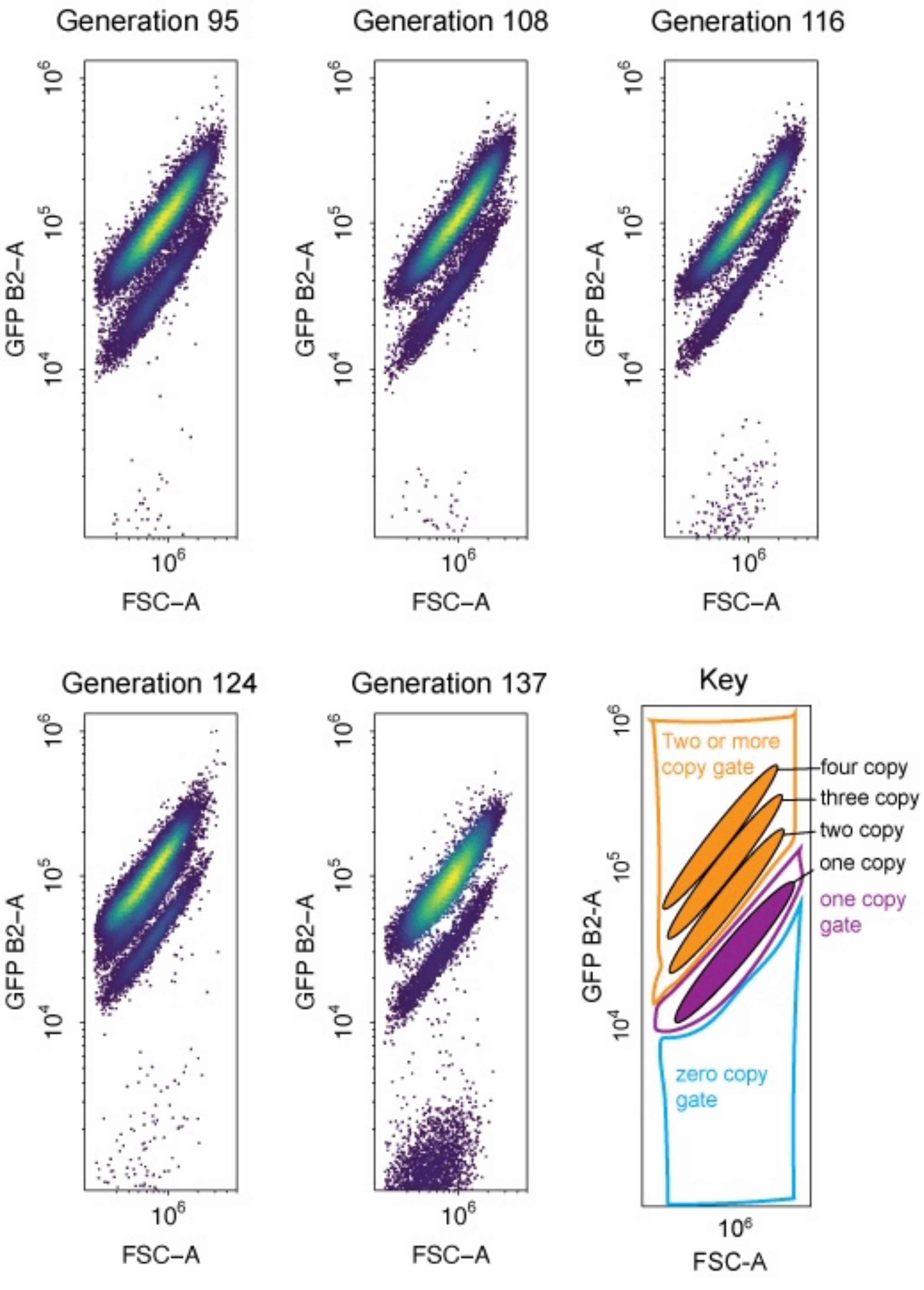

LTR∆ population 6

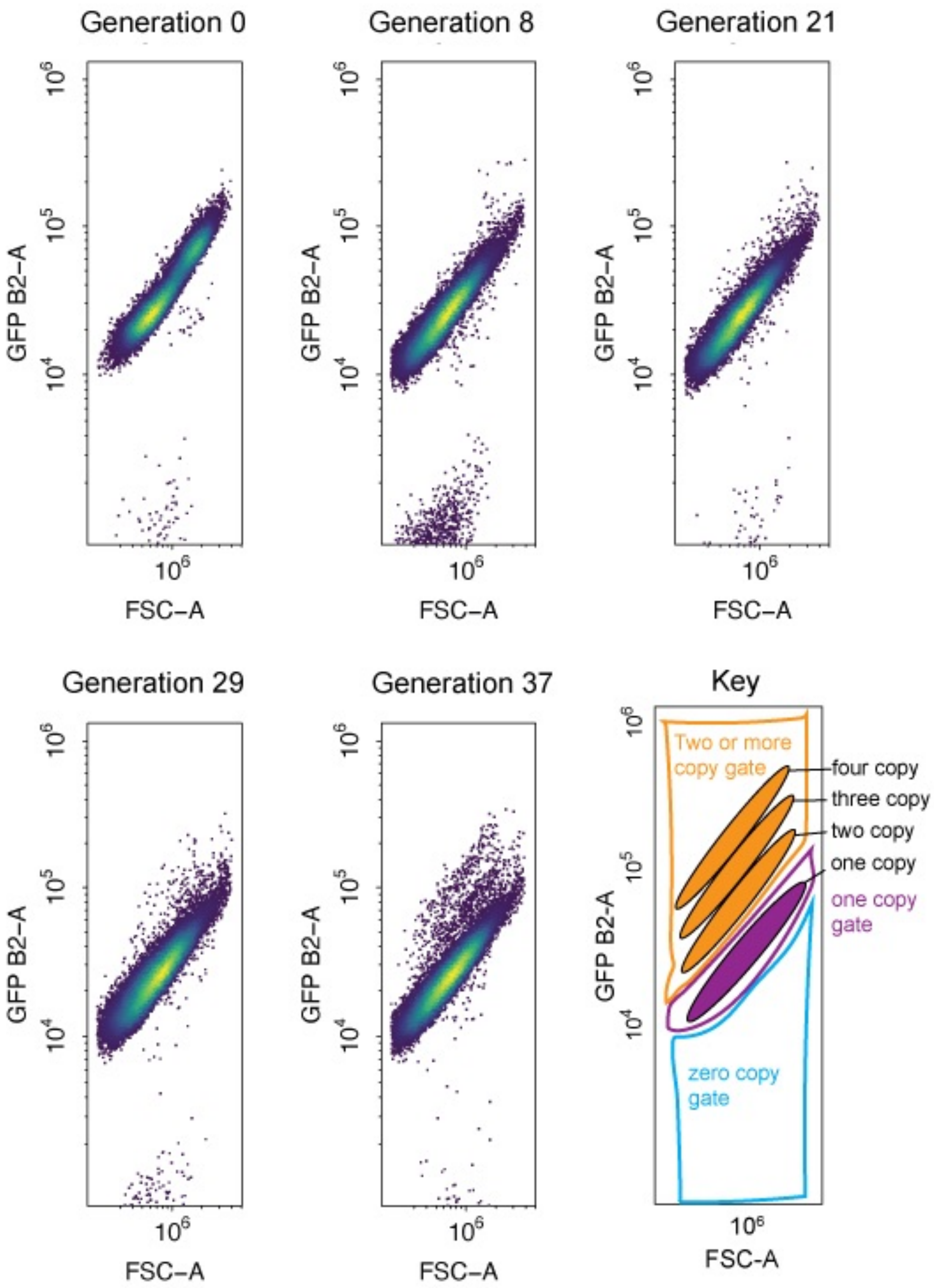

LTRΔ population 6

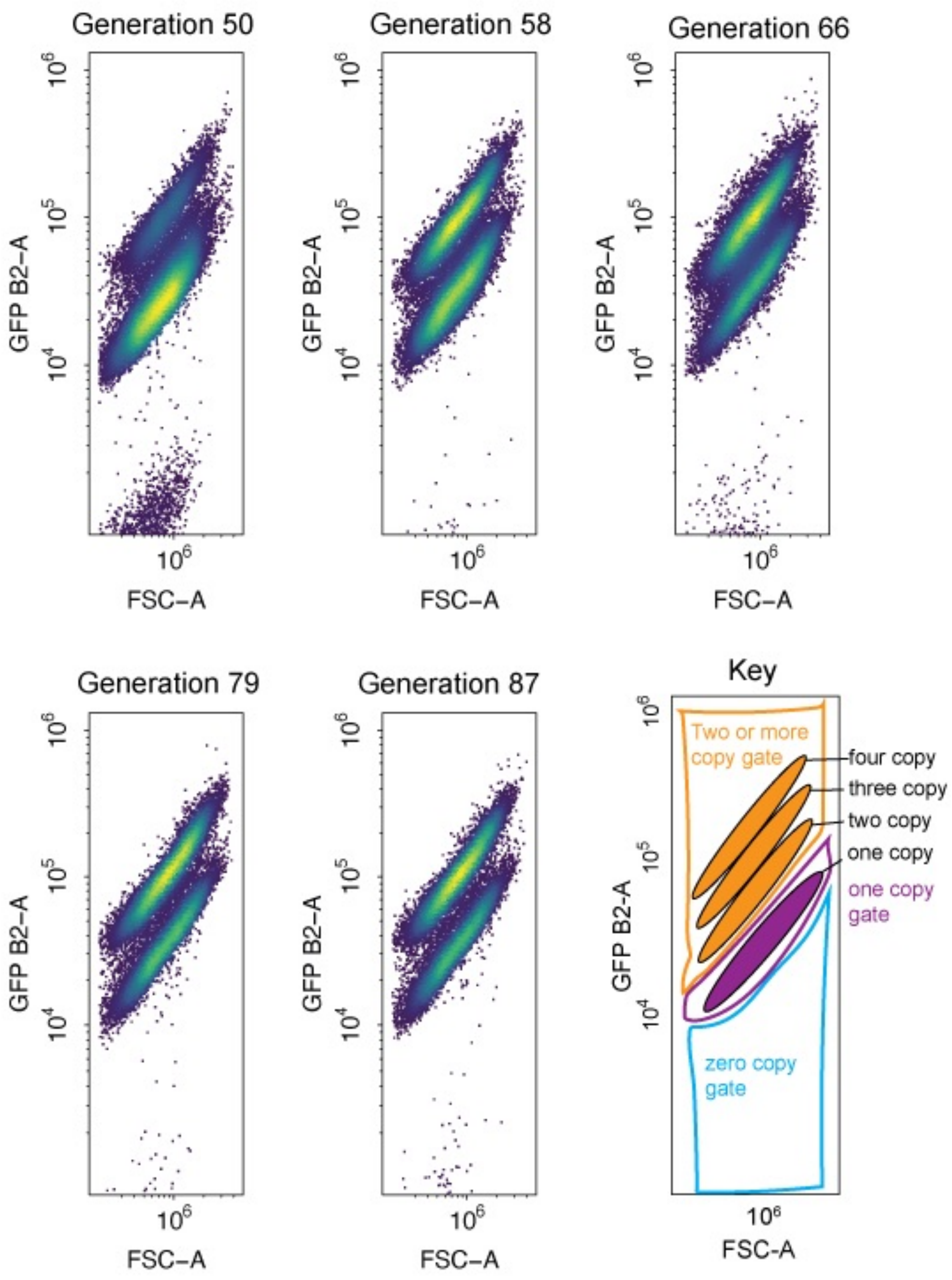

LTRΔ population 6

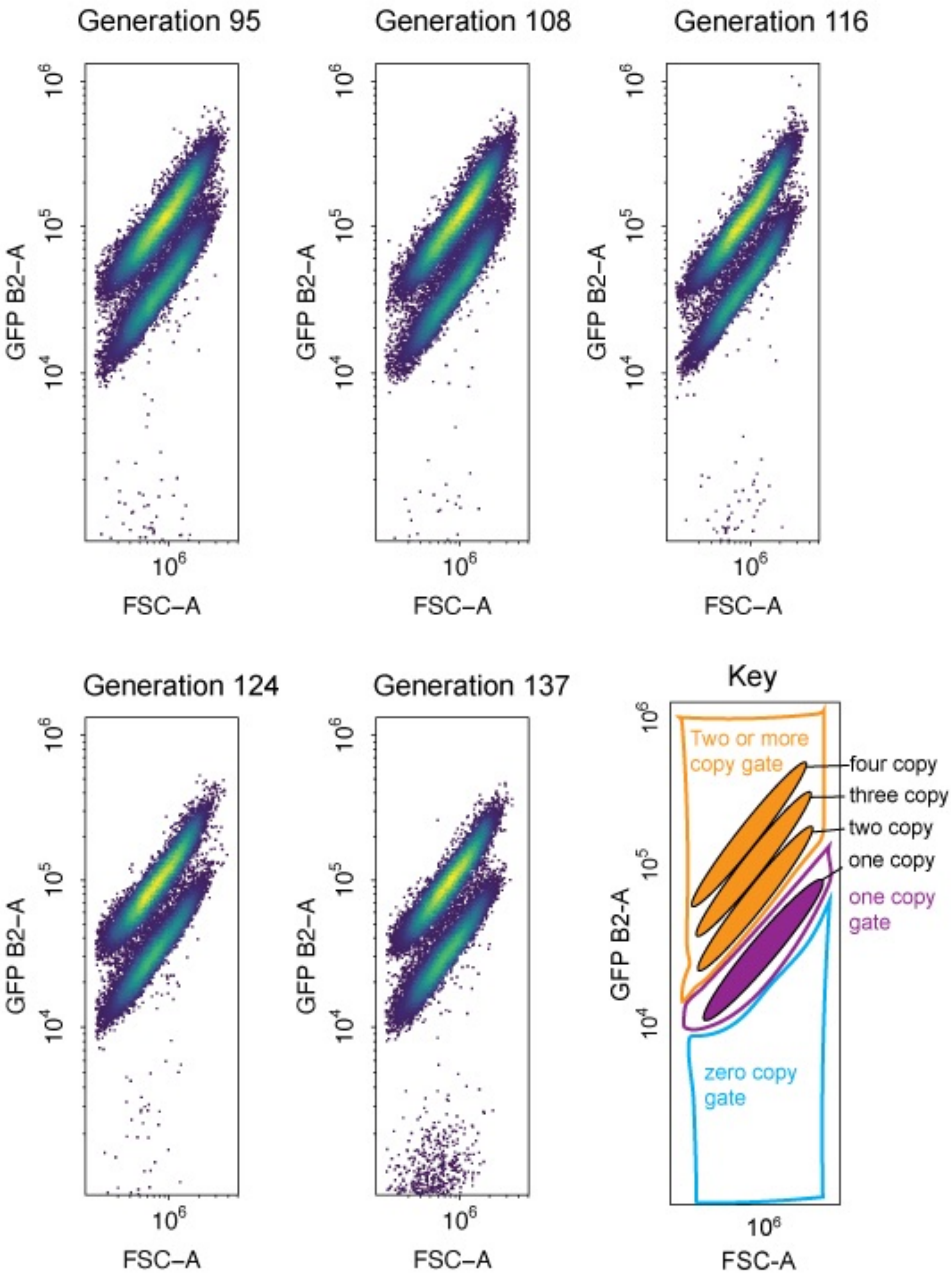

LTRΔ population 7

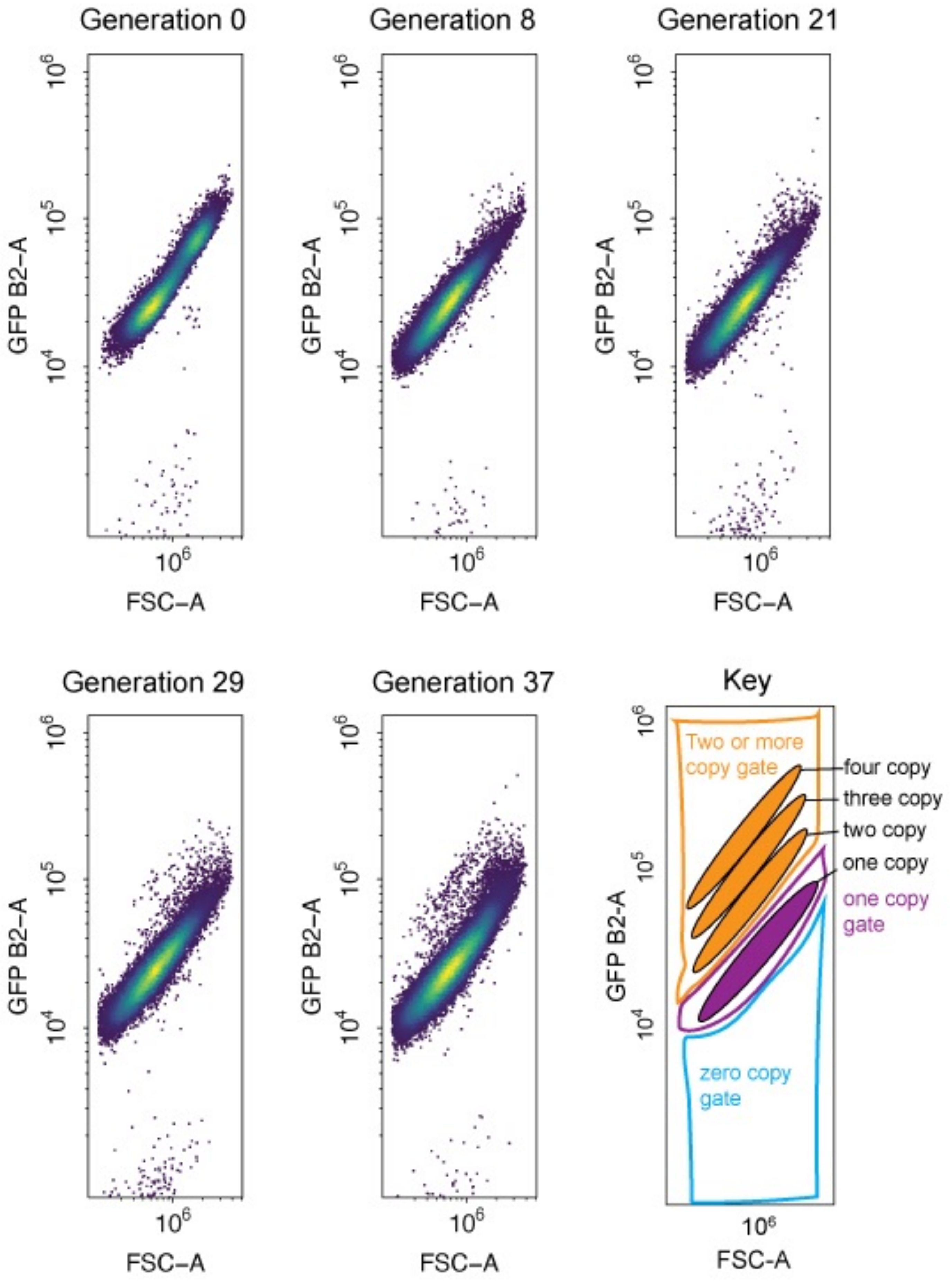

LTRΔ population 7

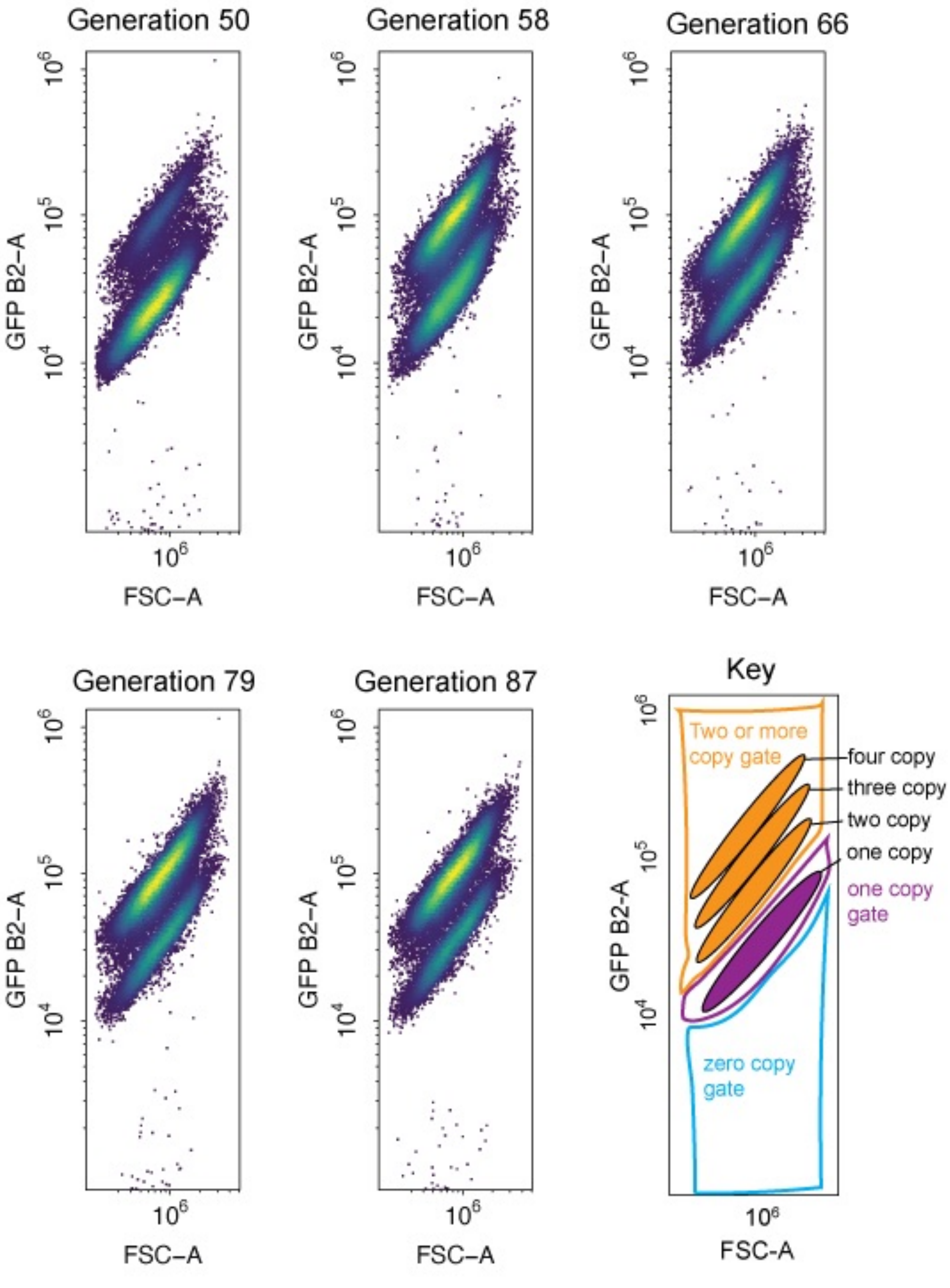

LTR∆ population 7

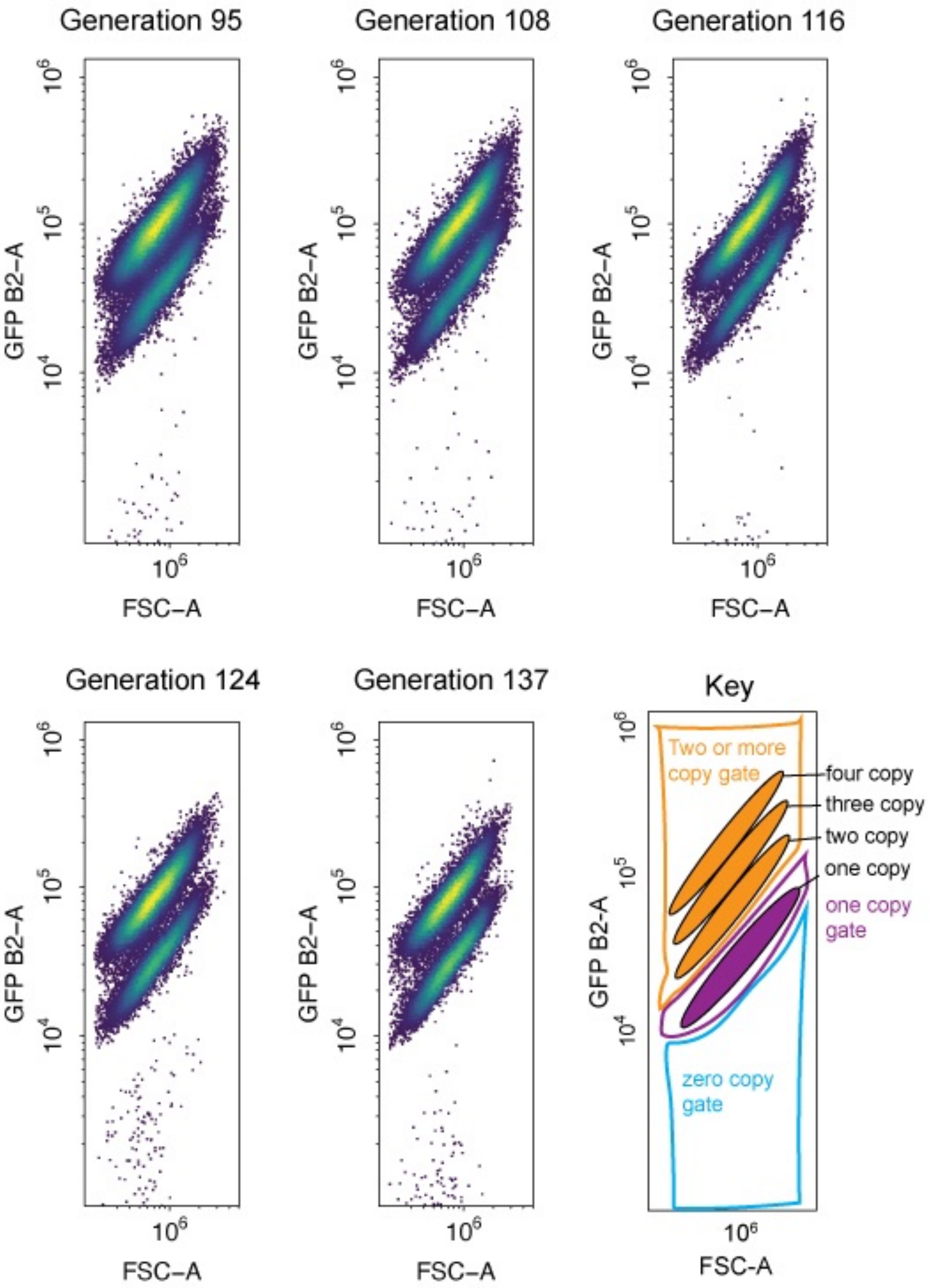

LTRΔ population 8

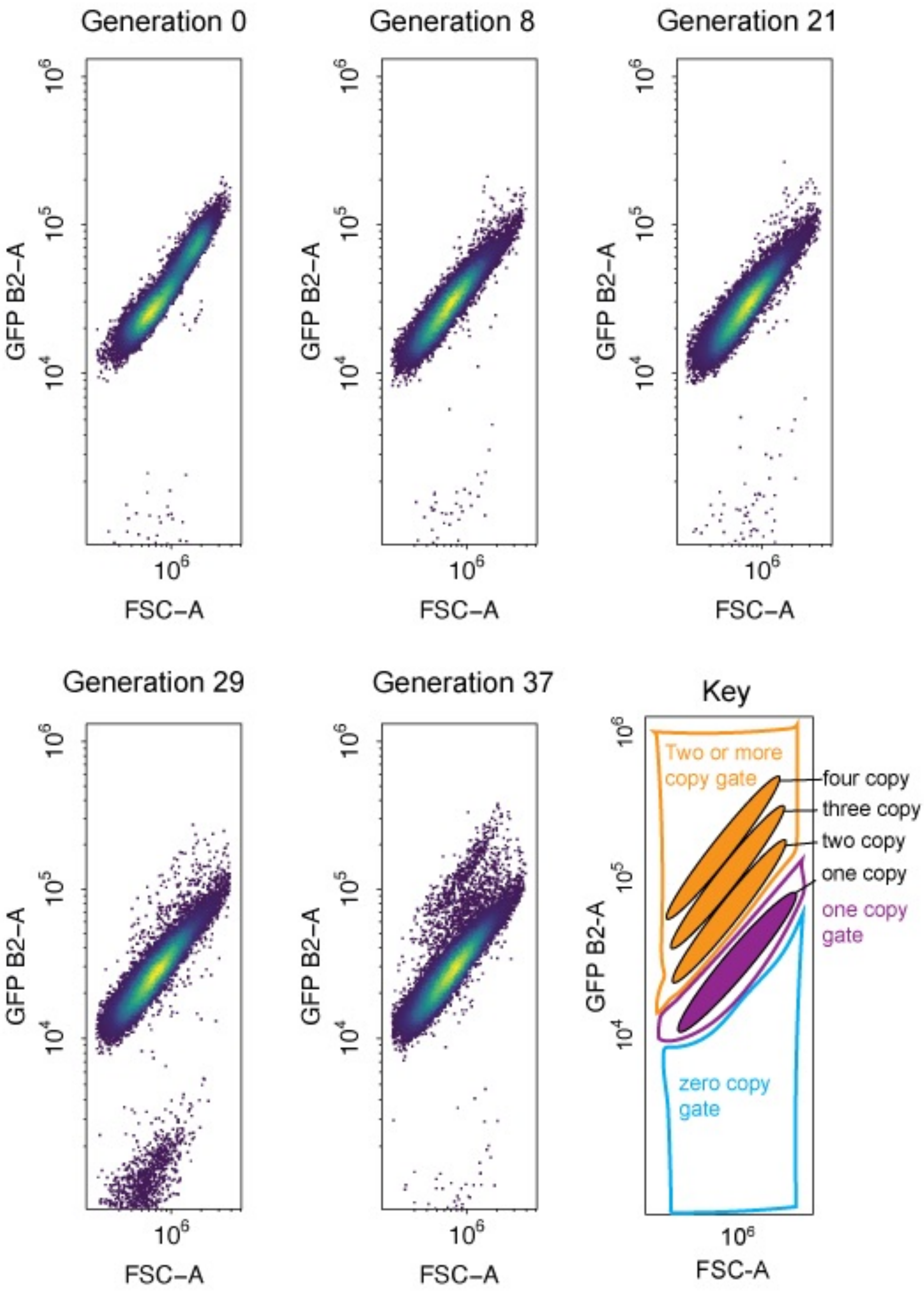

LTRΔ population 8

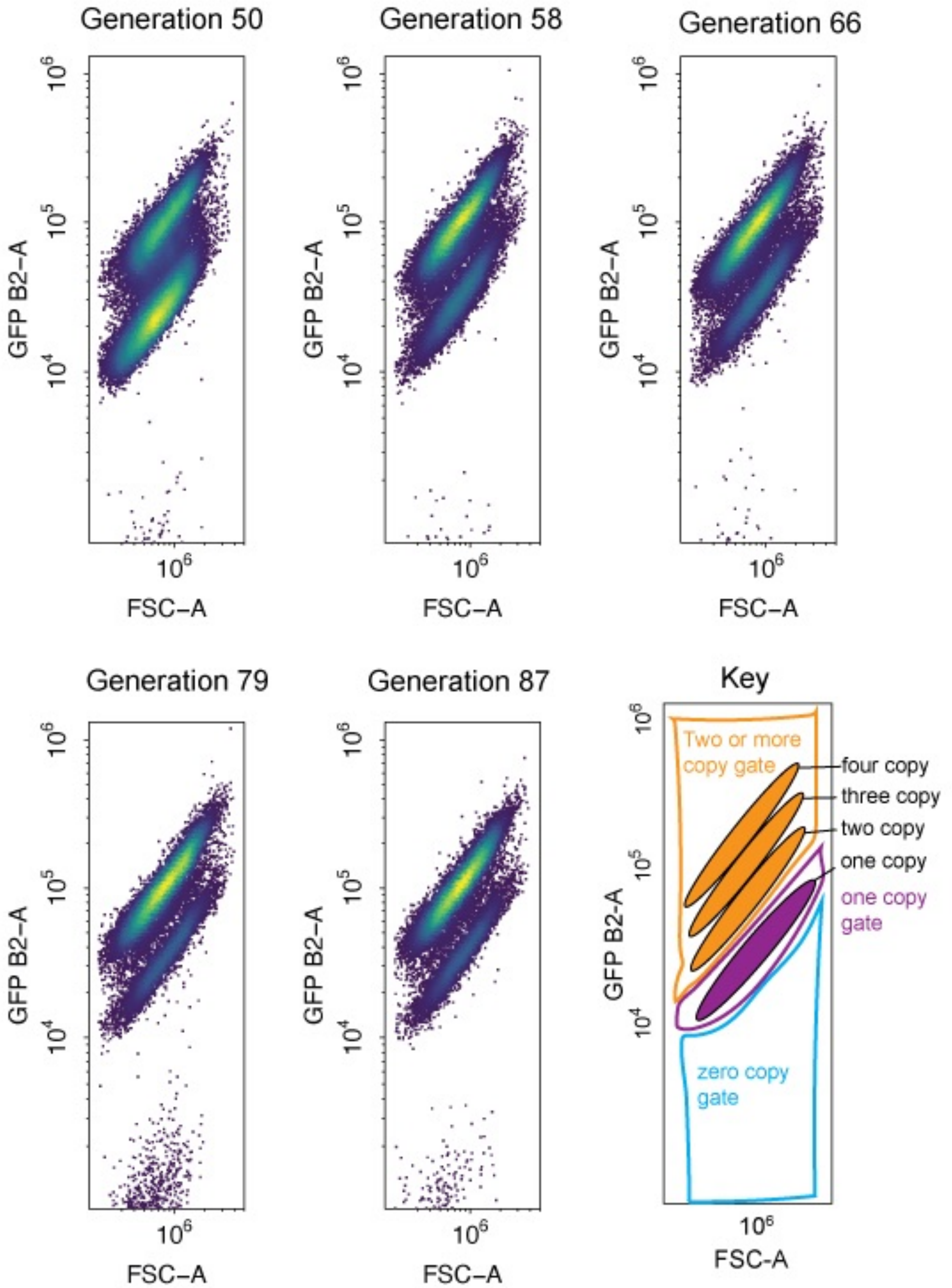

LTRΔ population 8

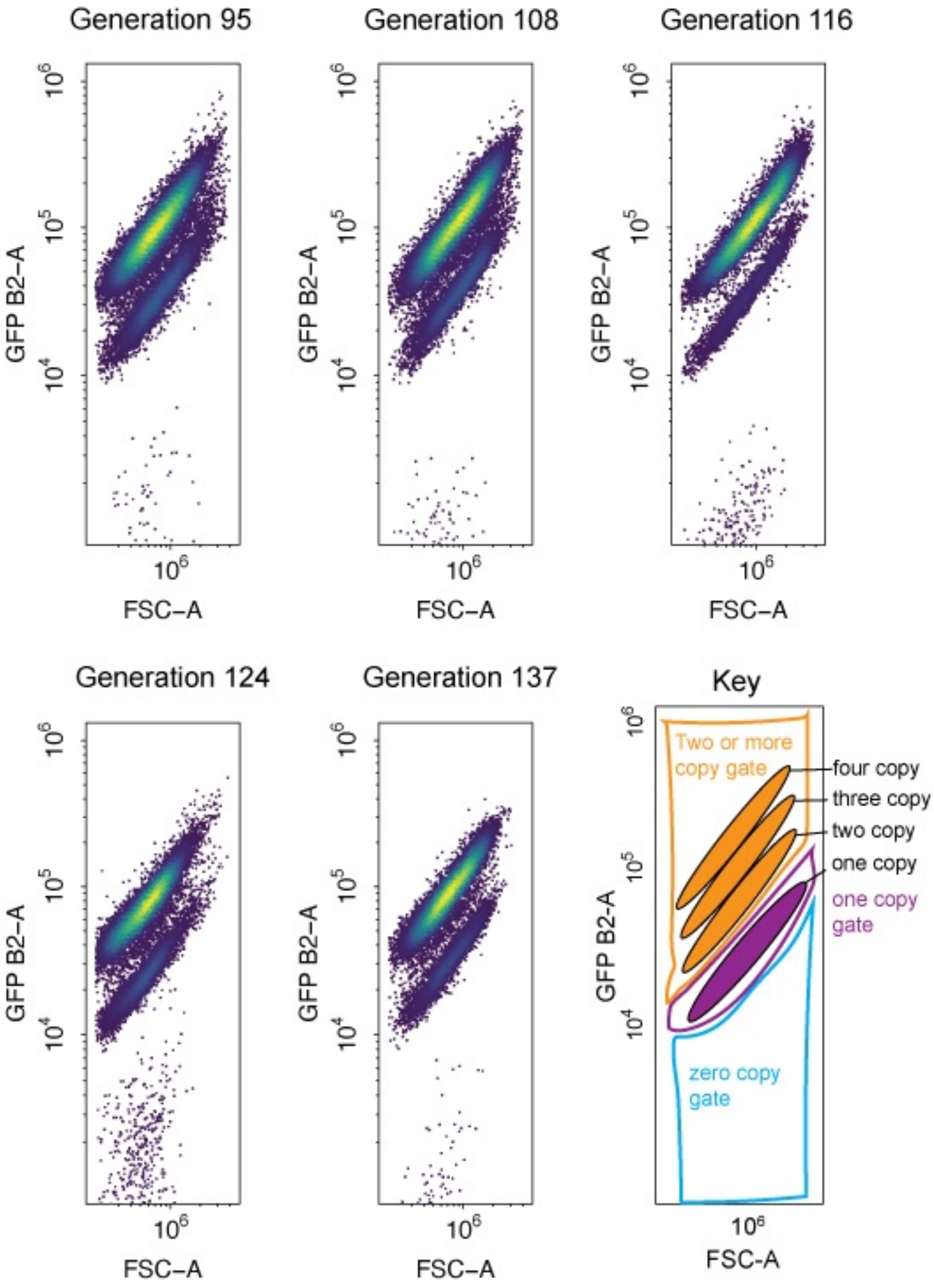

ARSΔ population 1

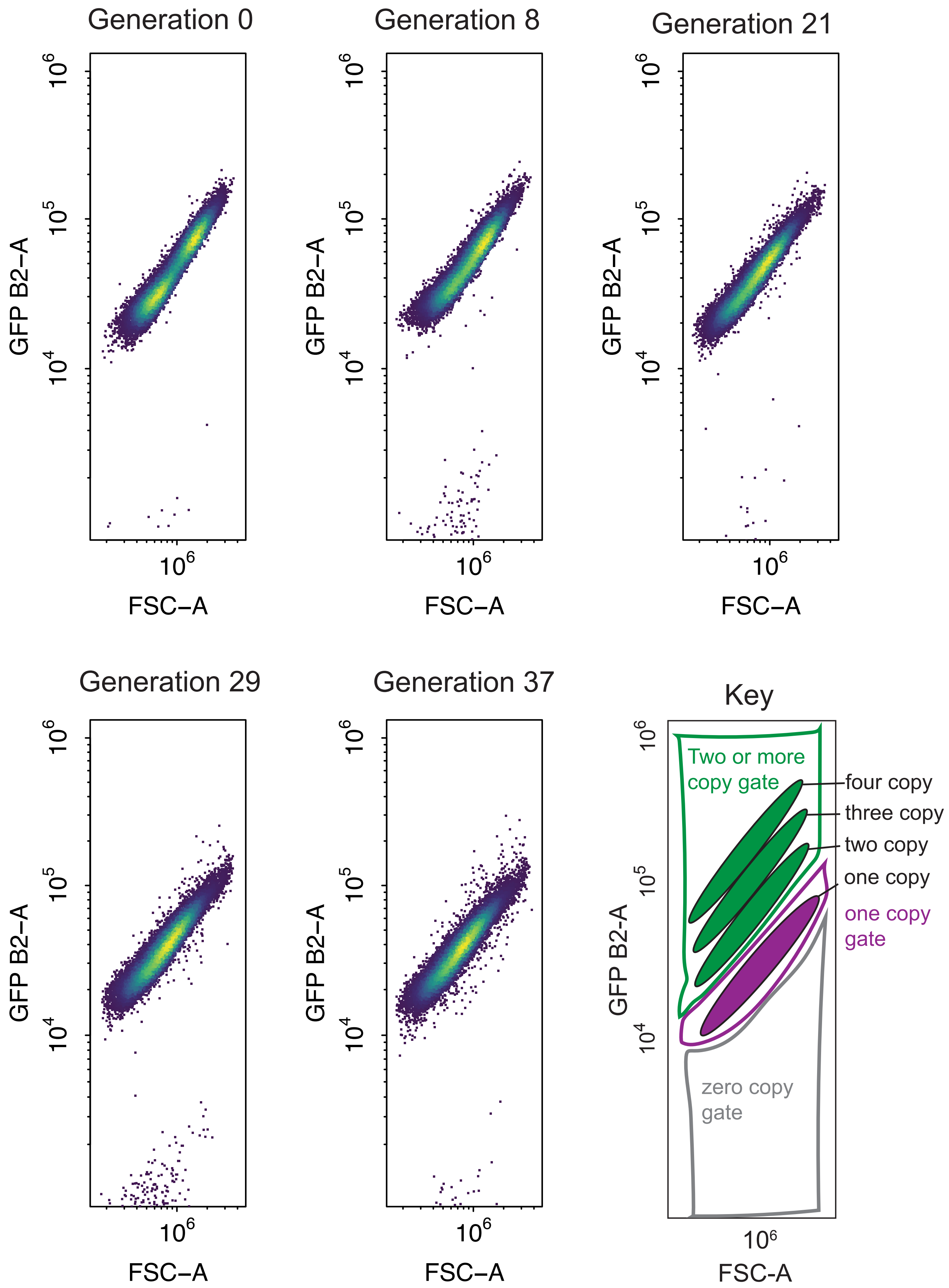

ARSΔ population 1

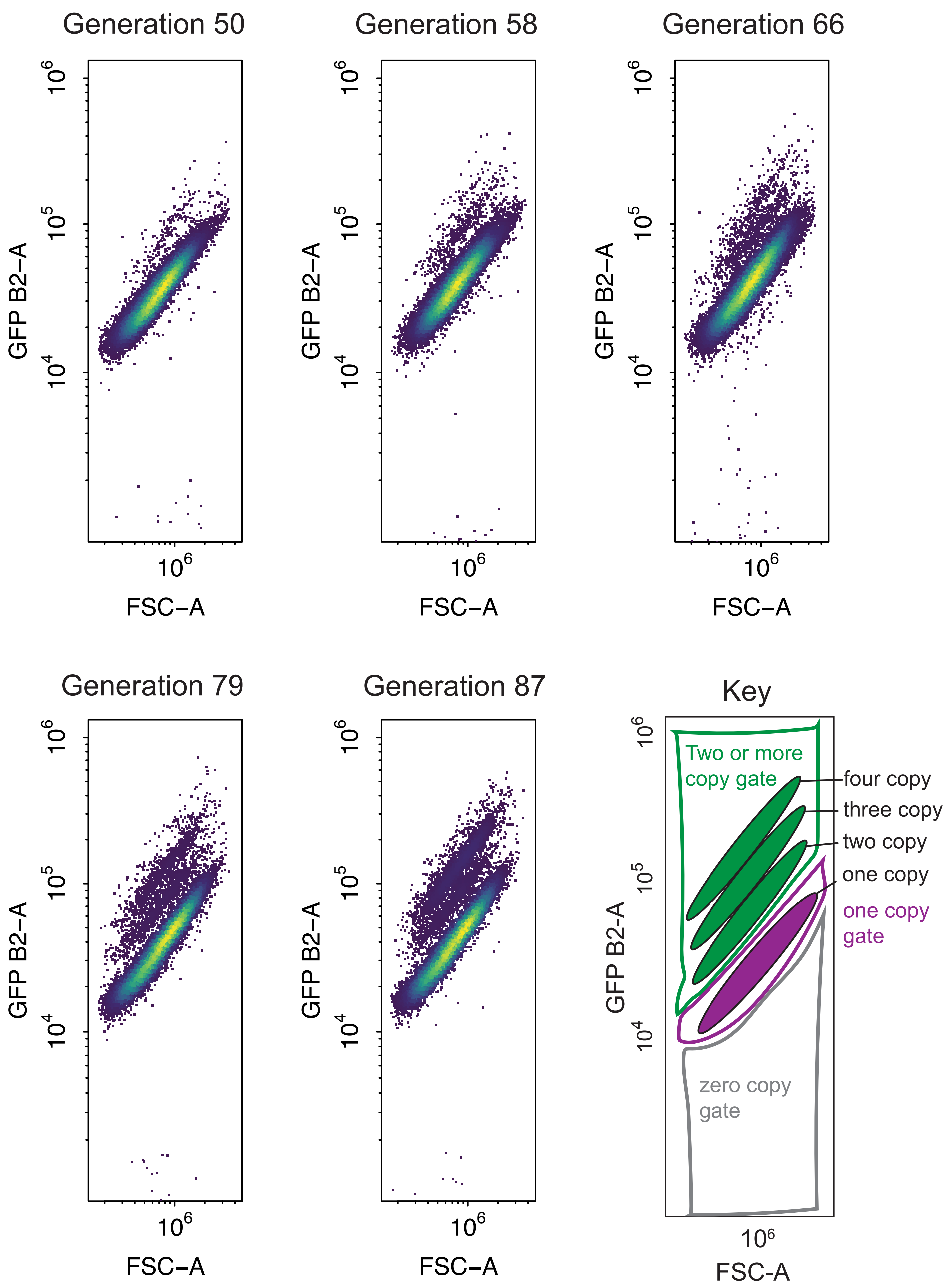

ARS∆ population 1

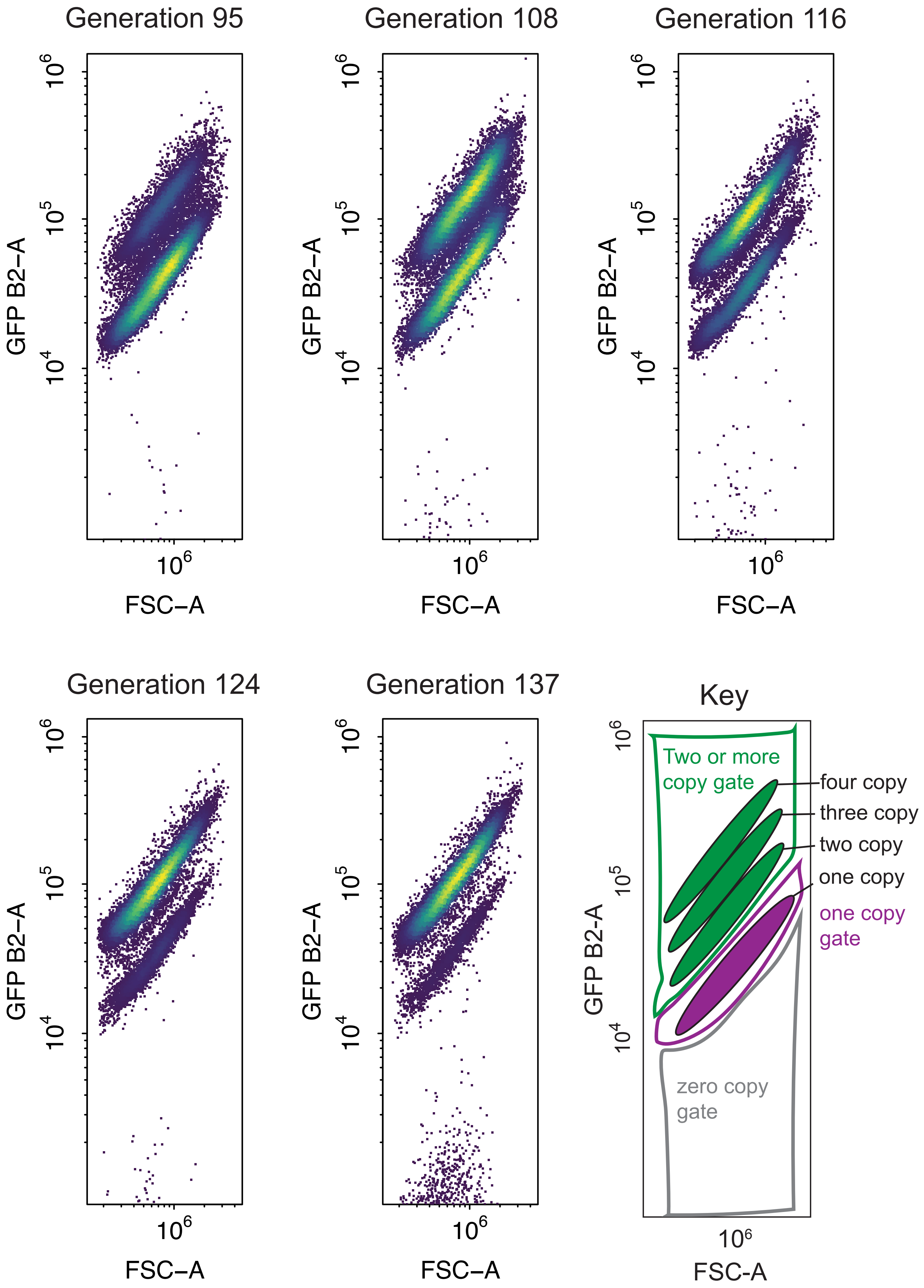

ARSΔ population 3

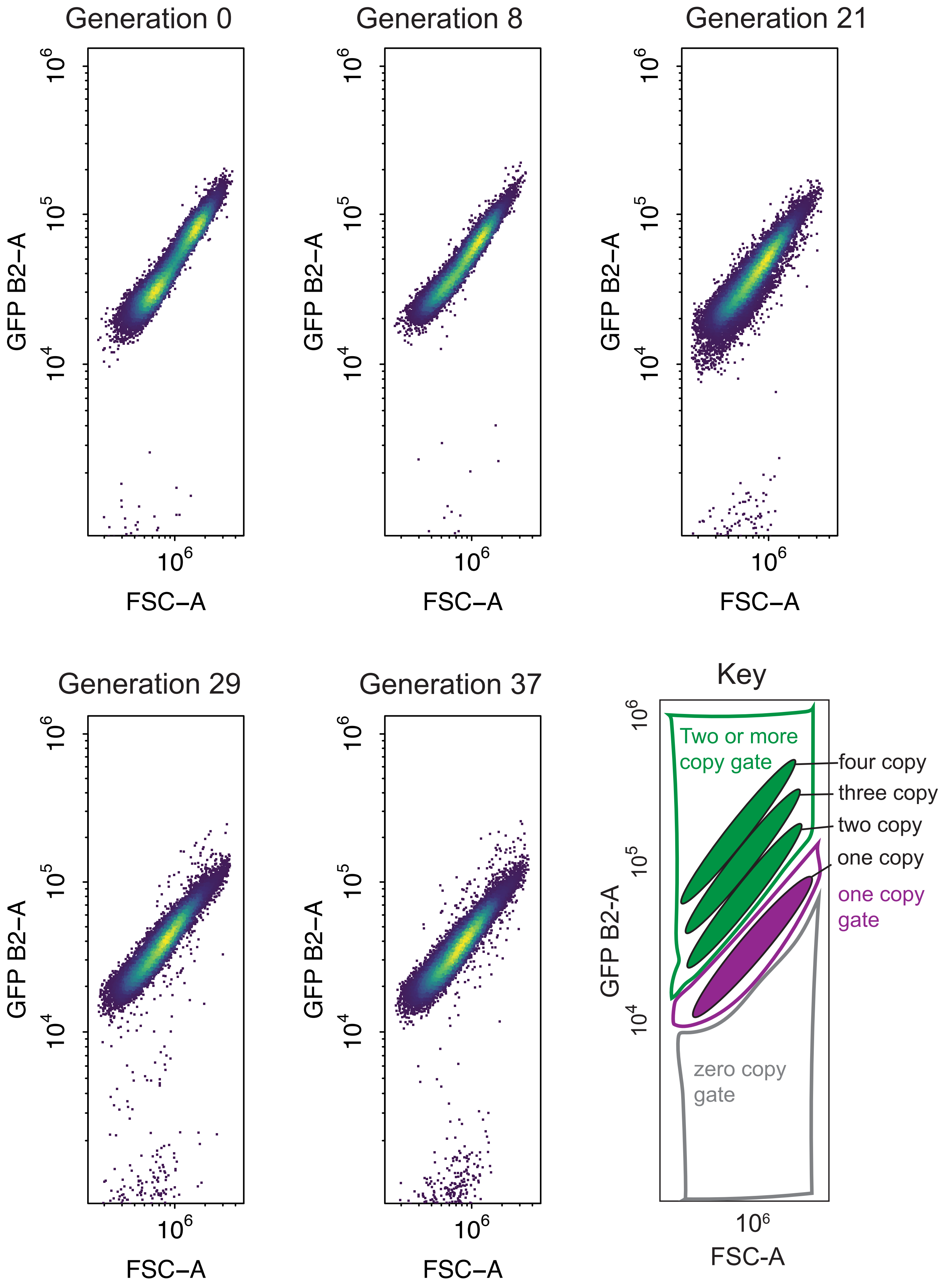

ARSΔ population 3

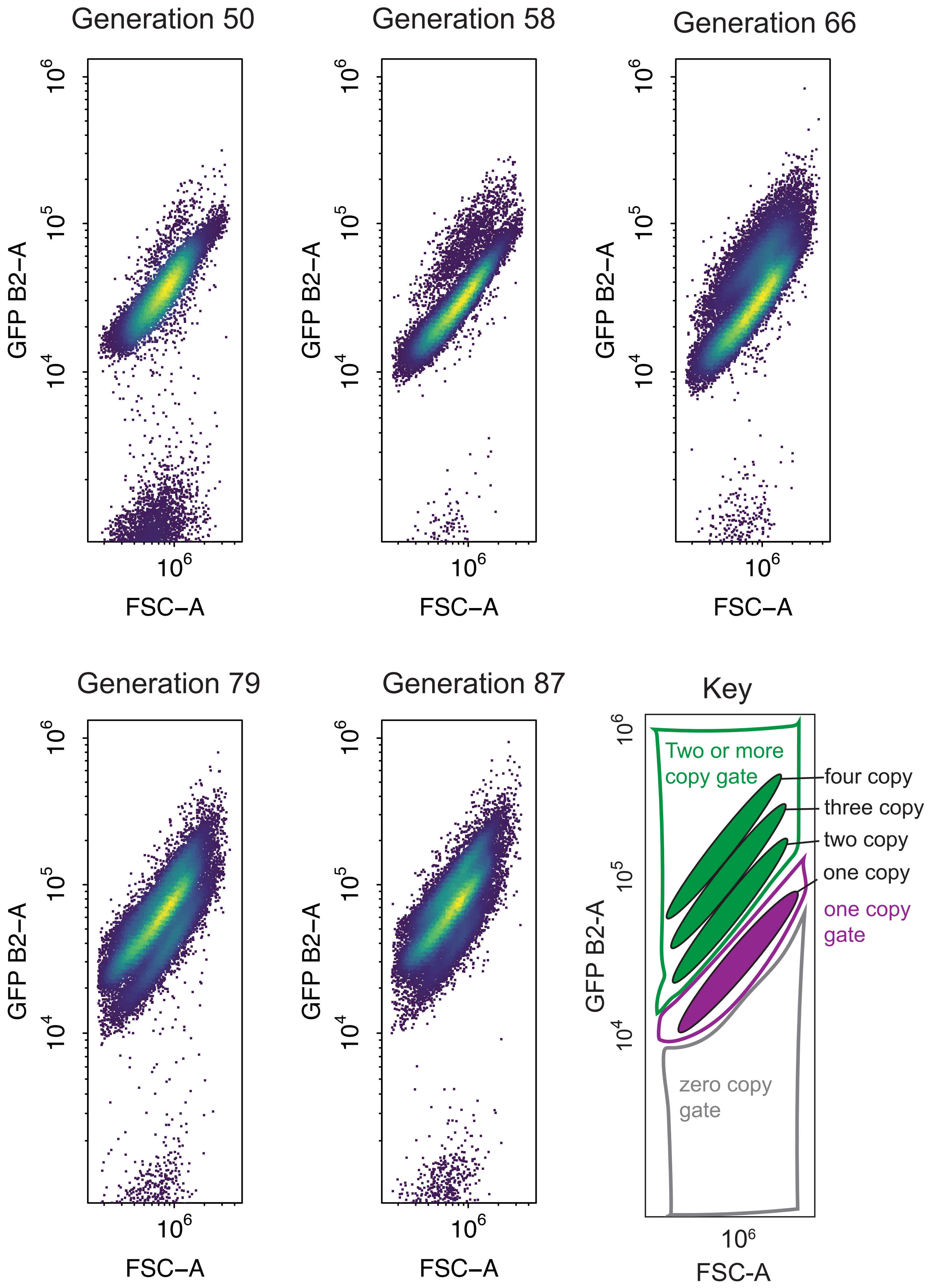

ARSΔ population 3

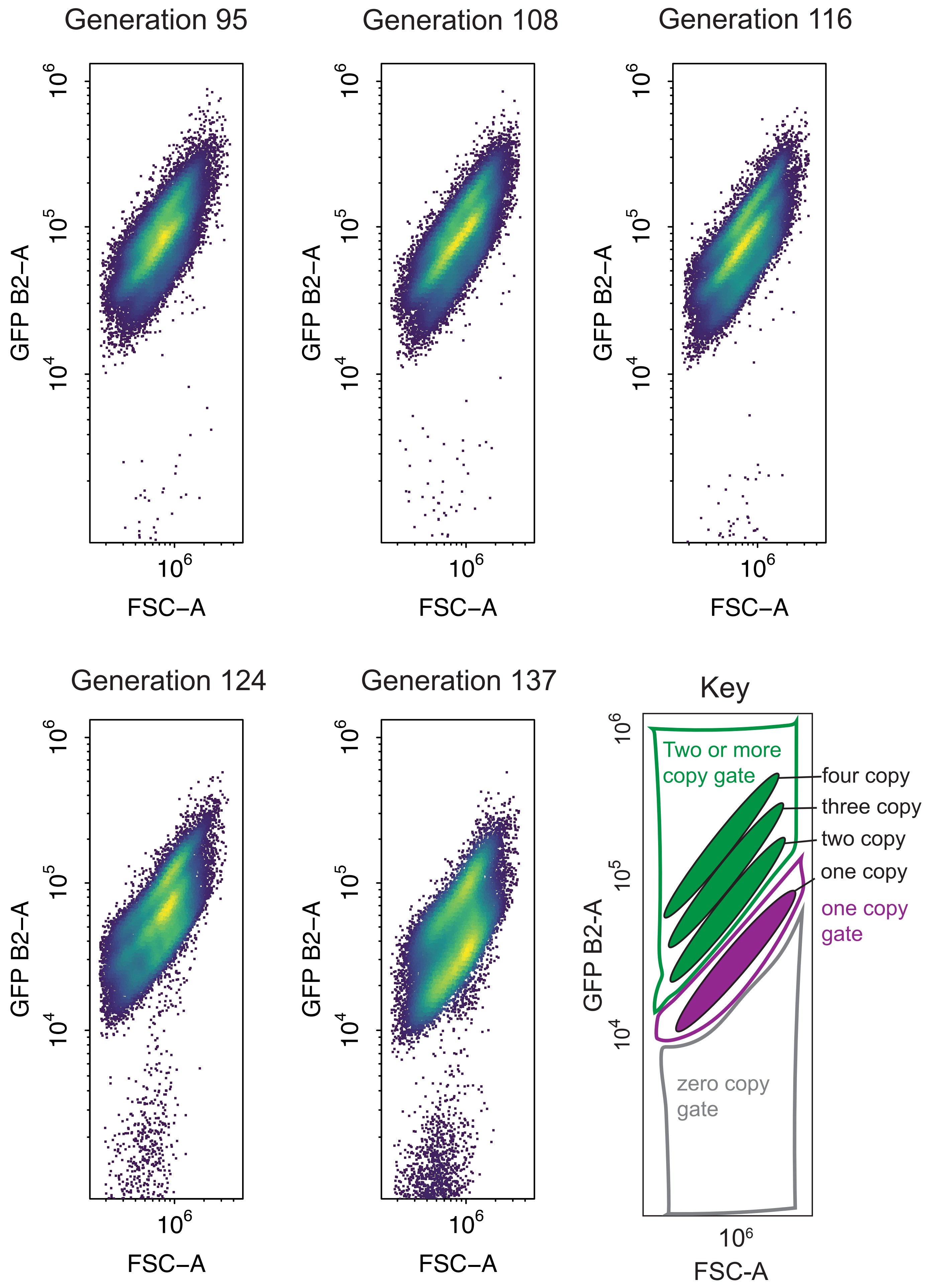

ARSΔ population 4

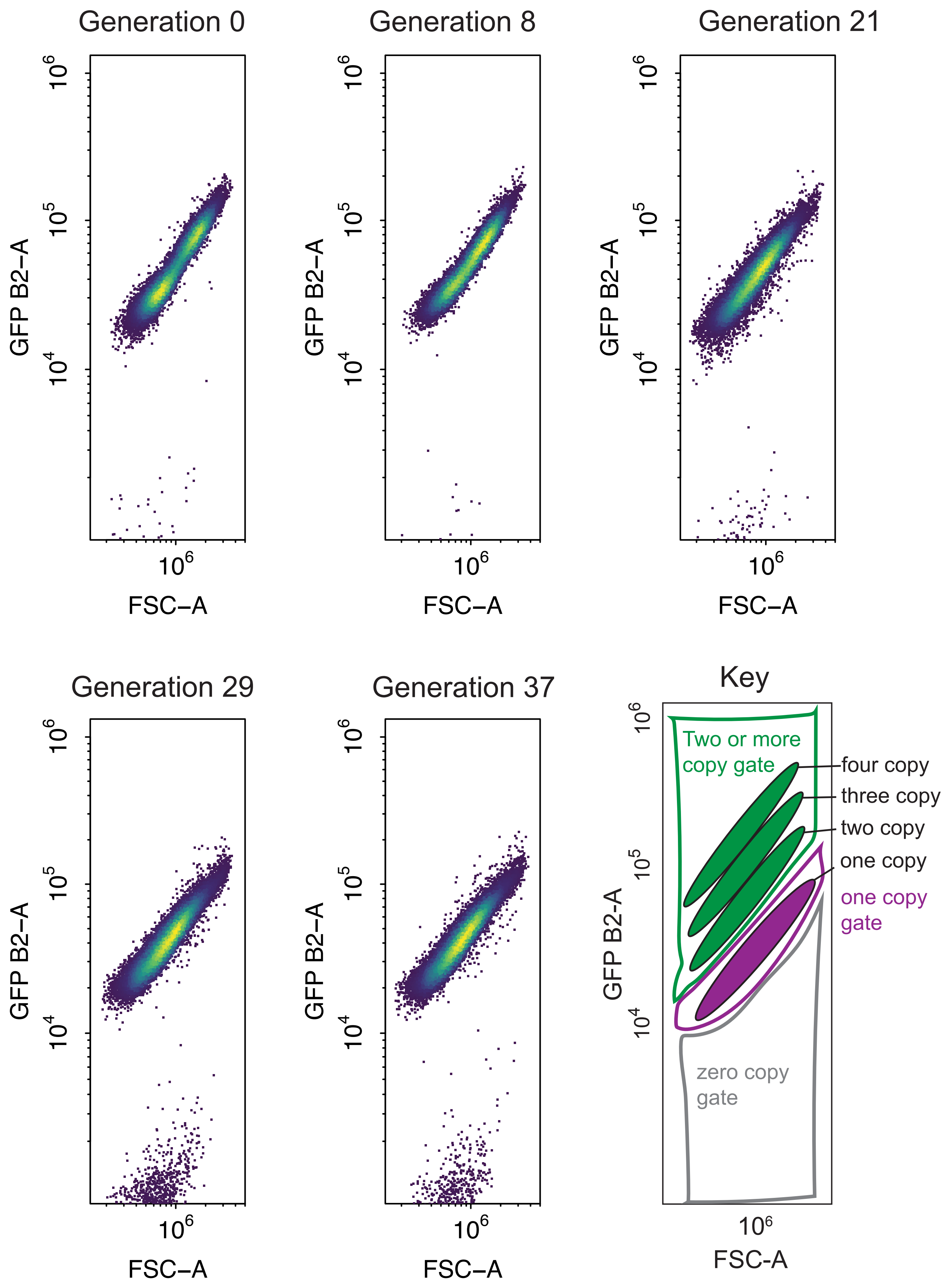

ARSΔ population 4

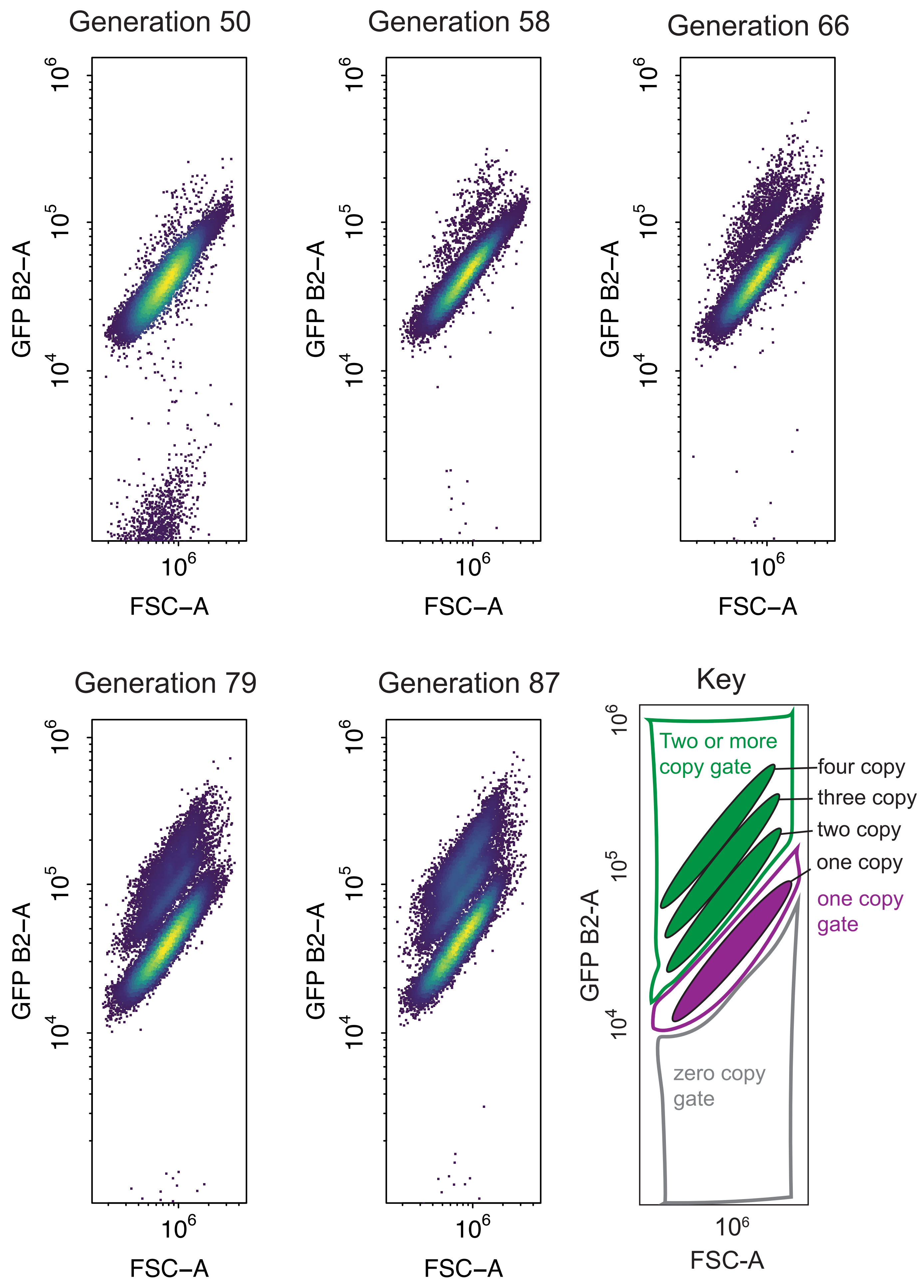

ARSΔ population 4

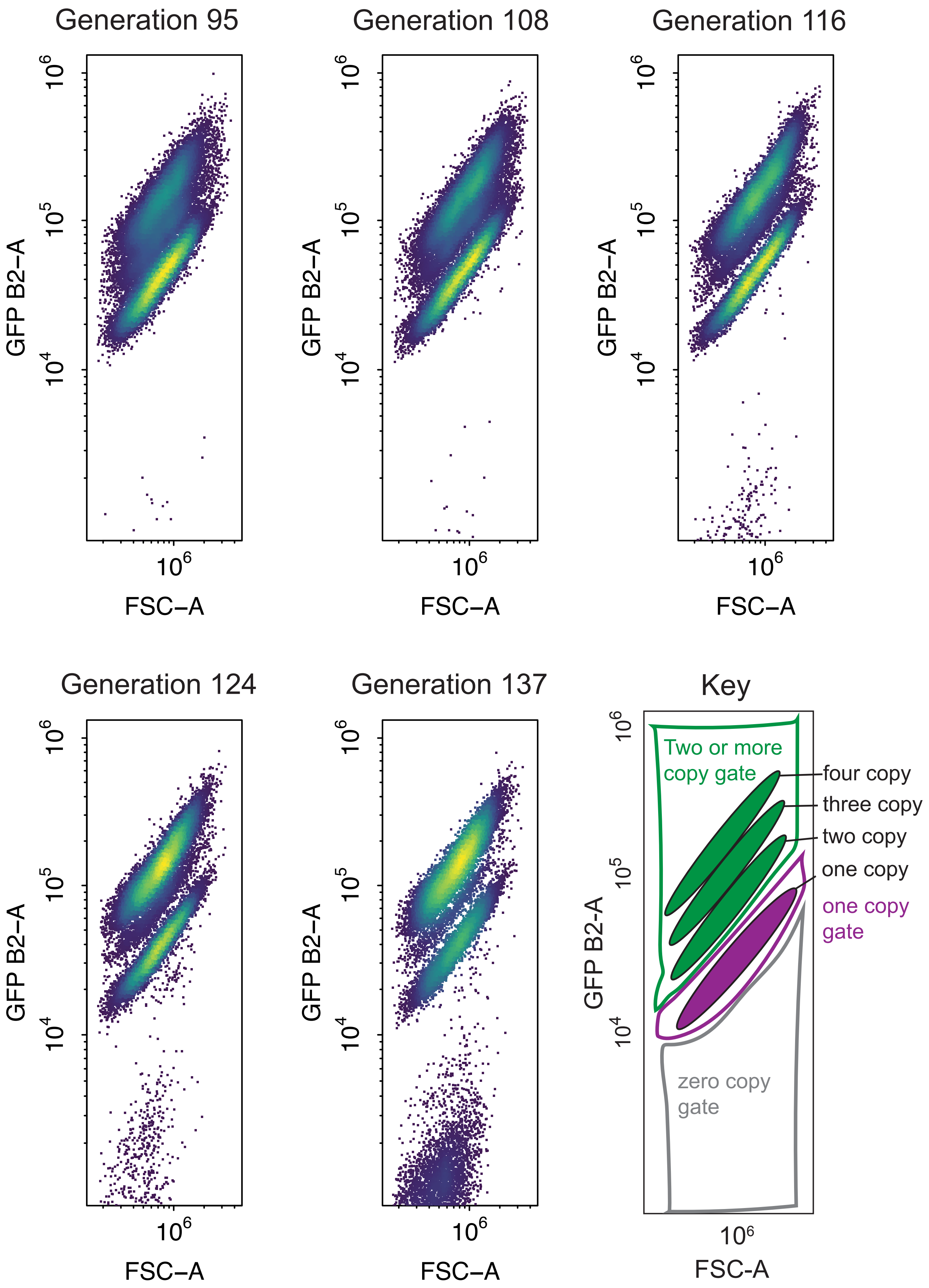

ARSΔ population 5

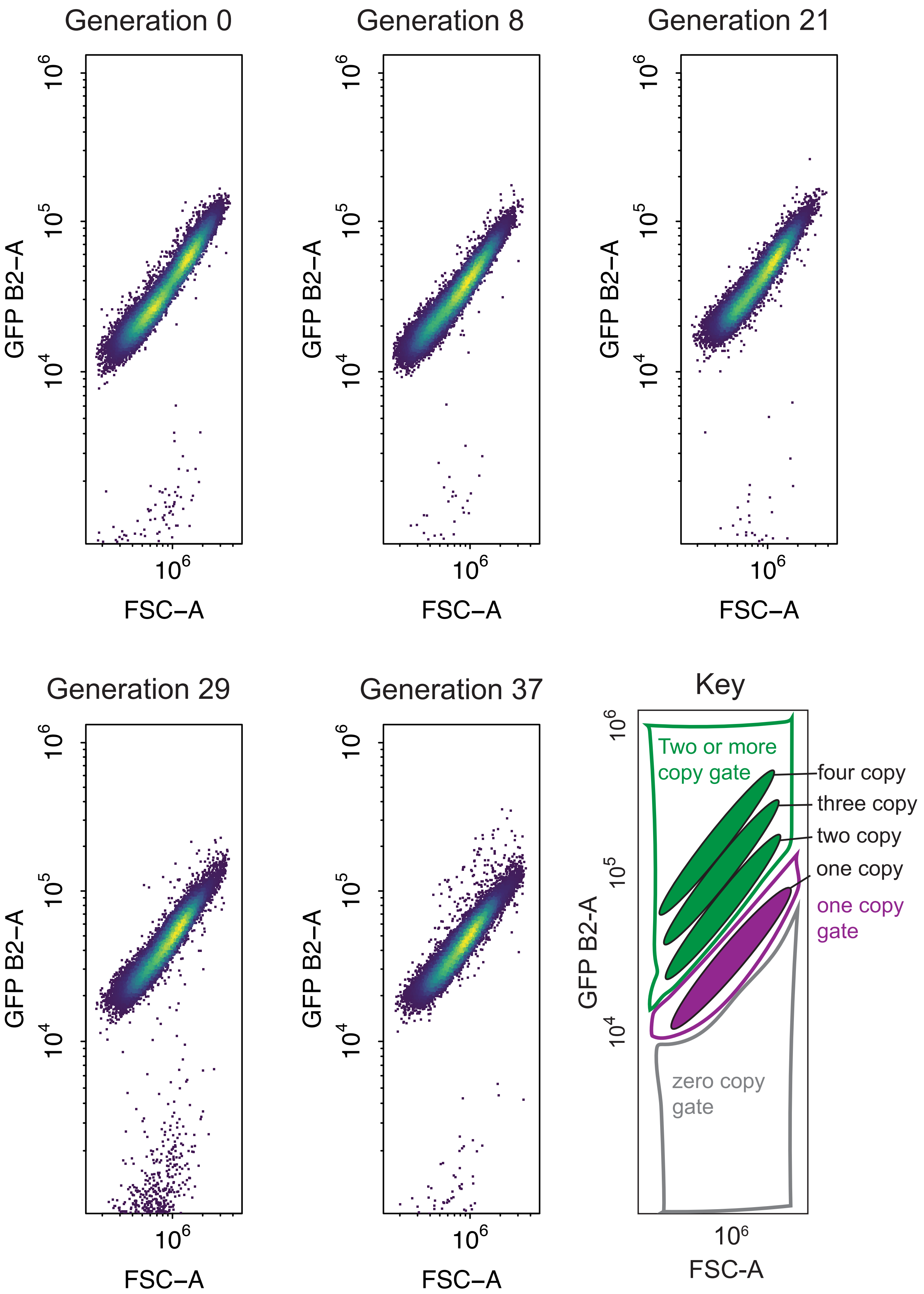

ARSΔ population 5

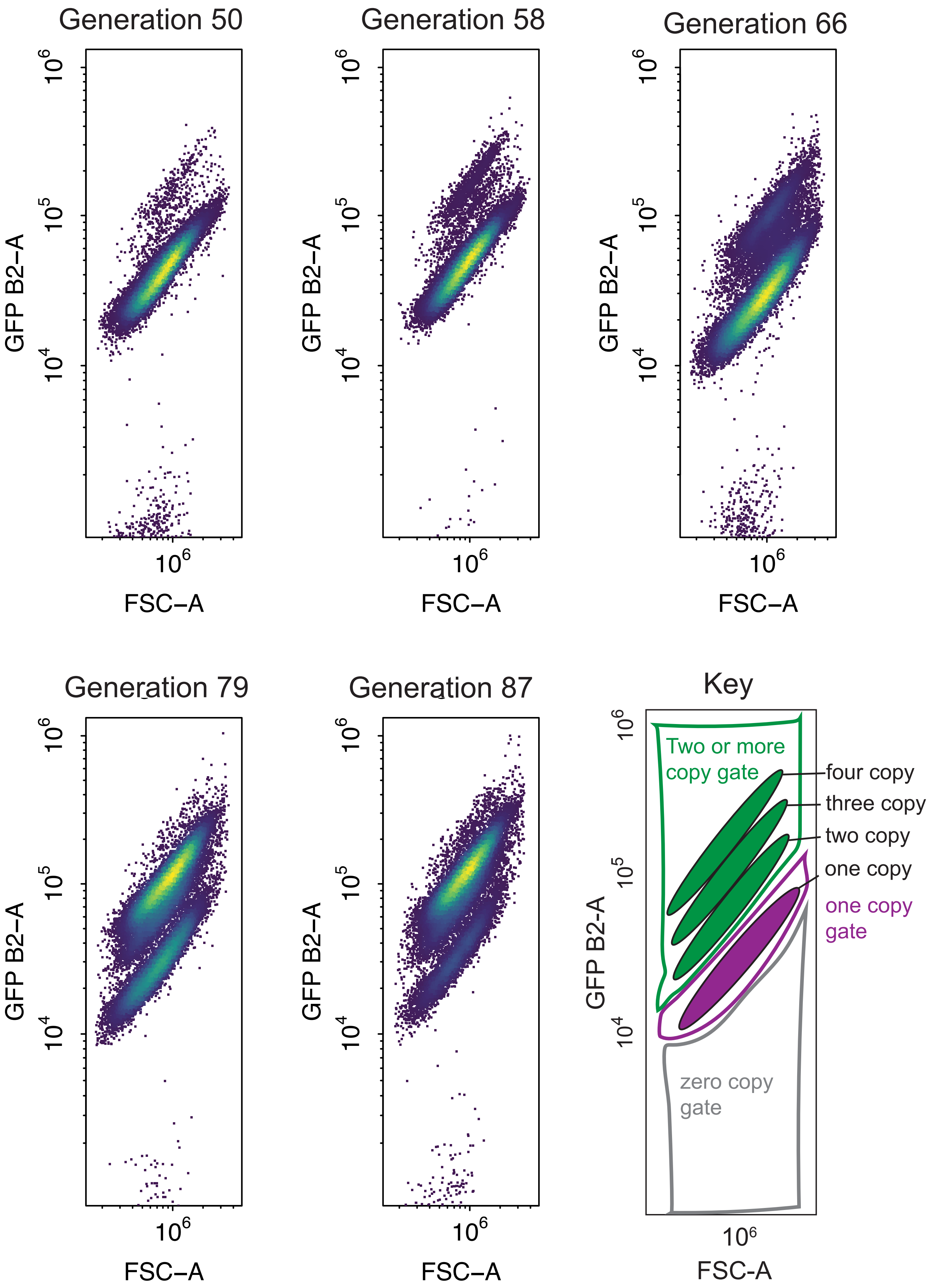

ARSΔ population 5

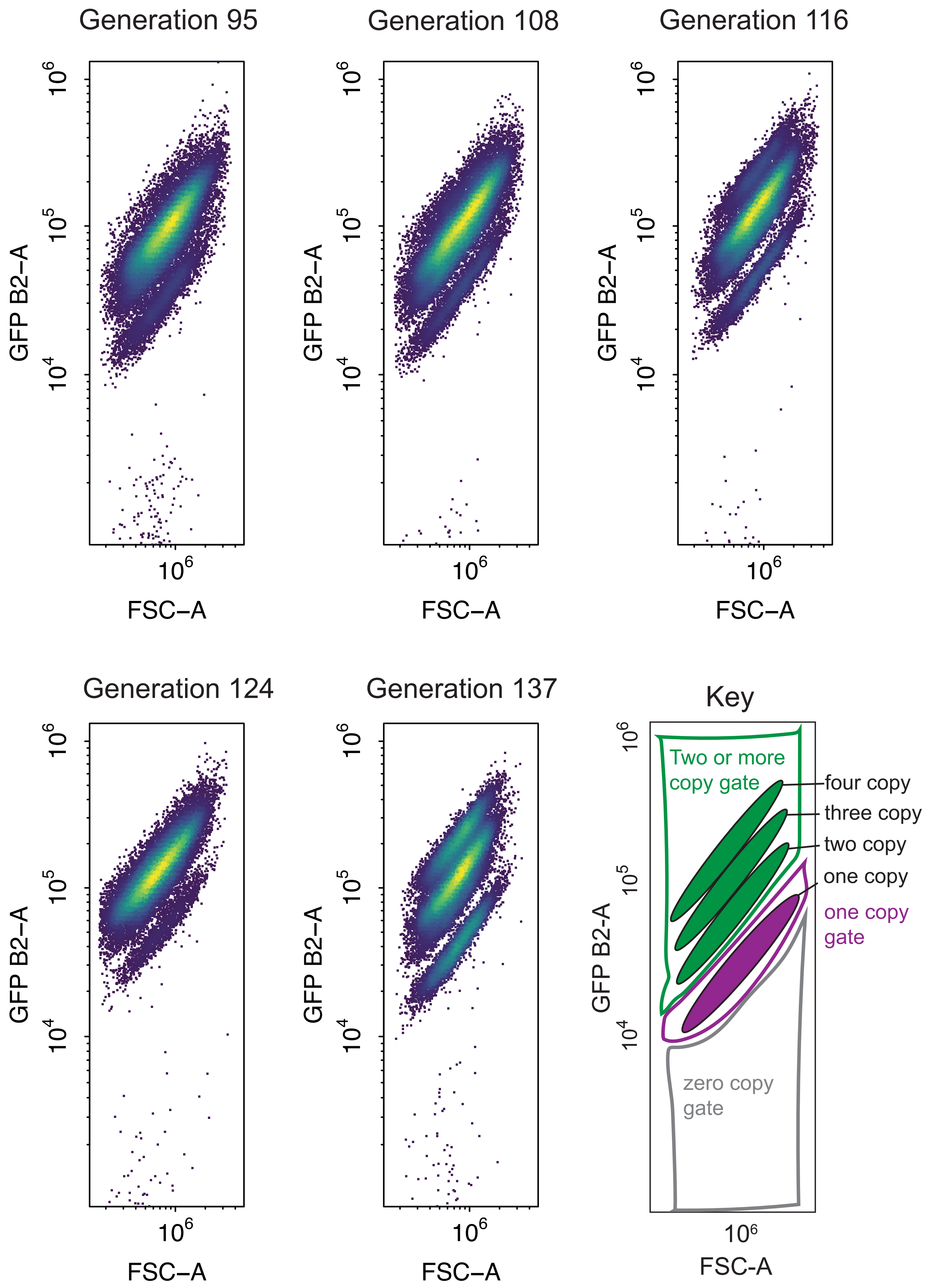

ARSΔ population 6

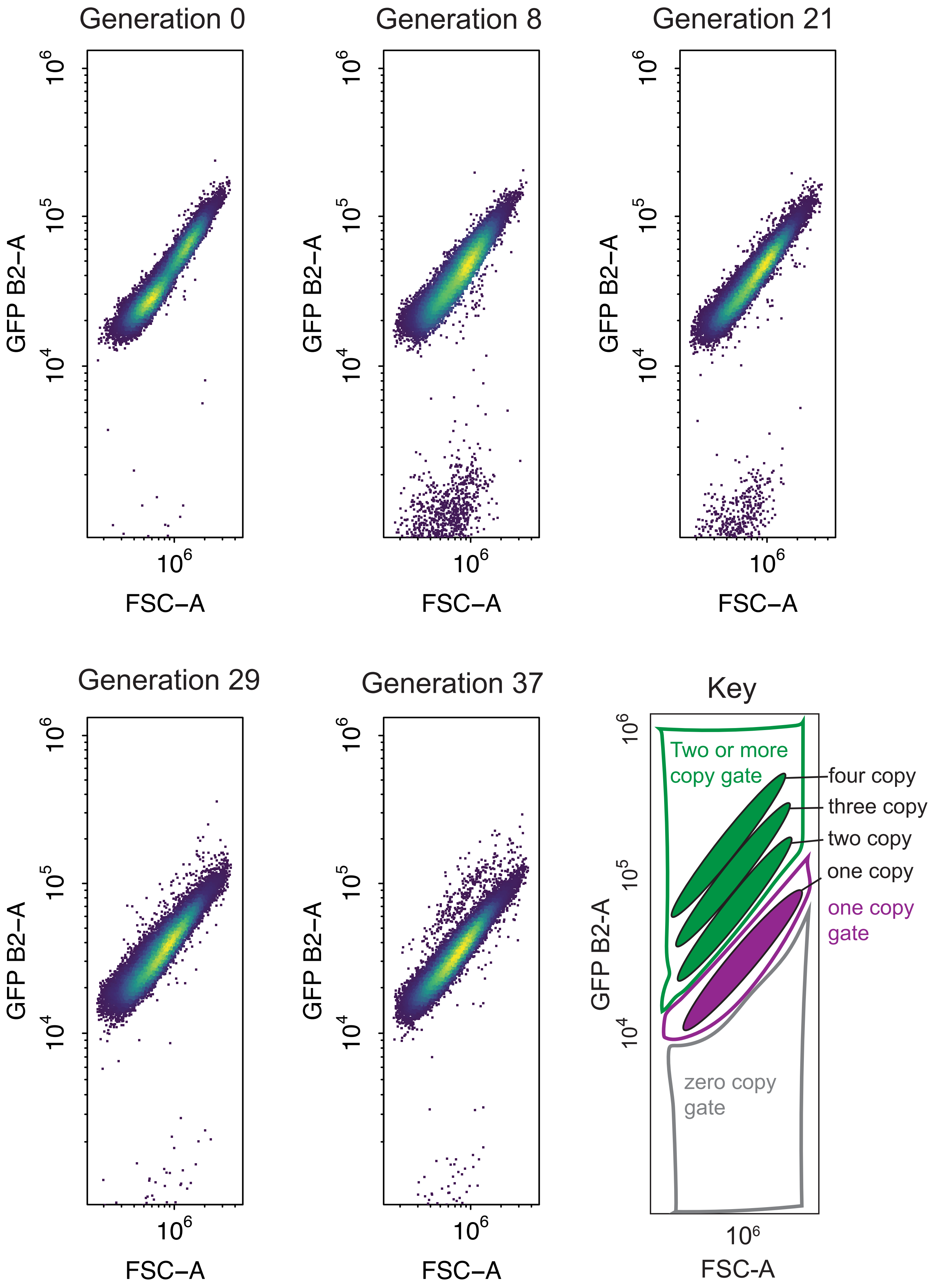

ARSΔ population 6

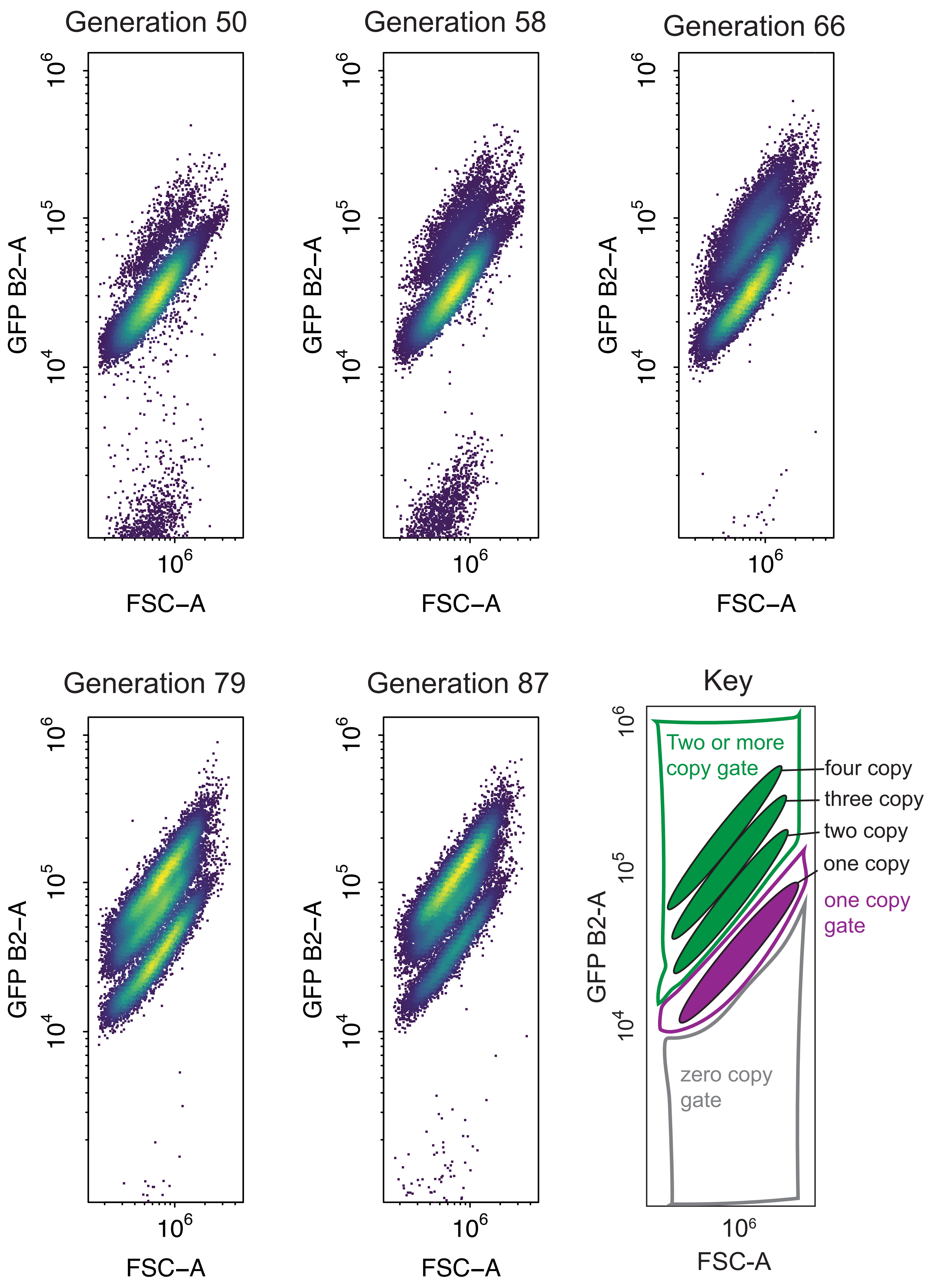

ARSΔ population 6

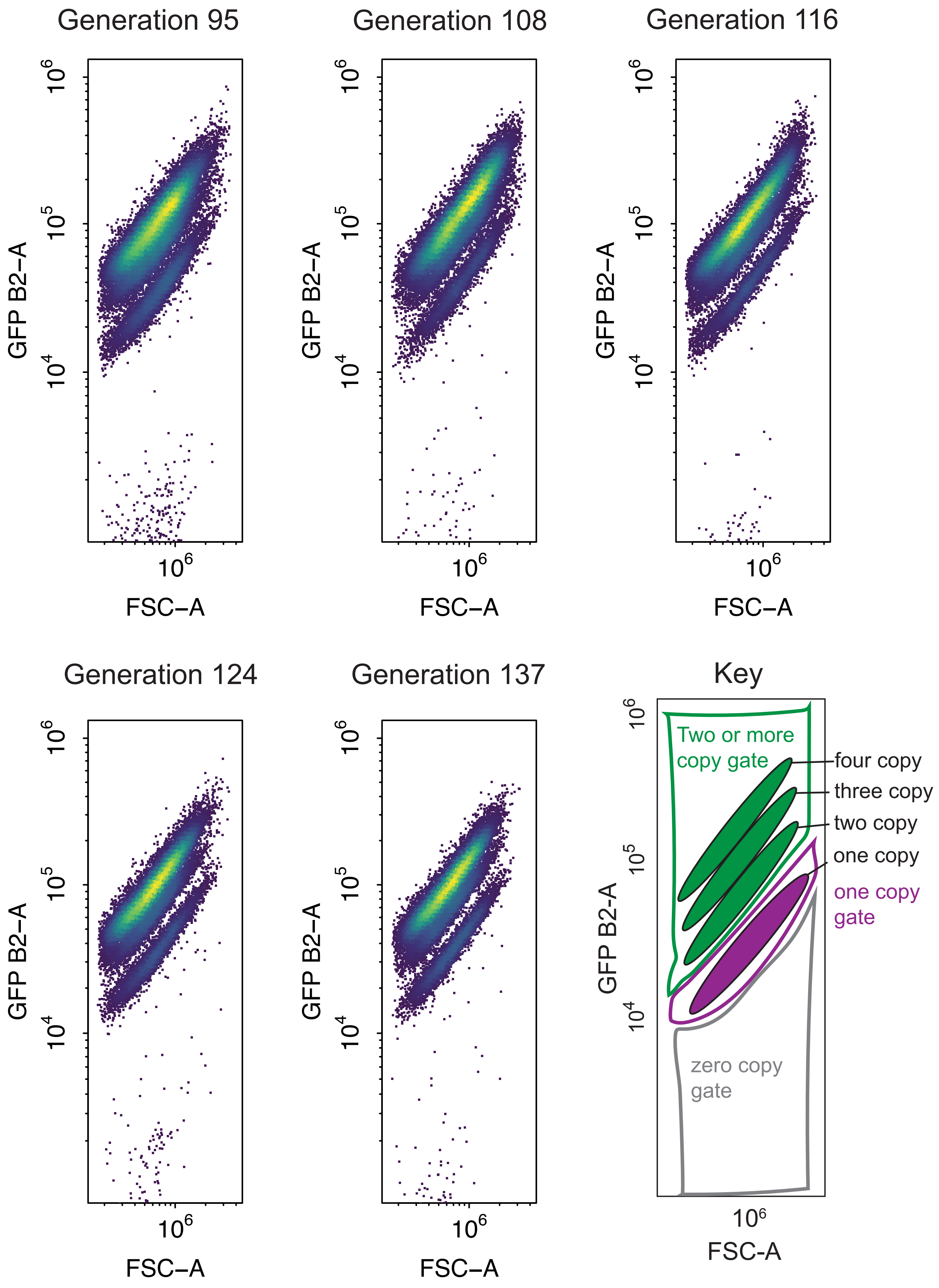

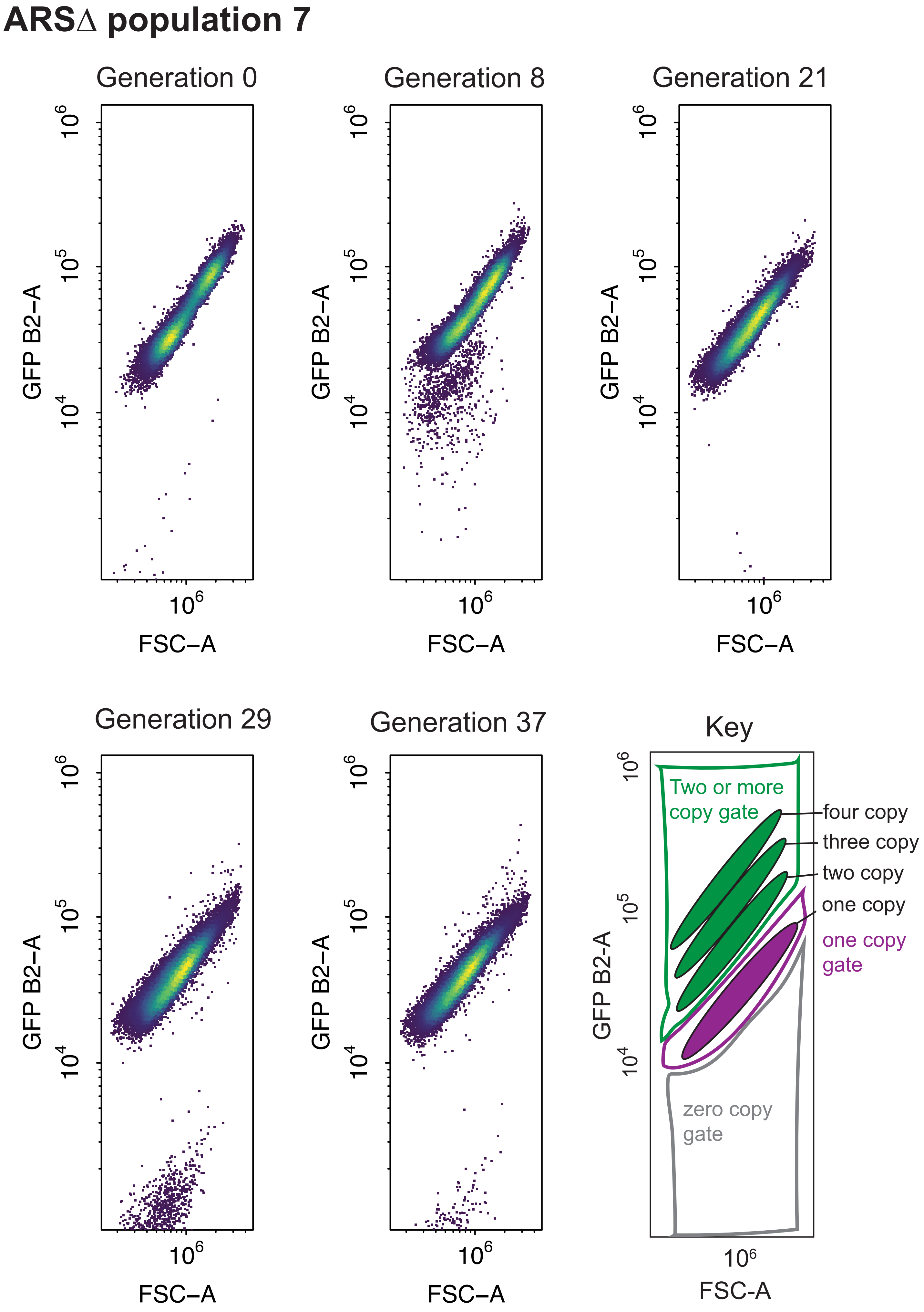
ARSΔ population 7
Generation 0
Generation 8
Generation 21
Generation 29
Generation 37
Key
GFP B2–A
FSC–A
10^4
10^5
10^6
Two or more copy gate
four copy
three copy
two copy
one copy
one copy gate
zero copy gate

ARSΔ population 7

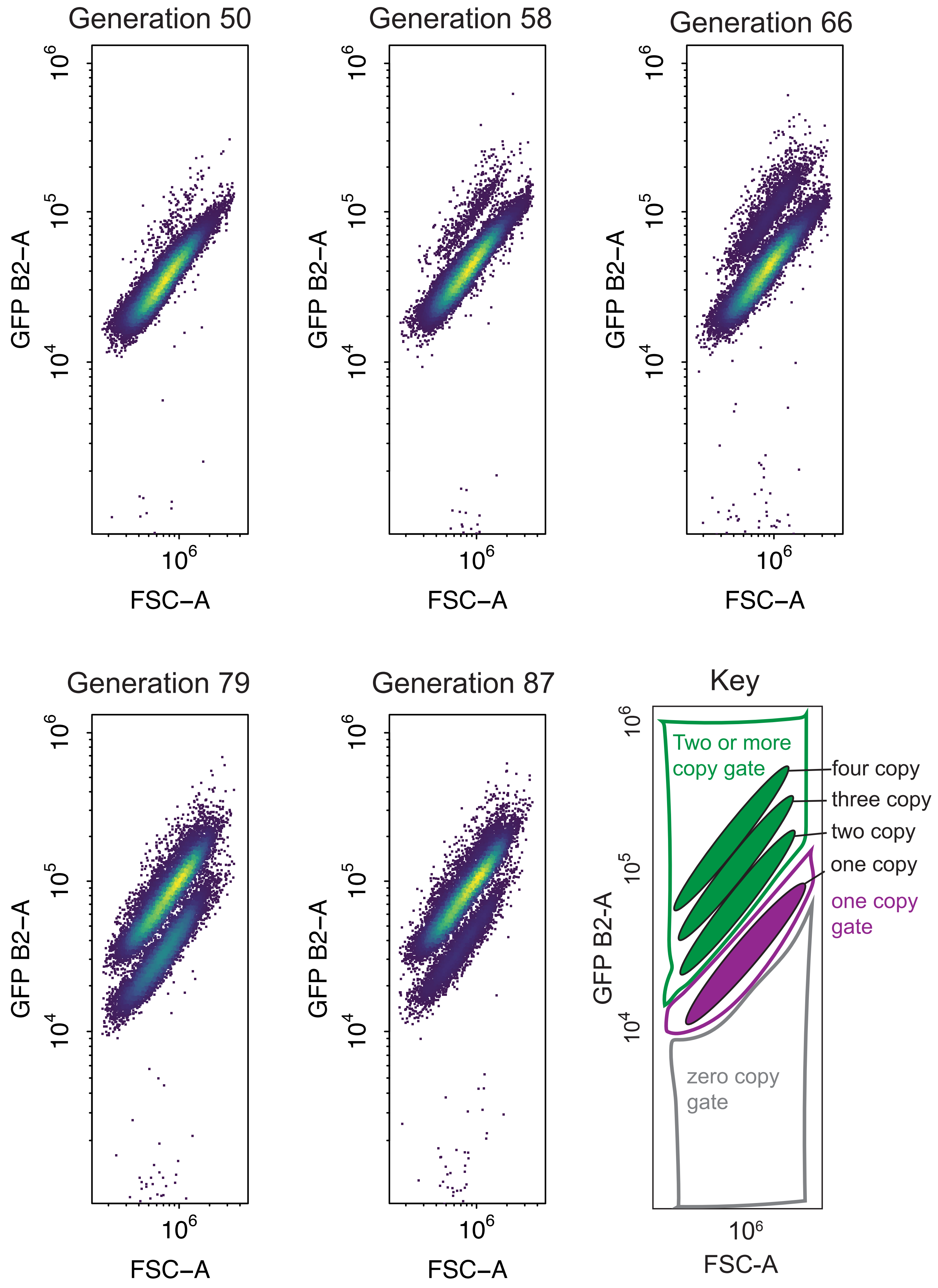

ARSΔ population 7

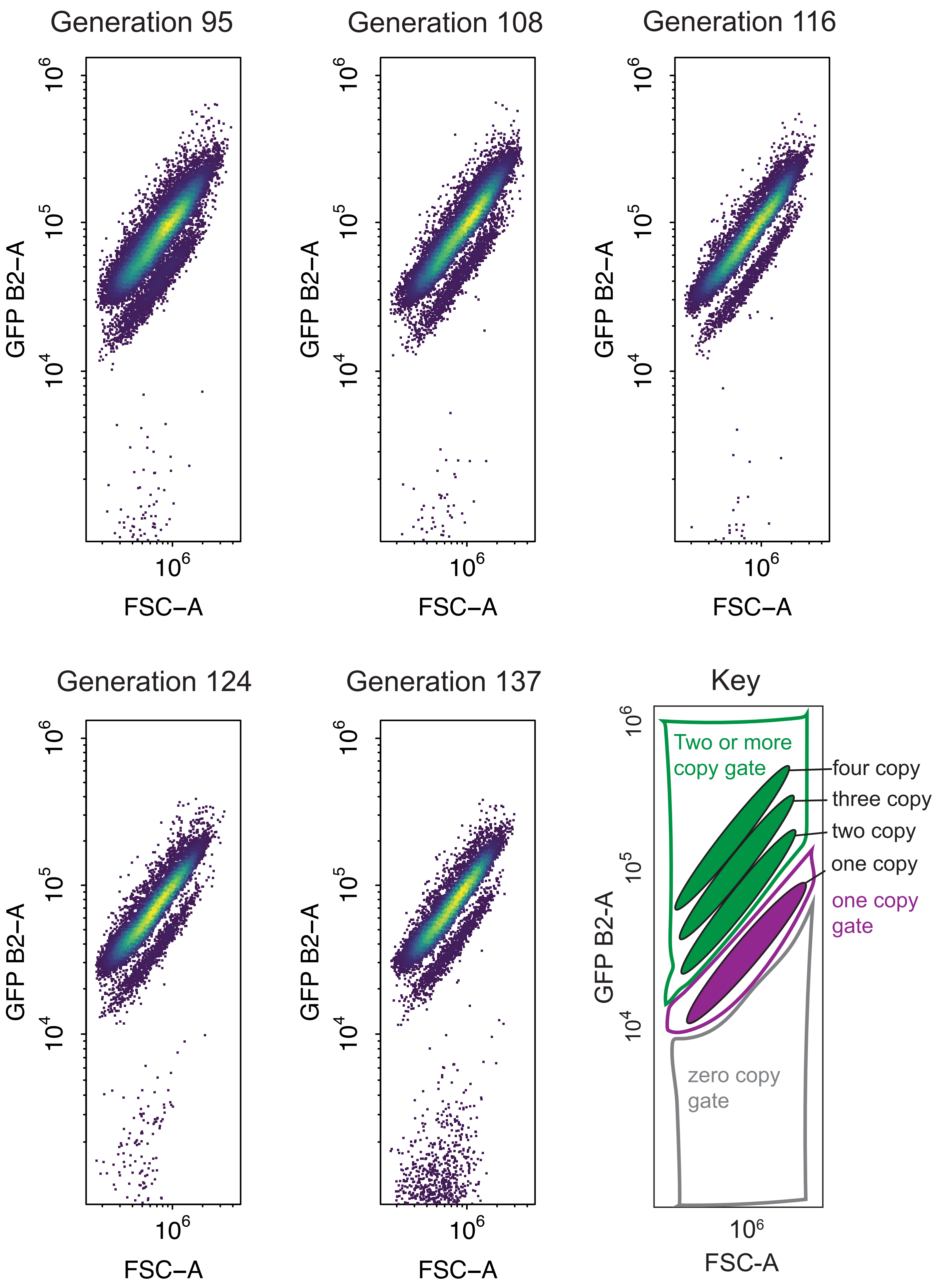

ARSΔ population 8

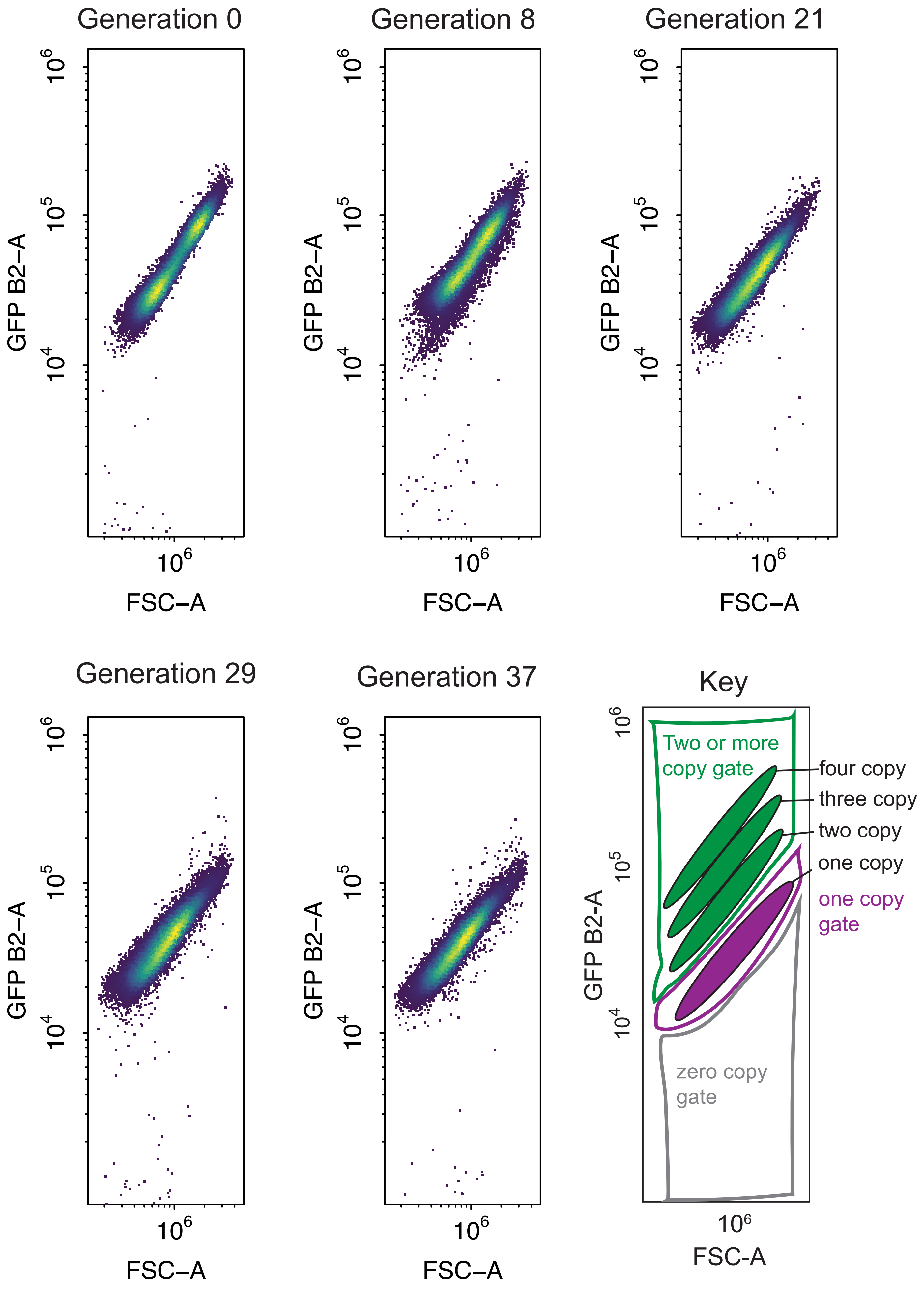

ARSΔ population 8

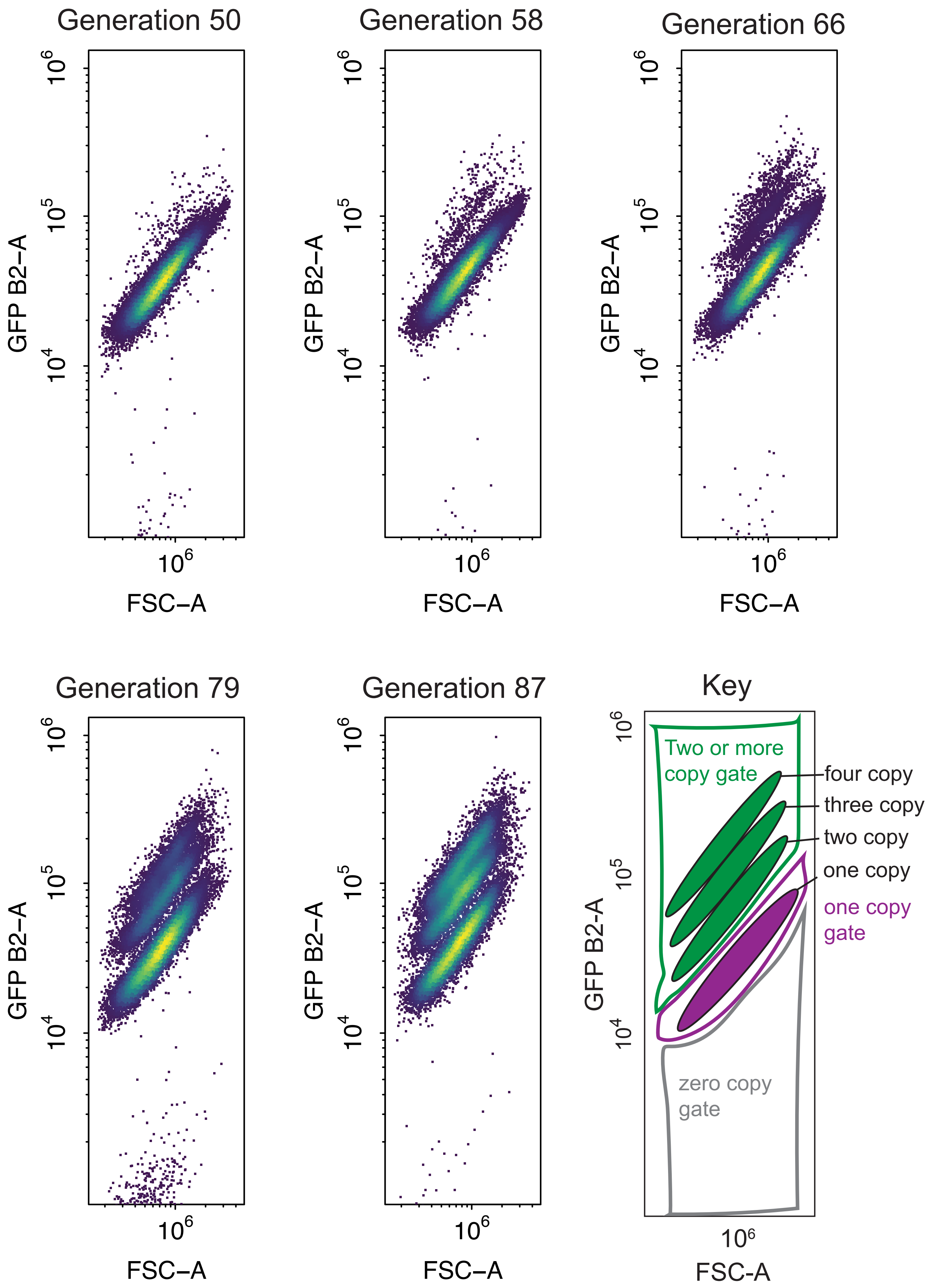

ARS∆ population 8

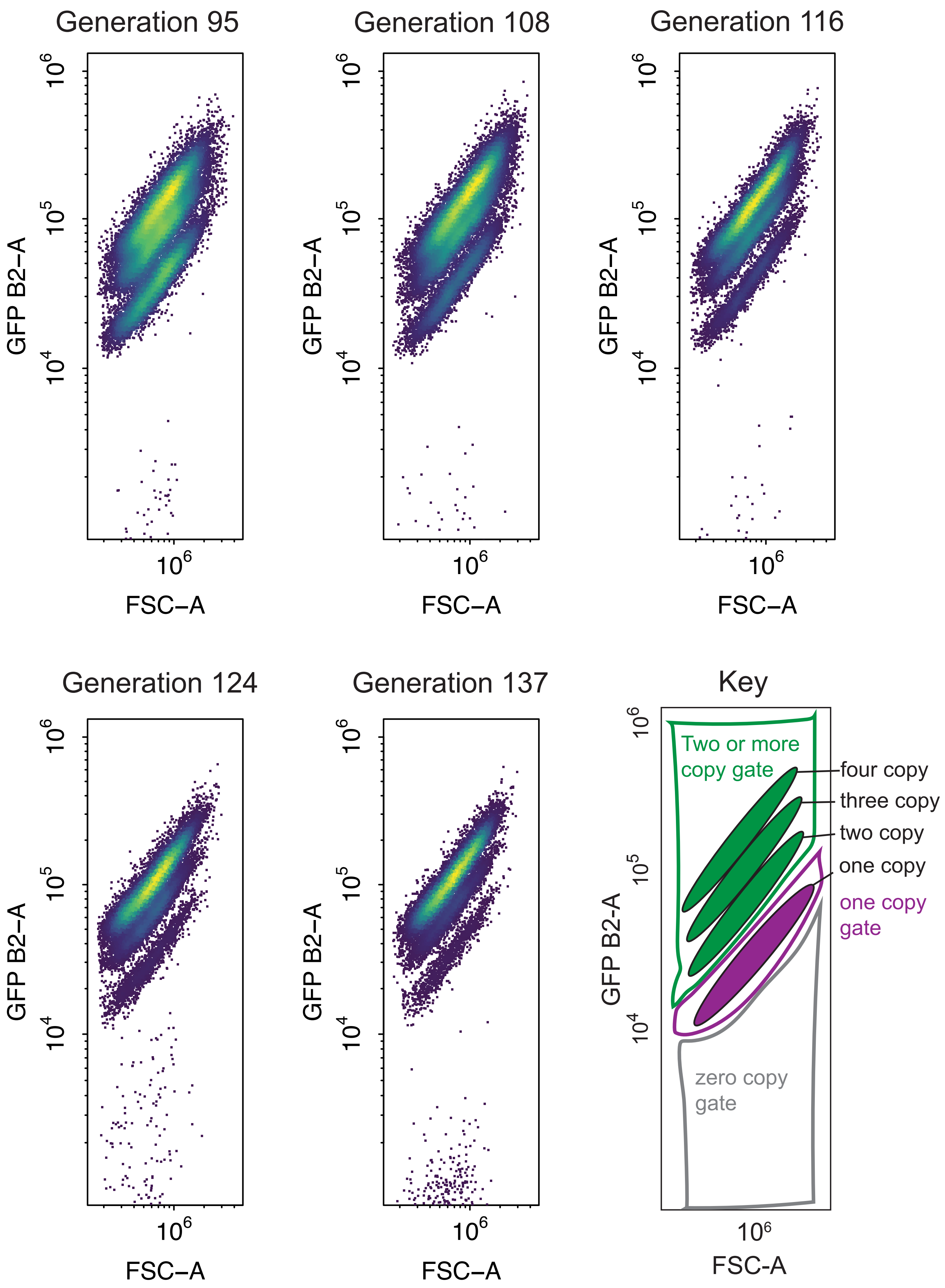

ALLΔ population 1

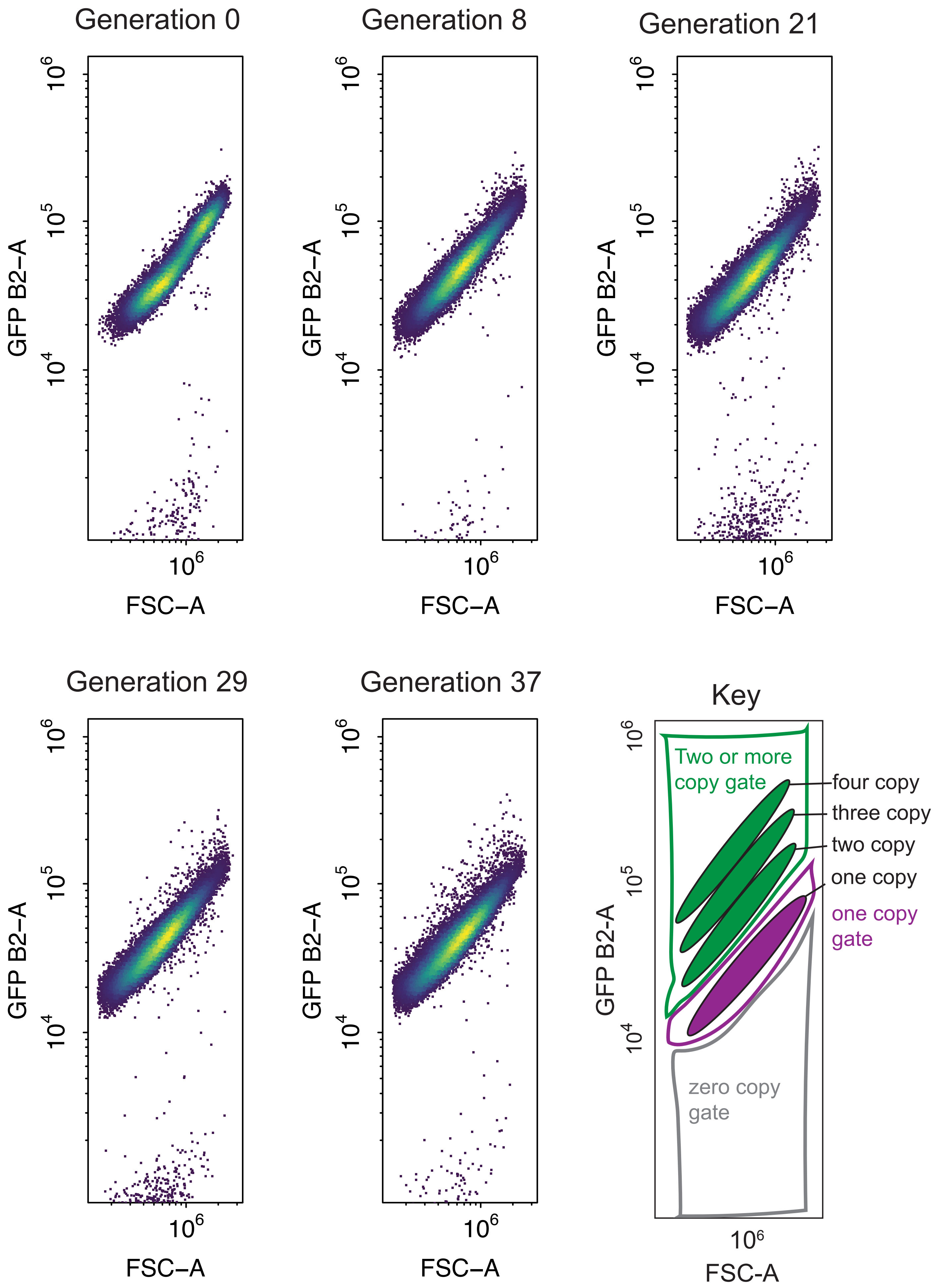

ALLΔ population 1

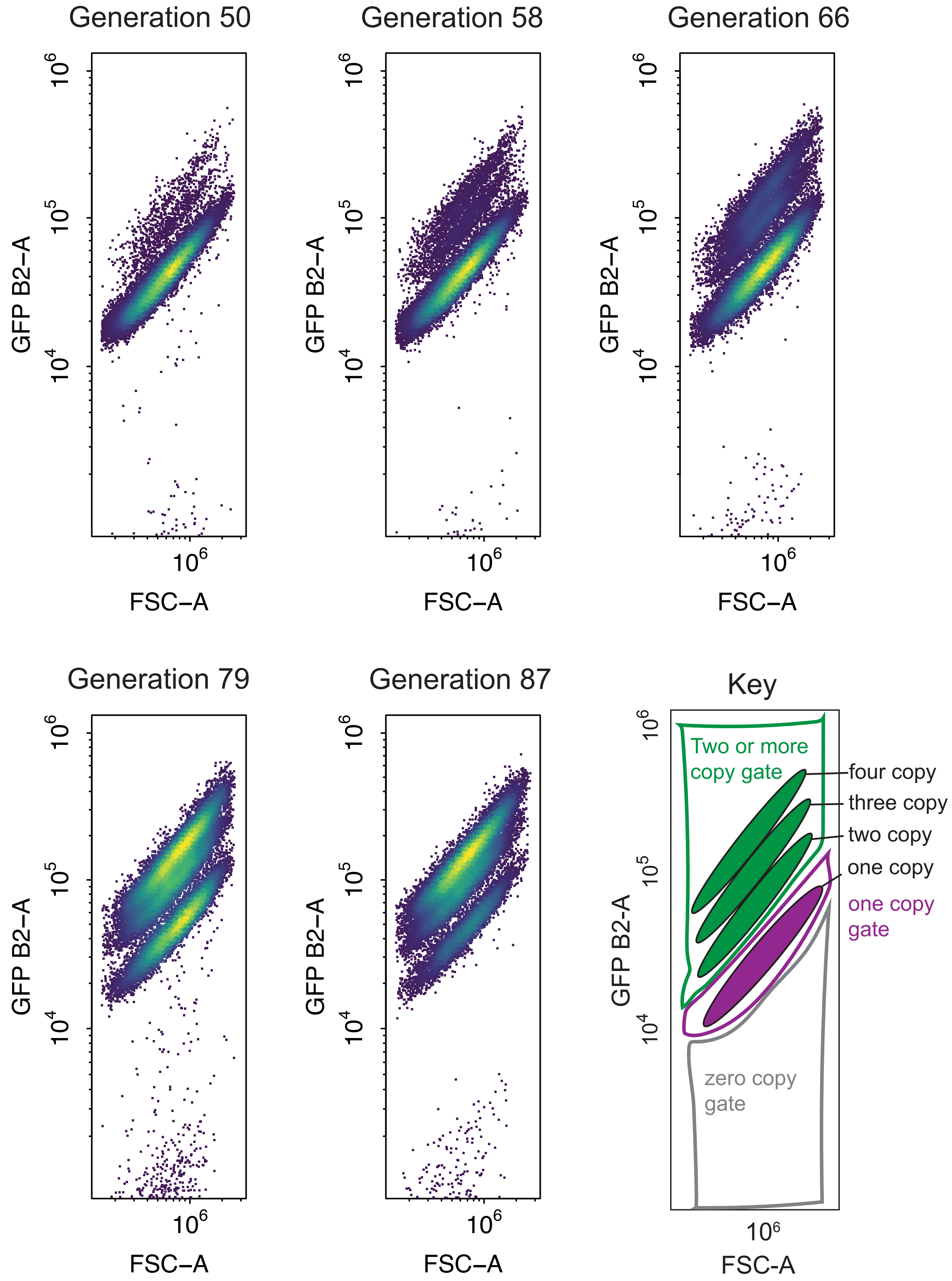

ALLΔ population 1

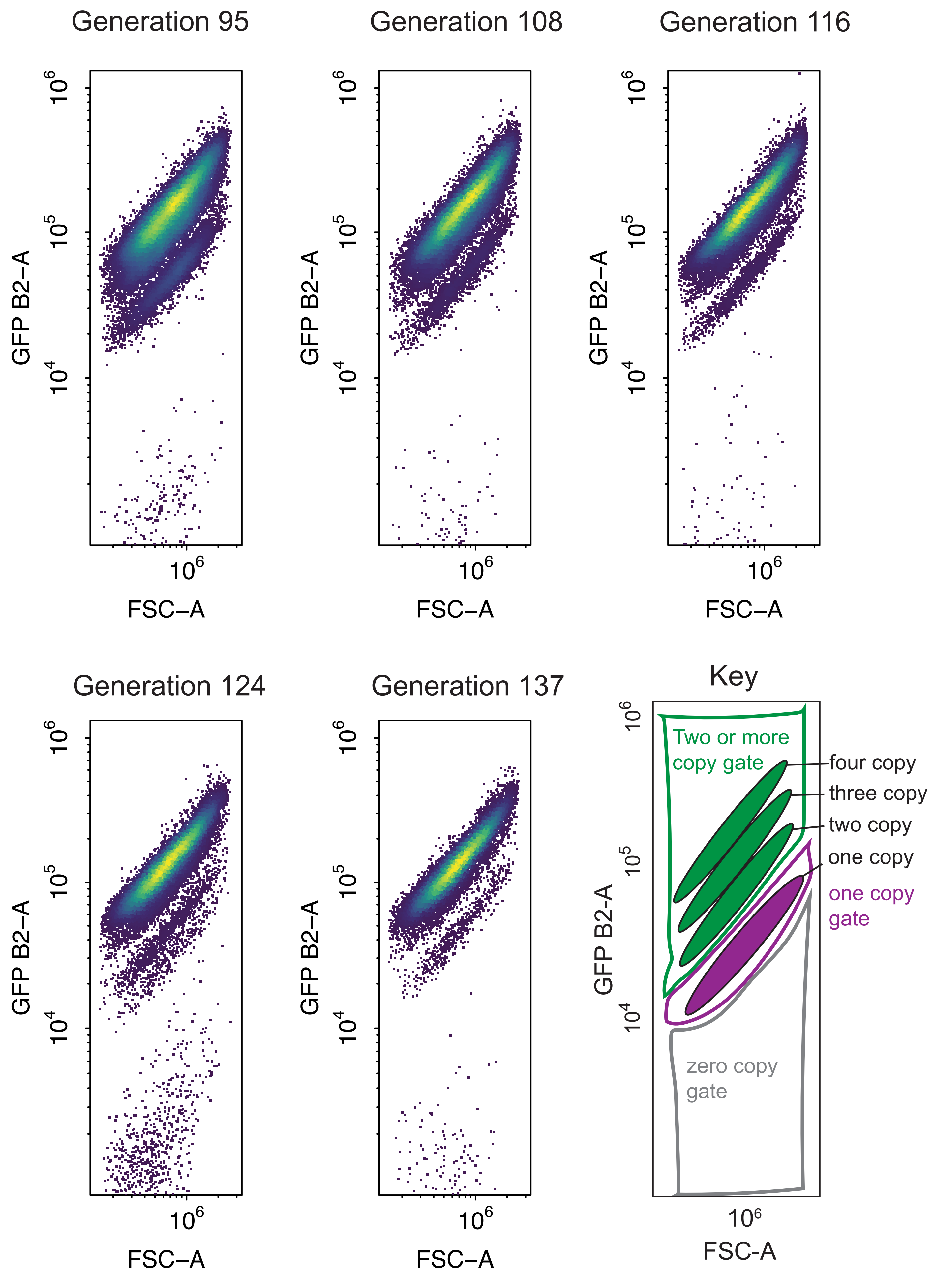

ALLΔ population 2

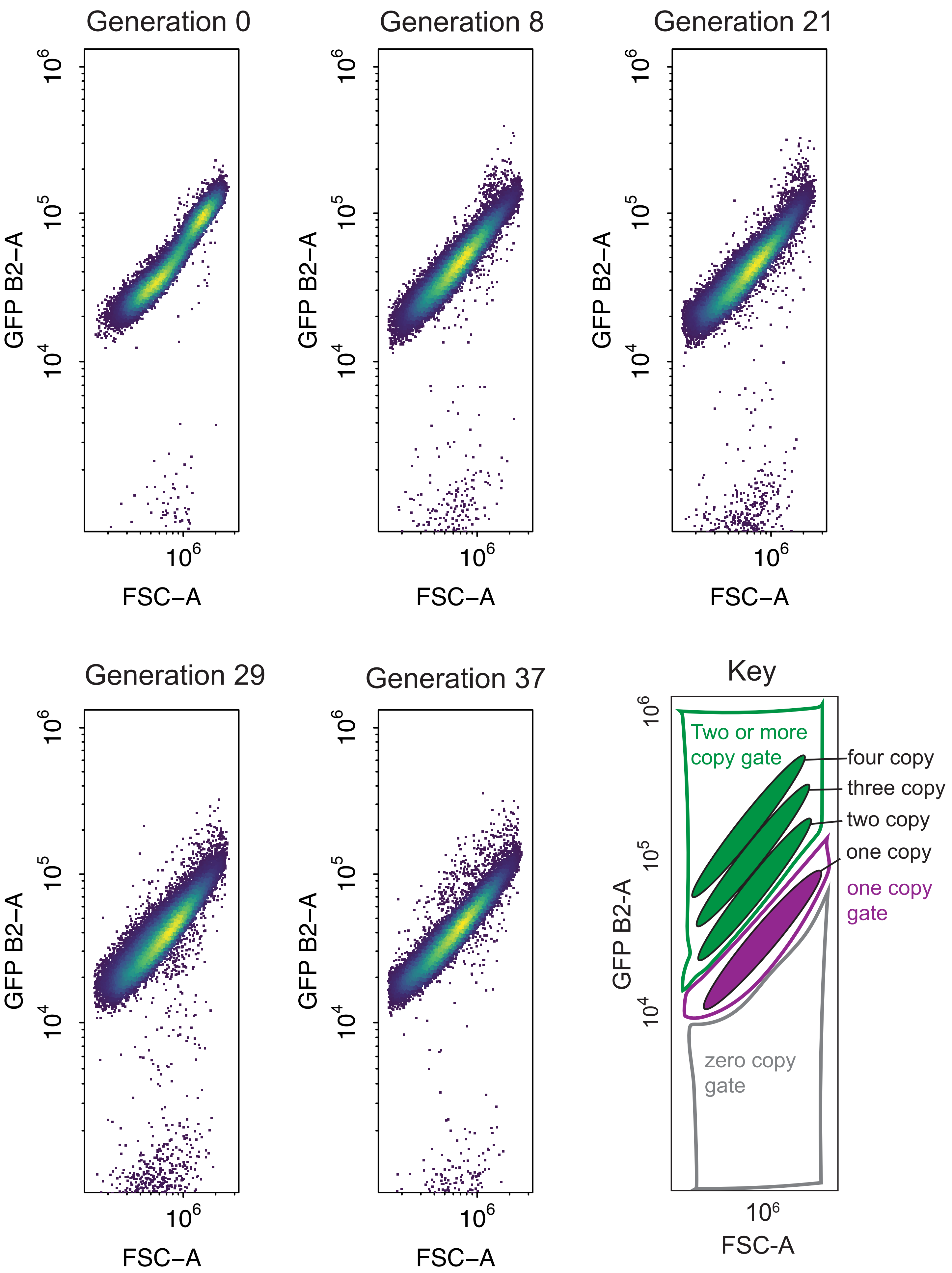

ALLΔ population 2

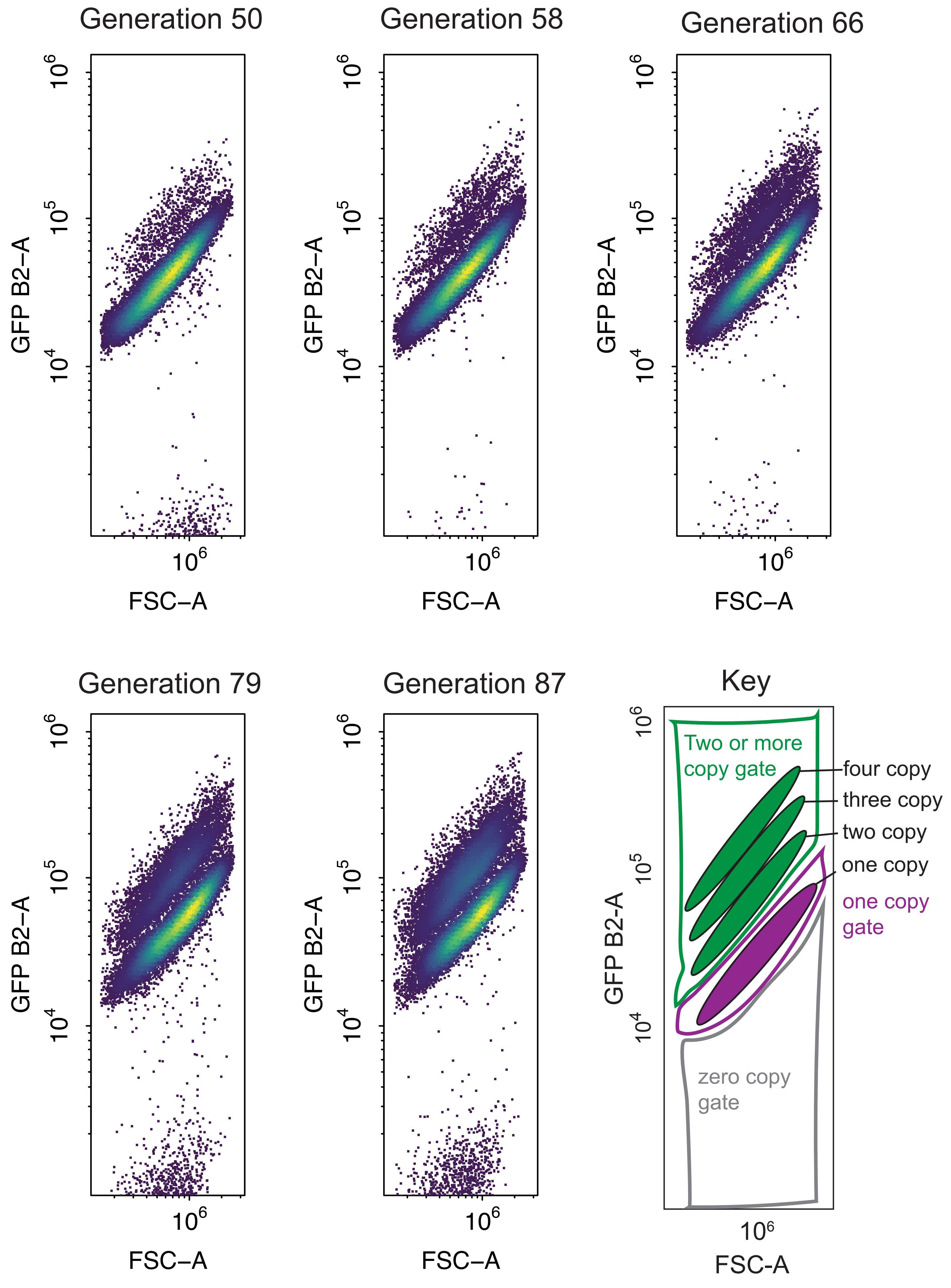

ALLΔ population 2

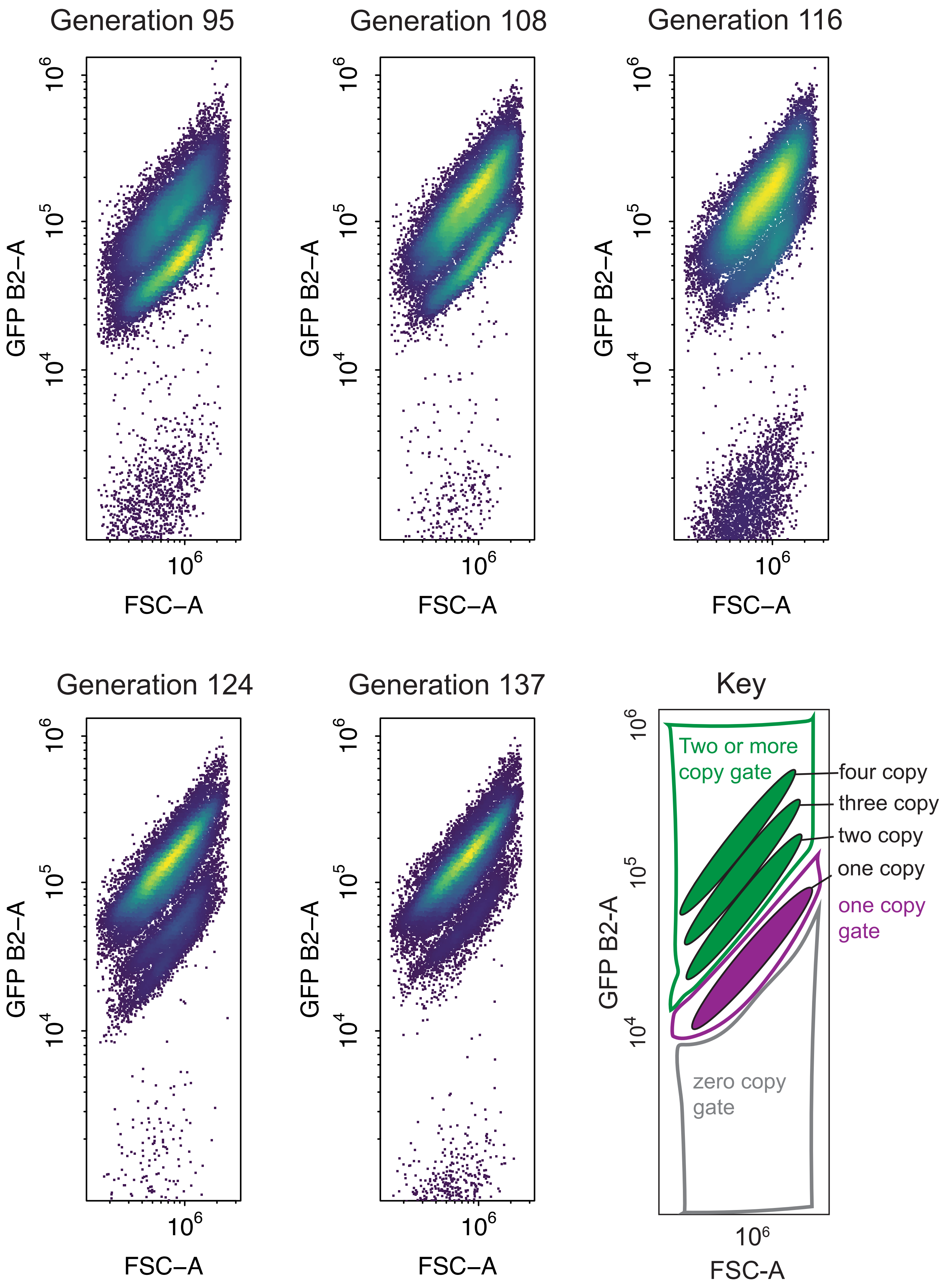

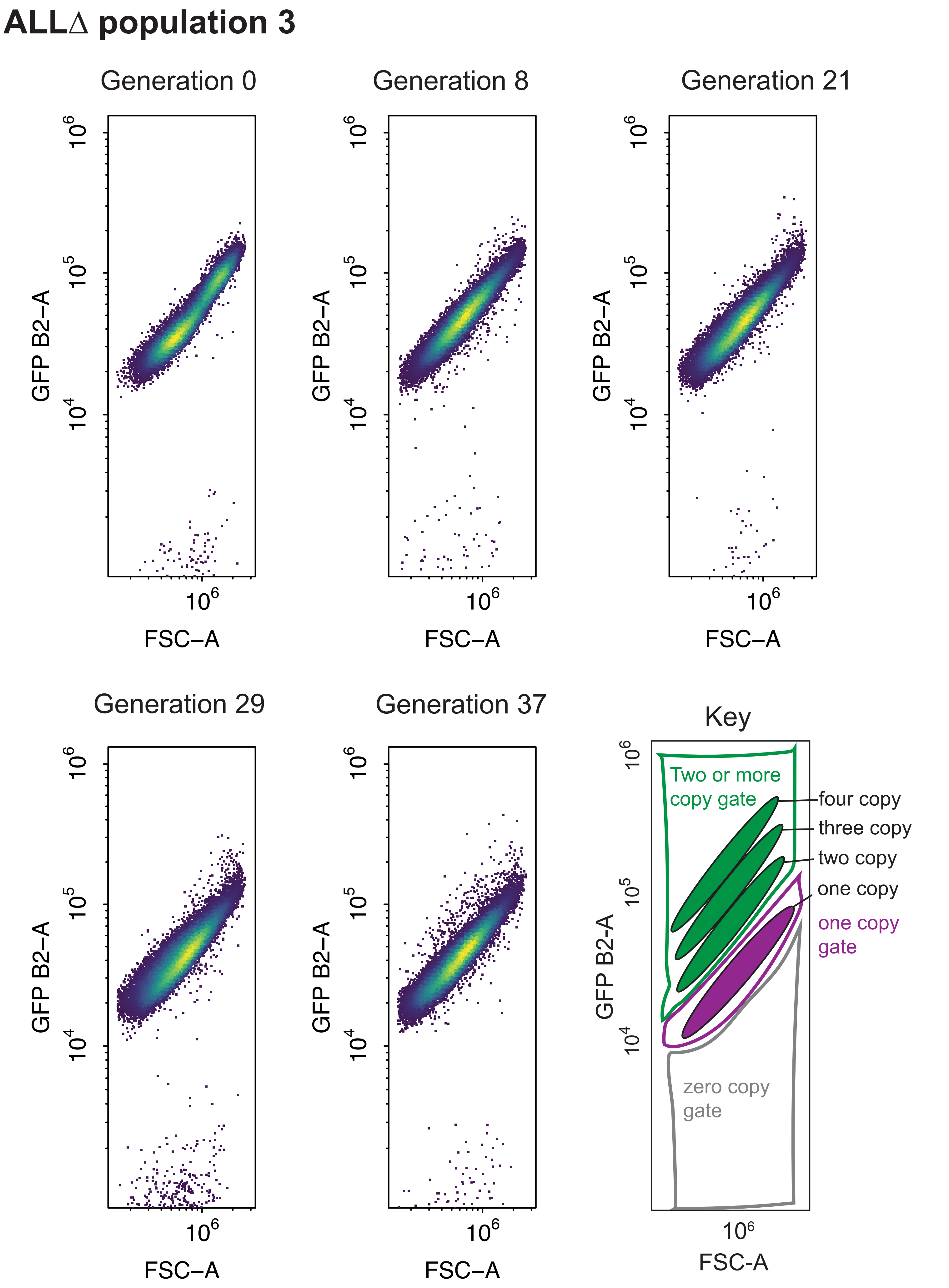
ALLΔ population 3
Generation 0
Generation 8
Generation 21
Generation 29
Generation 37
Key
GFP B2−A
FSC−A
10^4
10^5
10^6
Two or more copy gate
four copy
three copy
two copy
one copy
one copy gate
zero copy gate

ALLΔ population 3

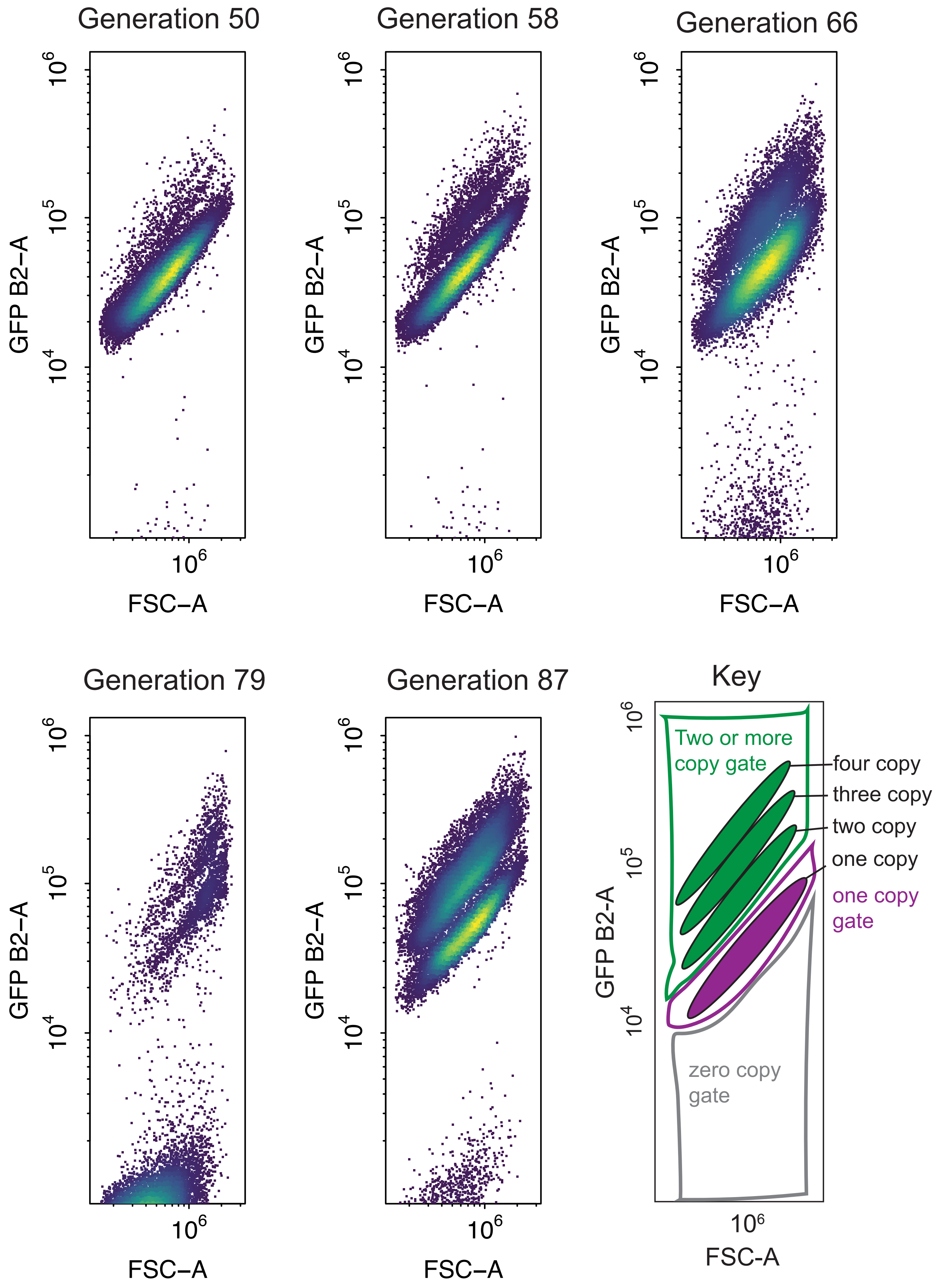

ALLΔ population 3

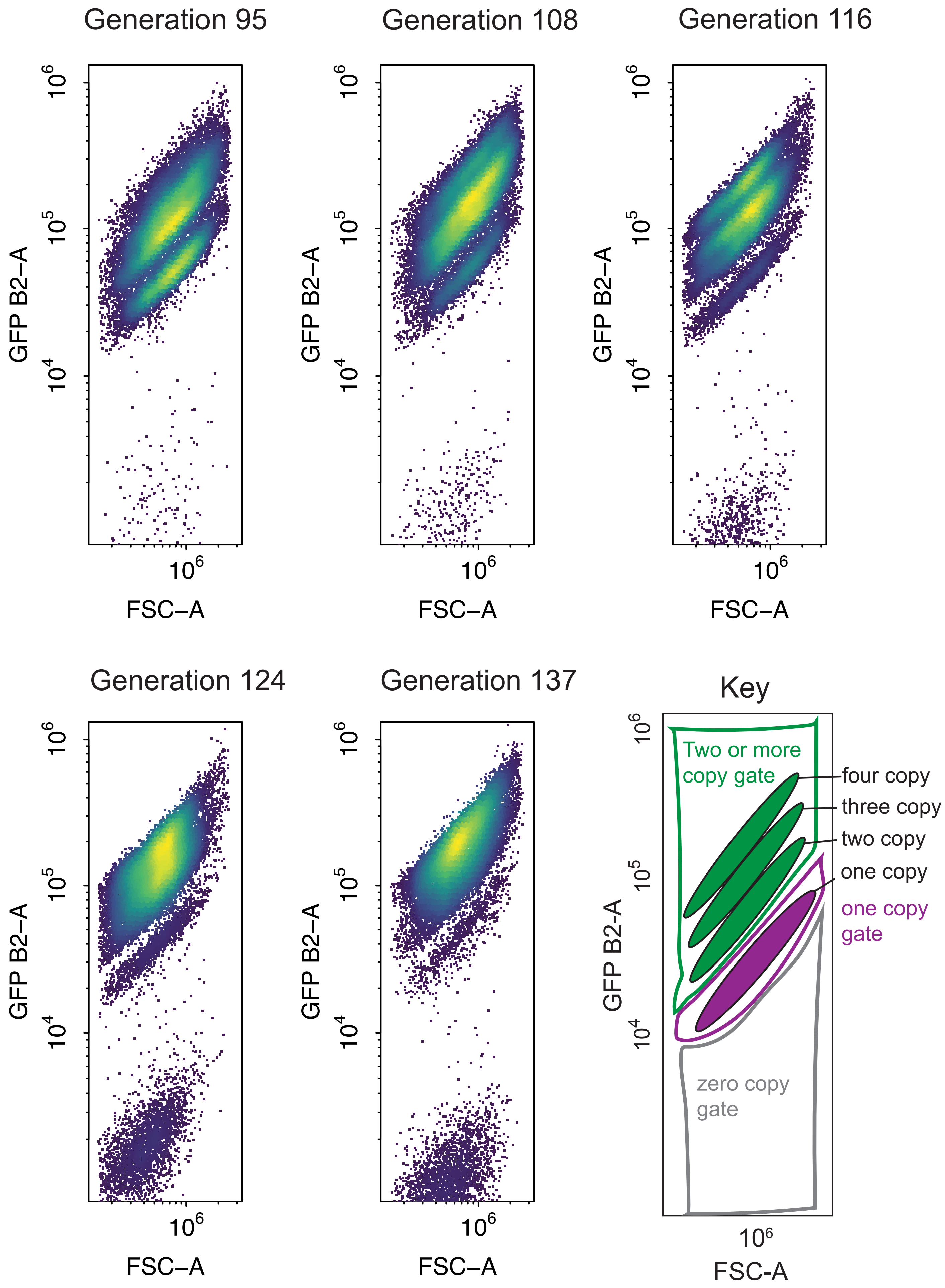

ALLΔ population 4

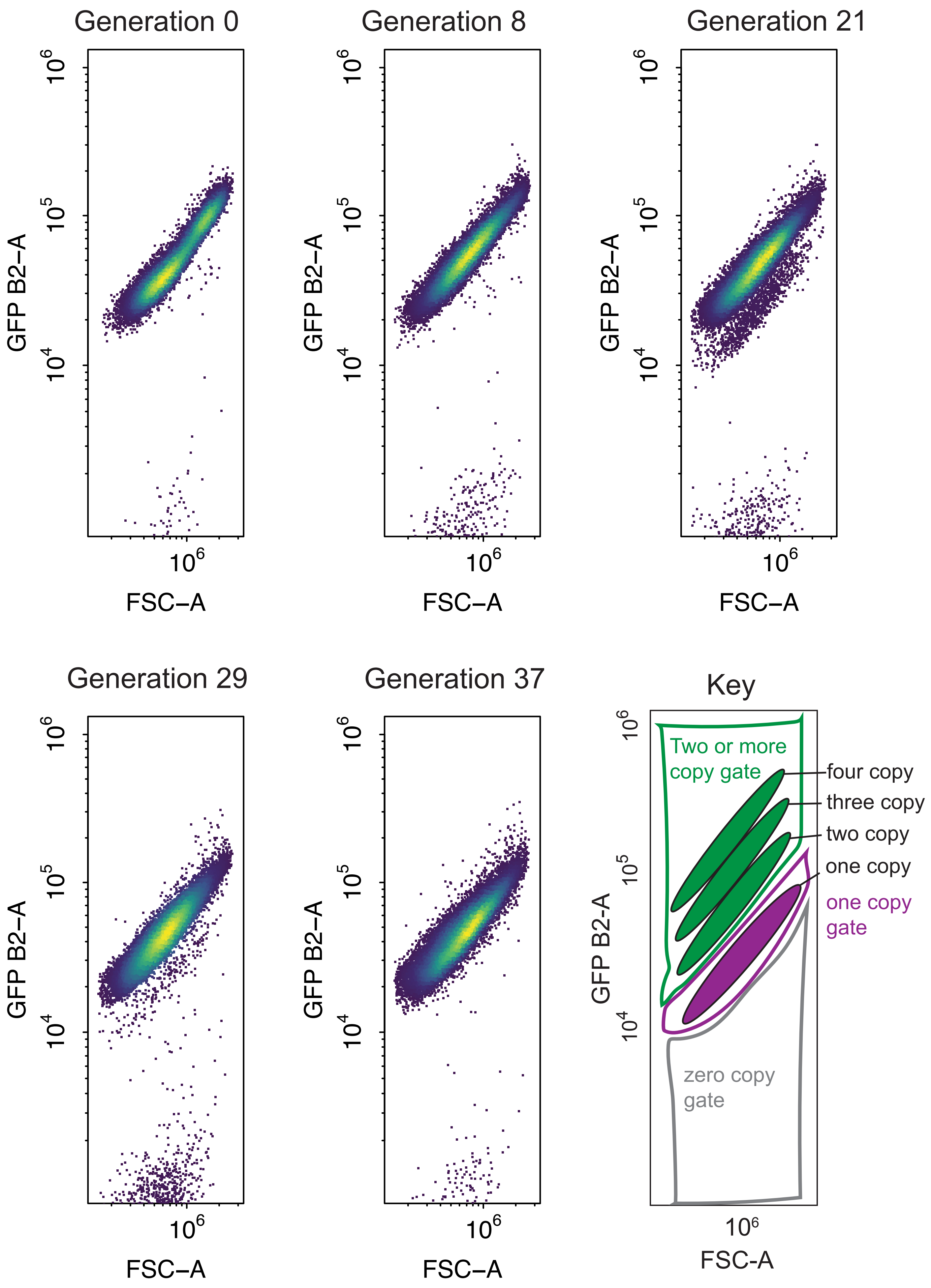

ALLΔ population 4

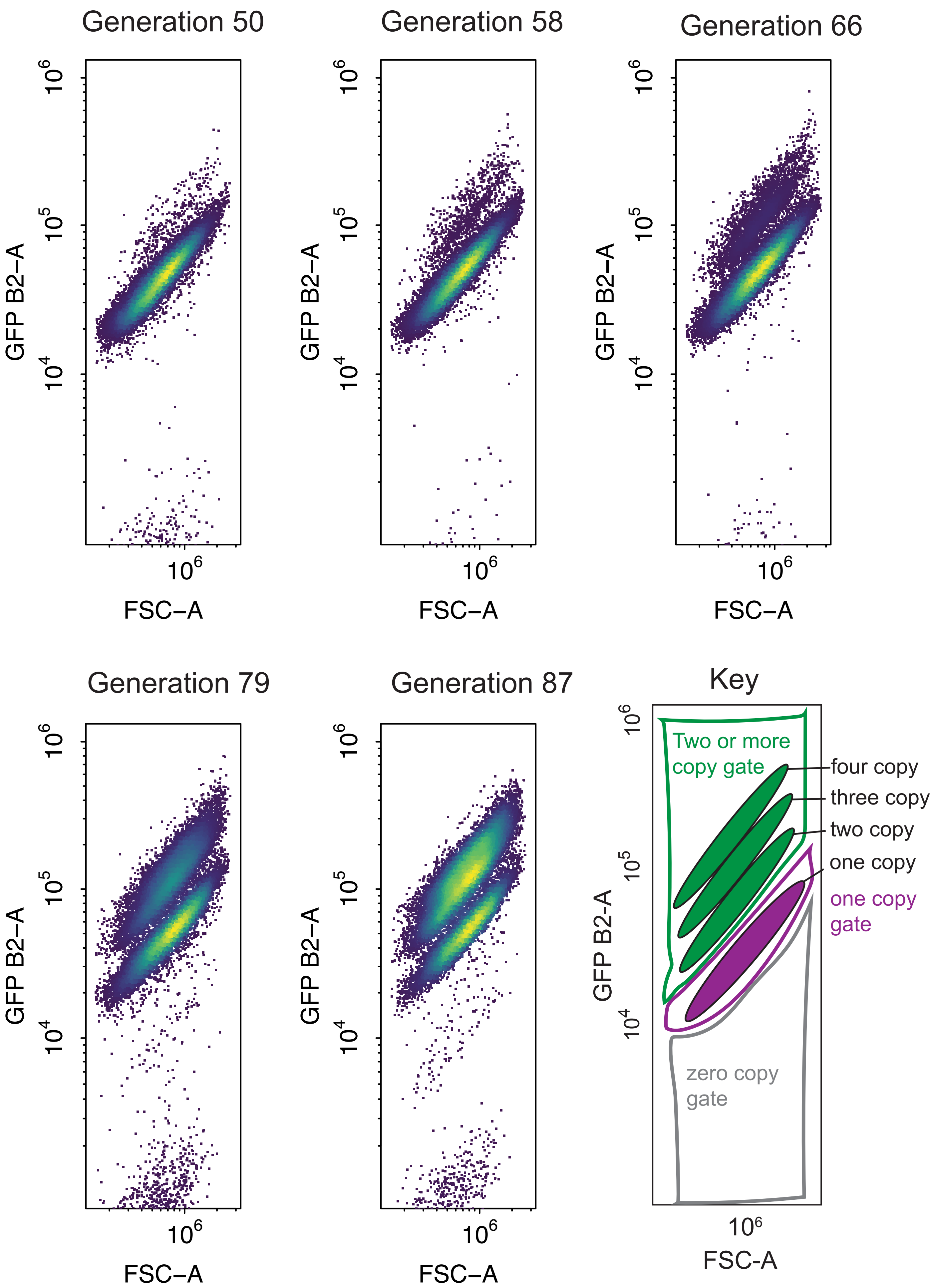

ALLΔ population 4

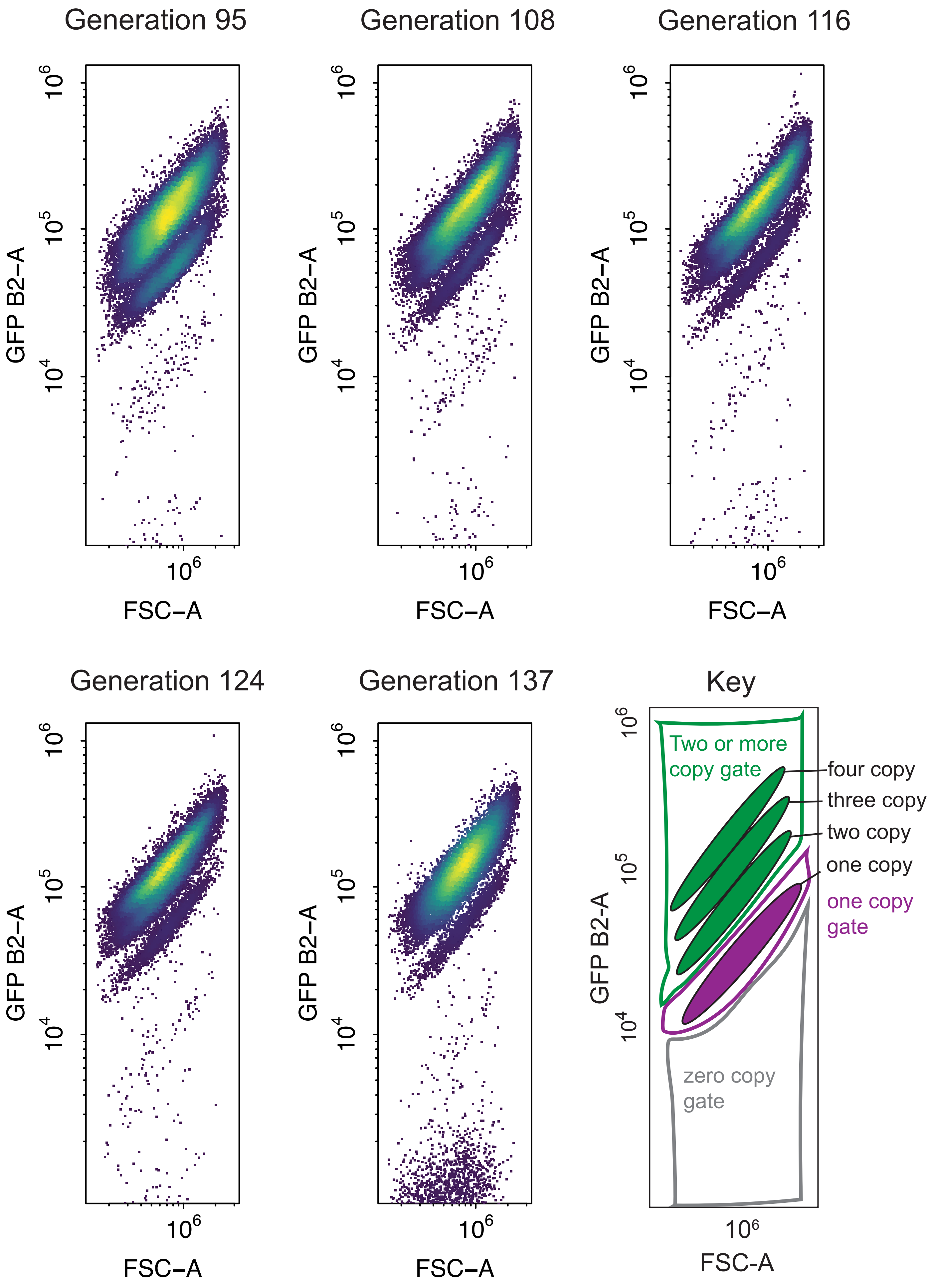

ALLΔ population 5

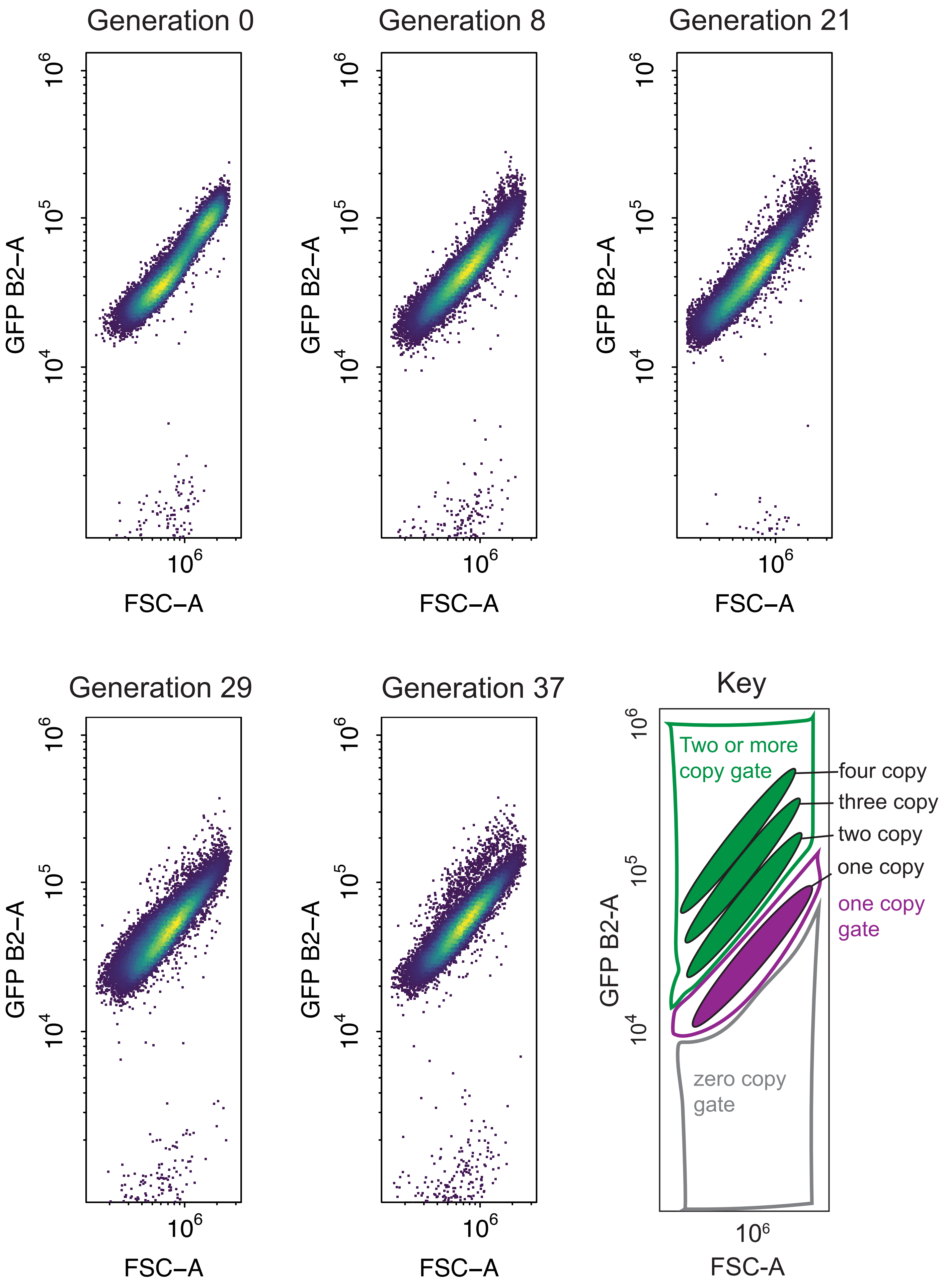

ALLΔ population 5

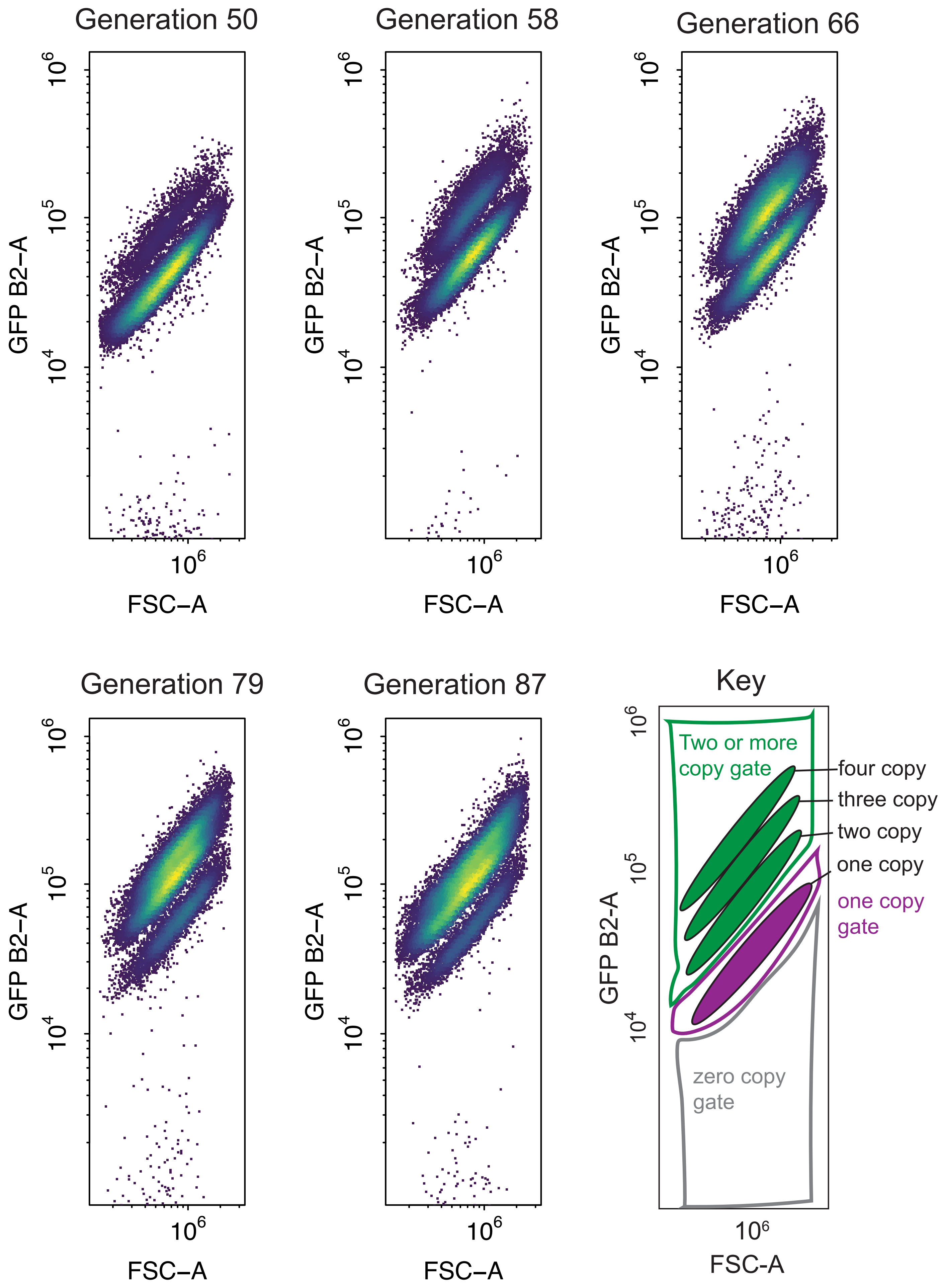

ALLΔ population 5

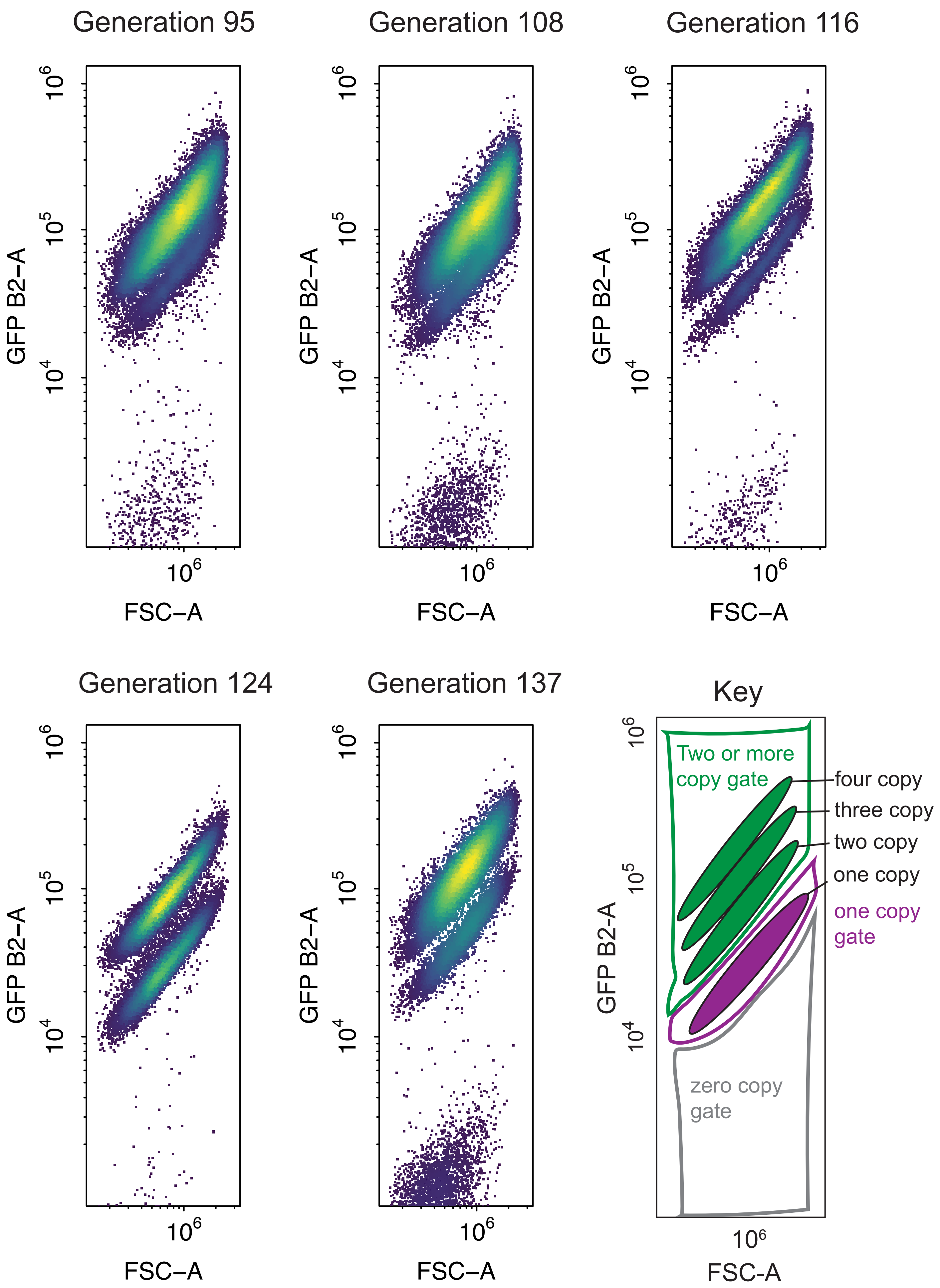

ALLΔ population 6

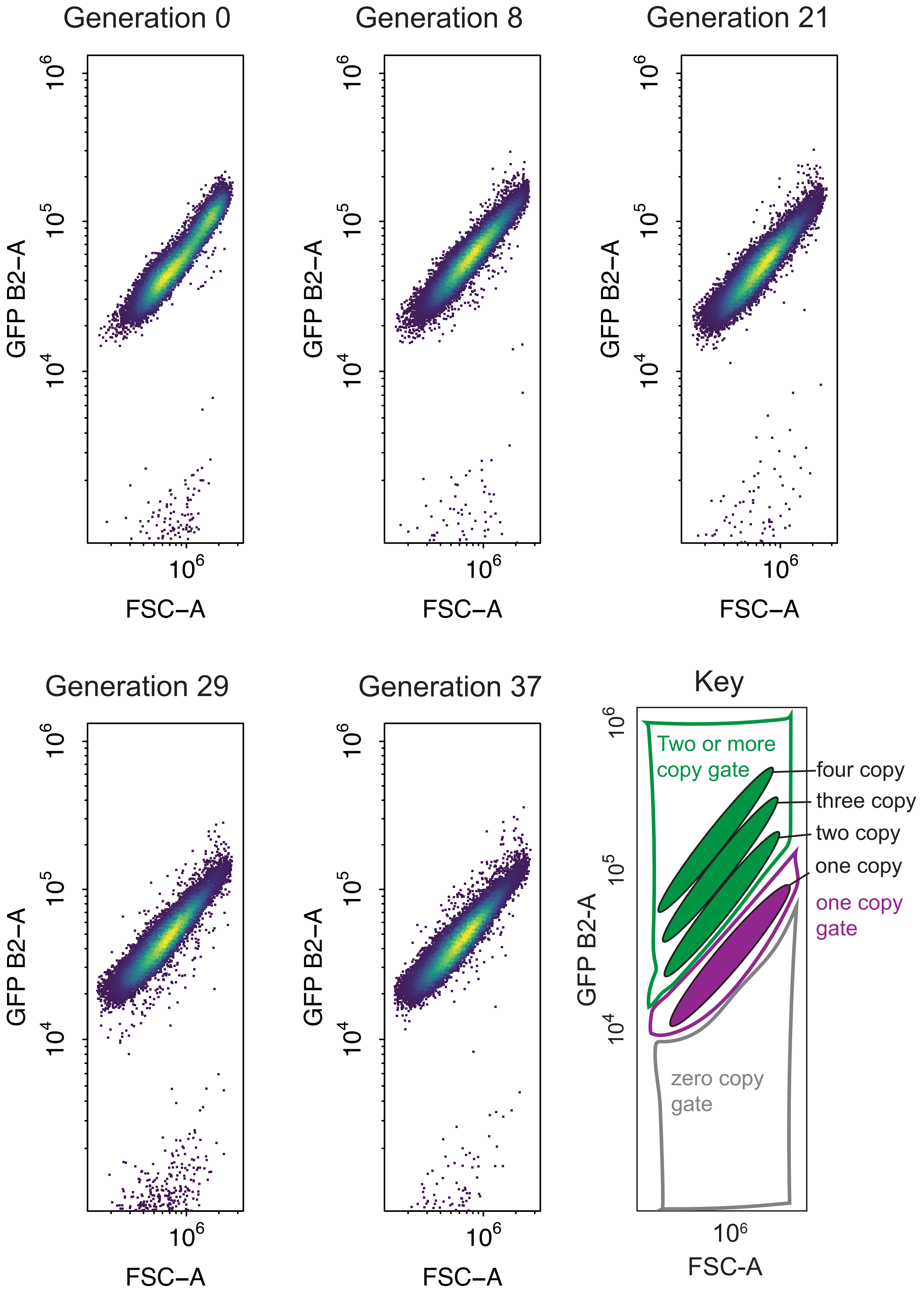

ALLΔ population 6

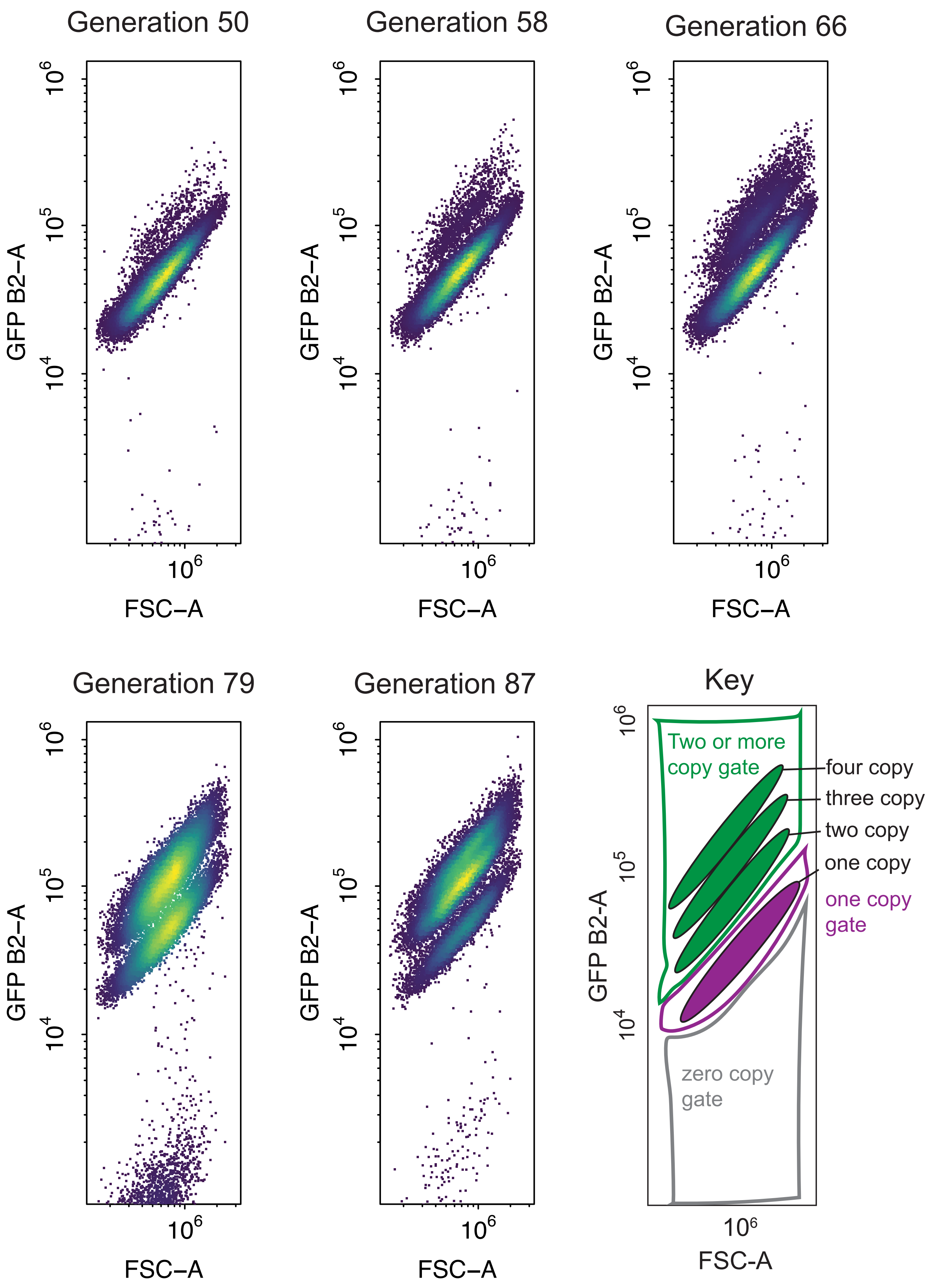

ALLΔ population 6

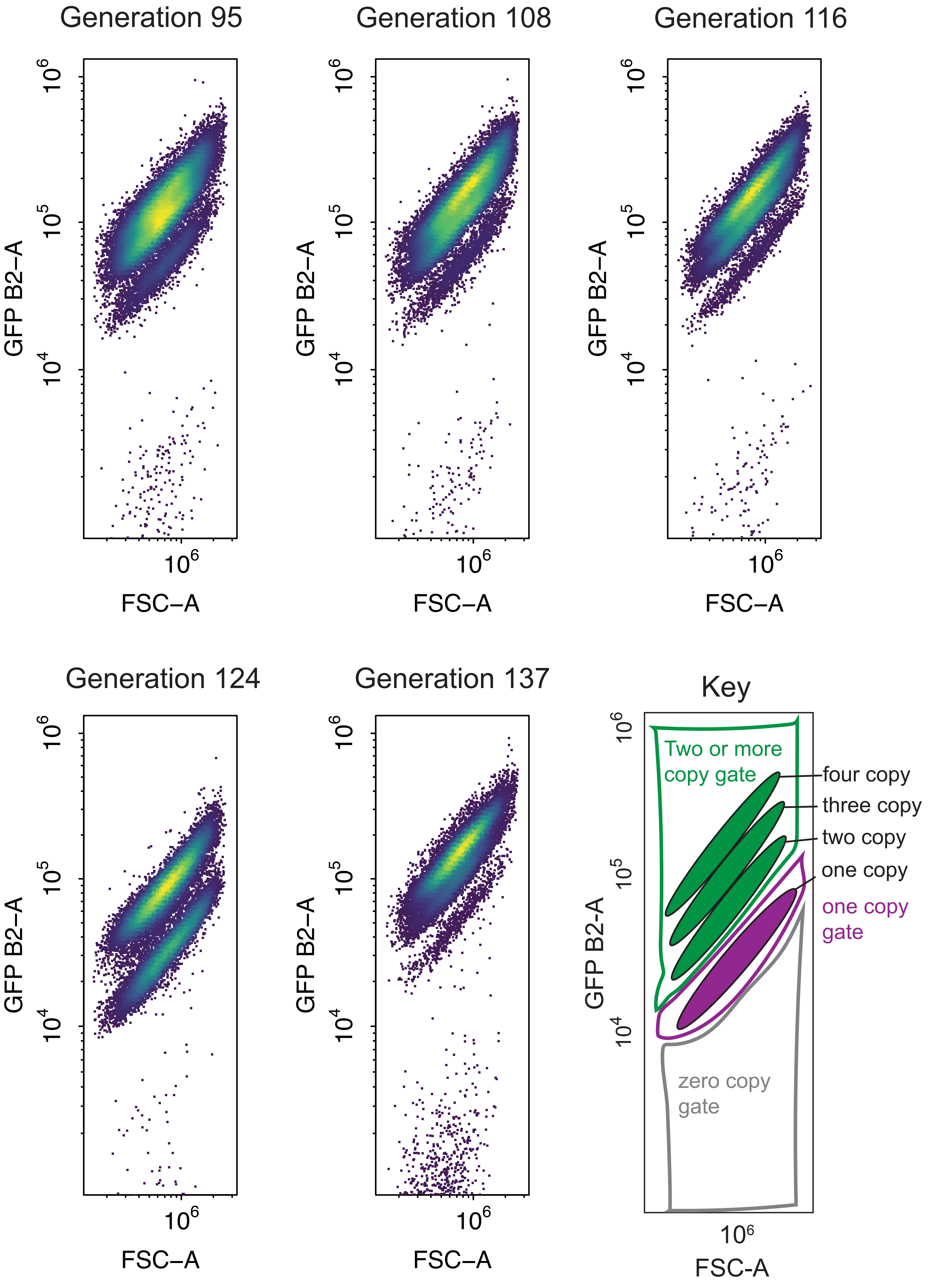

ALLΔ population 7

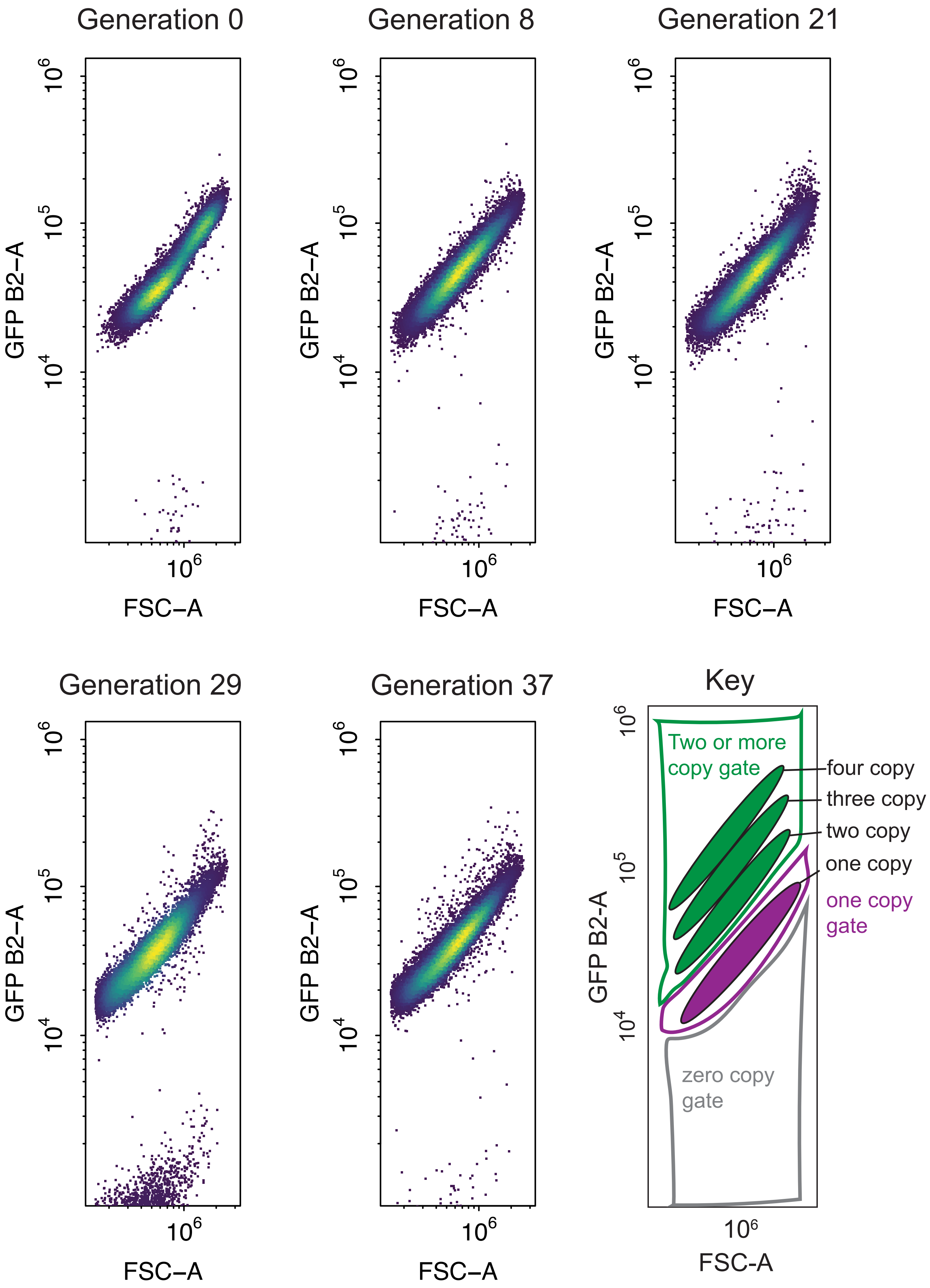

ALLΔ population 7

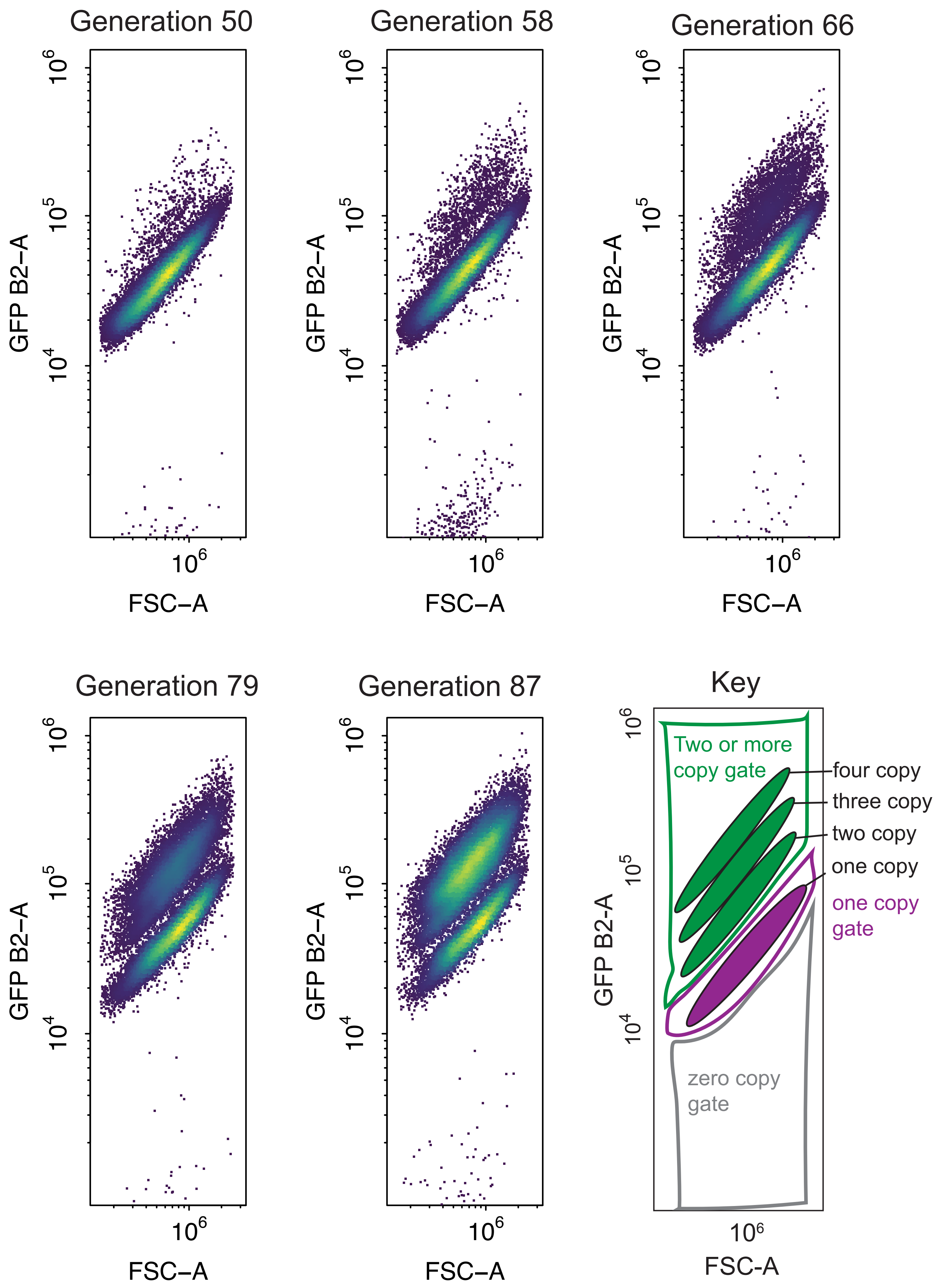

ALLΔ population 7

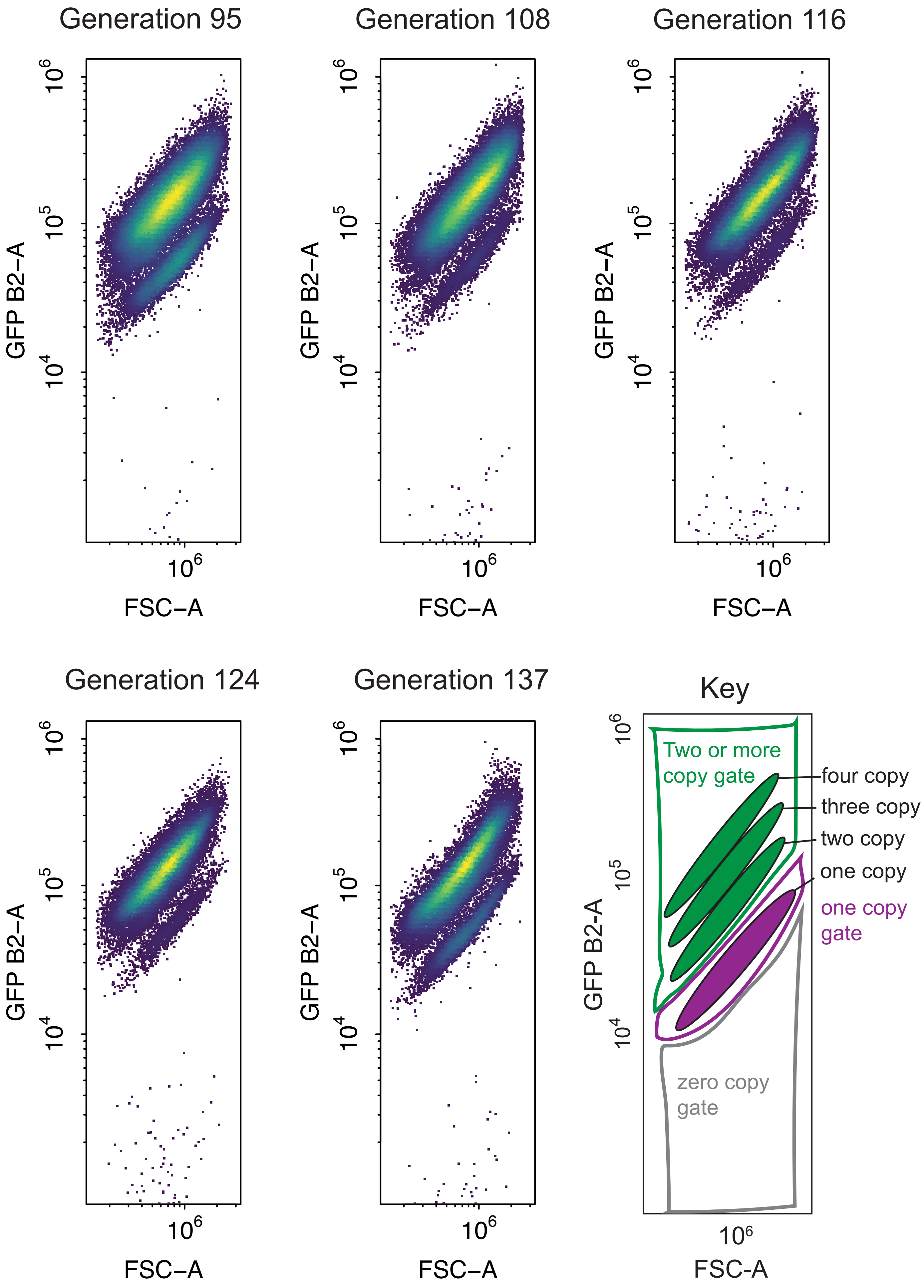

ALLΔ population 8

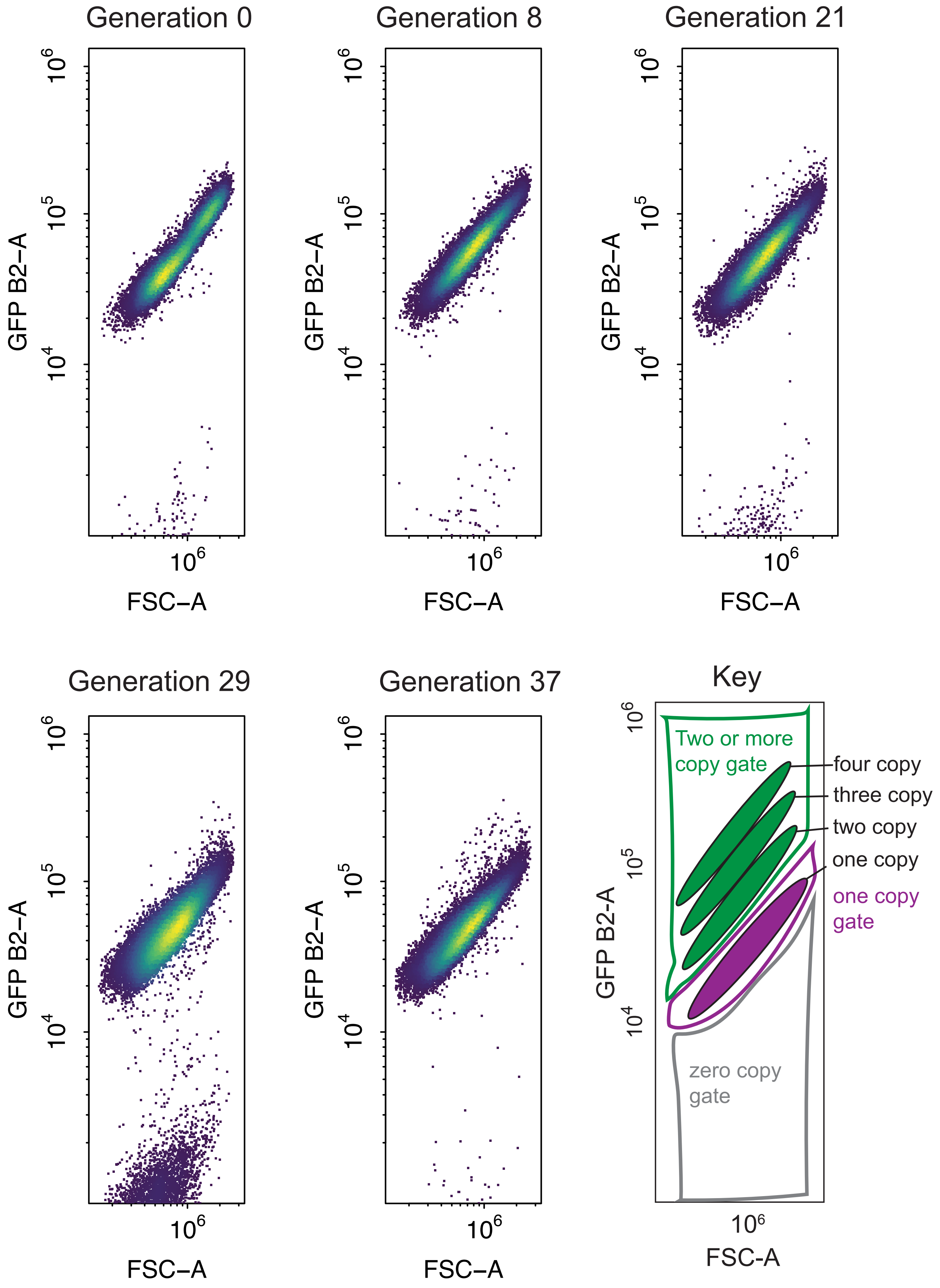

ALLΔ population 8

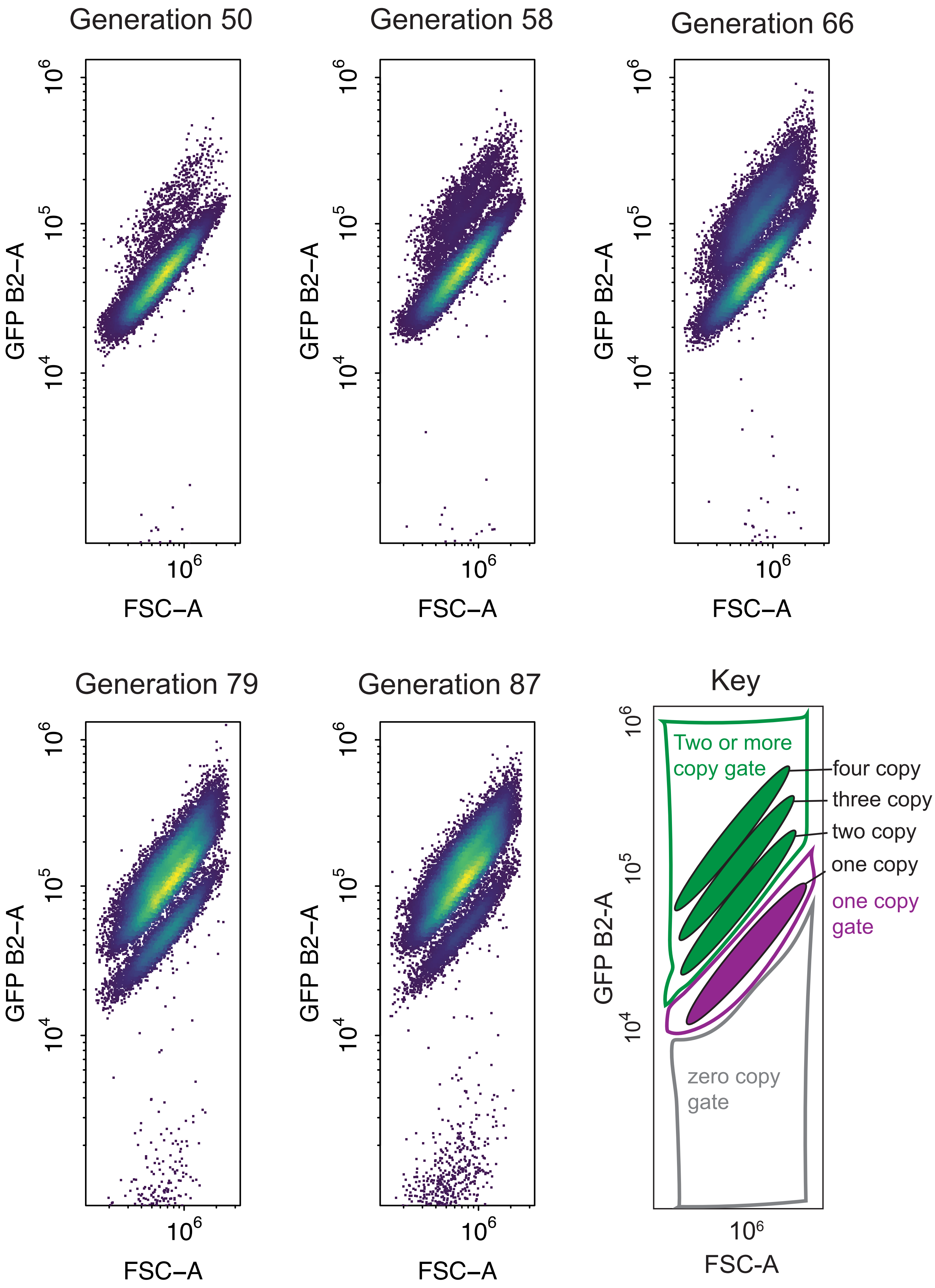

ALLΔ population 8

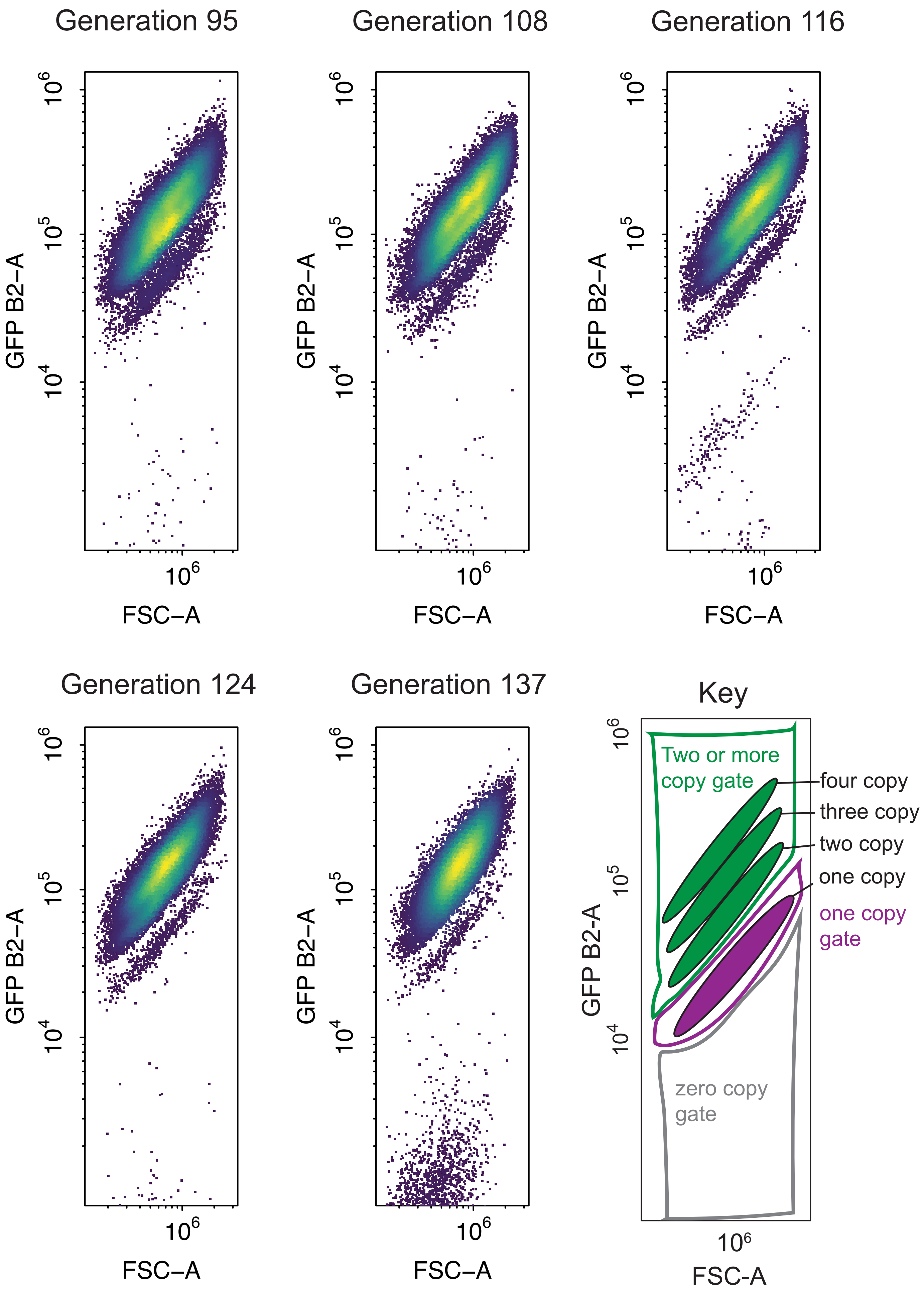

Supplement: Figure 2—source data 1. — FSC-A is forward scatter-area which is a proxy for cell size. GFP fluorescence was measured using the B2-A channel in arbitrary units. Hierarchical gating was performed to identify zero-, one-, and two-or-more-copy populations. Within the two-or-more copy gate, distinct subpopulations formed consistent with having a two-, three-, four- copies of GFP. [file elife-98934-fig2-data1.pdf]
