# Supplementary figures and images for: Template switching during DNA replication is a prevalent source of adaptive gene amplification
Source: eLife. 2025 Feb 3;13:RP98934. doi: 10.7554/eLife.98934 (PMC11790251; doi:10.7554/eLife.98934)

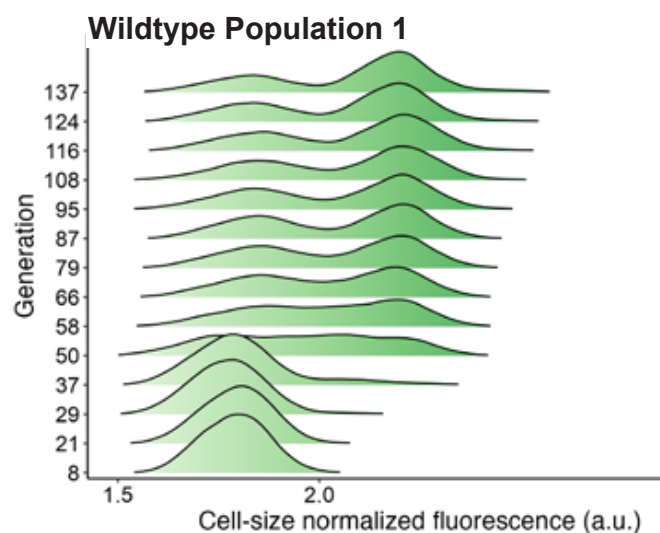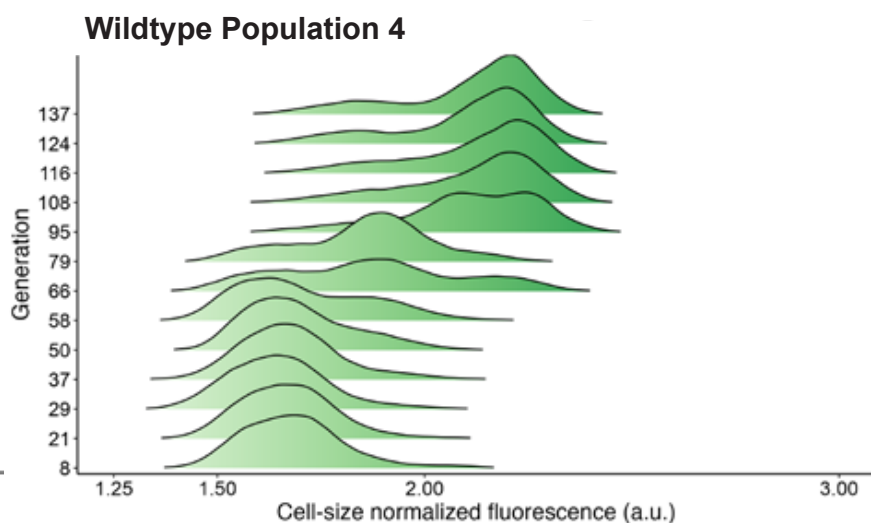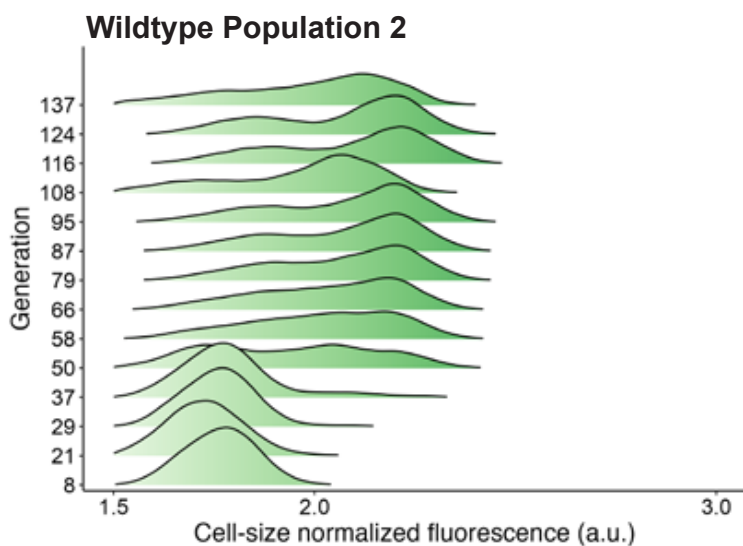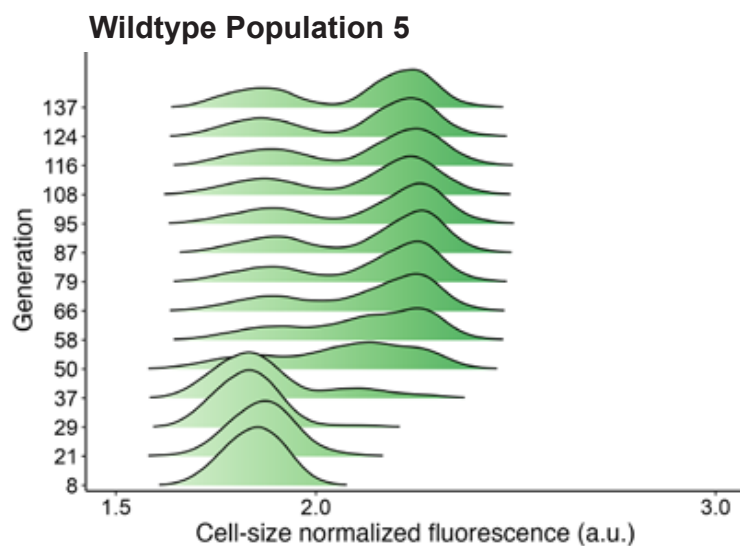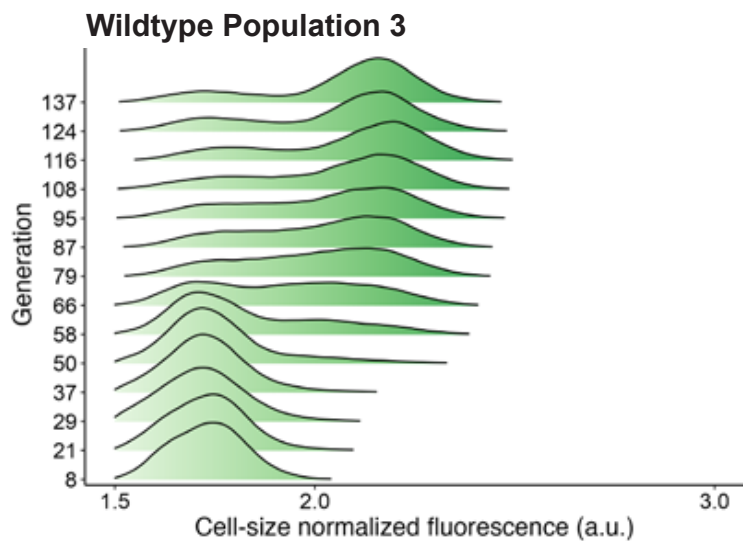

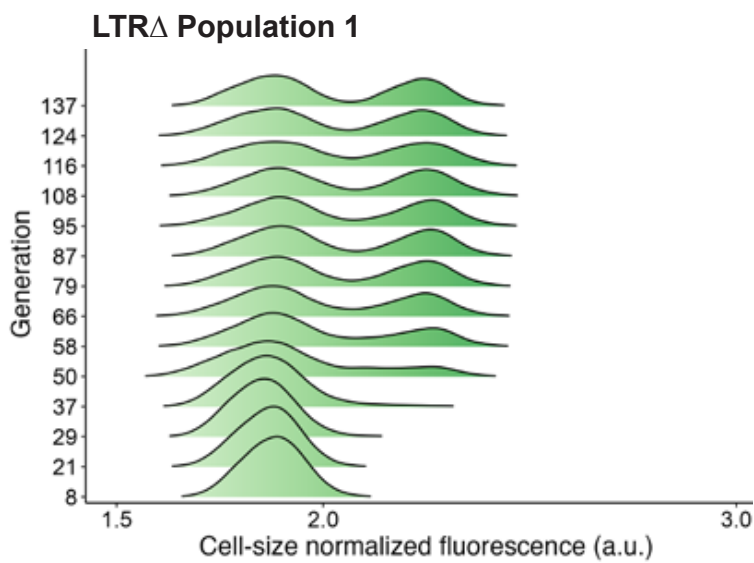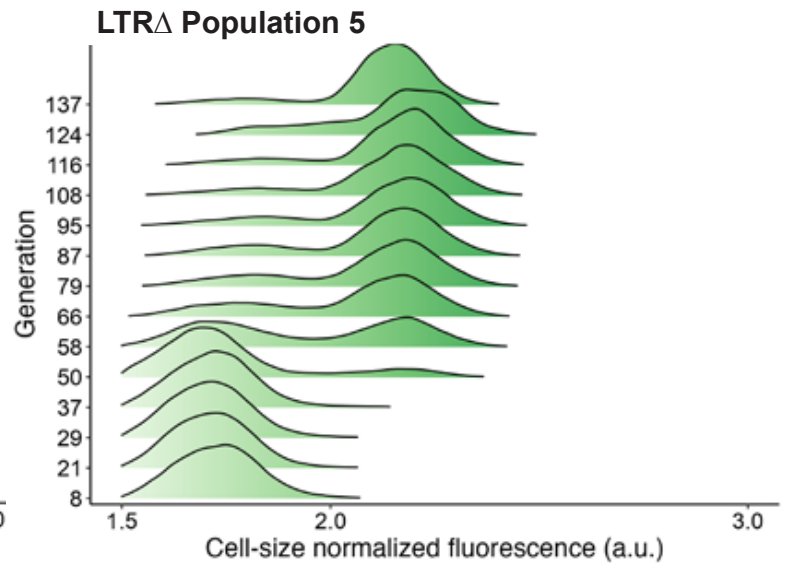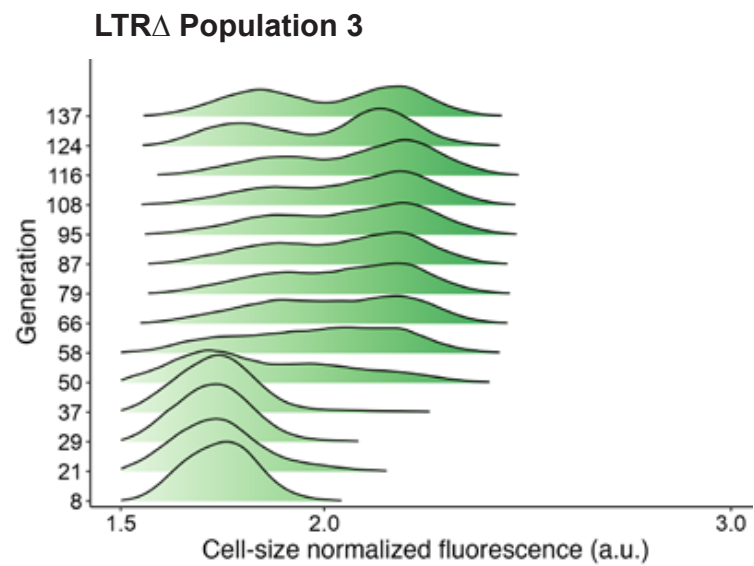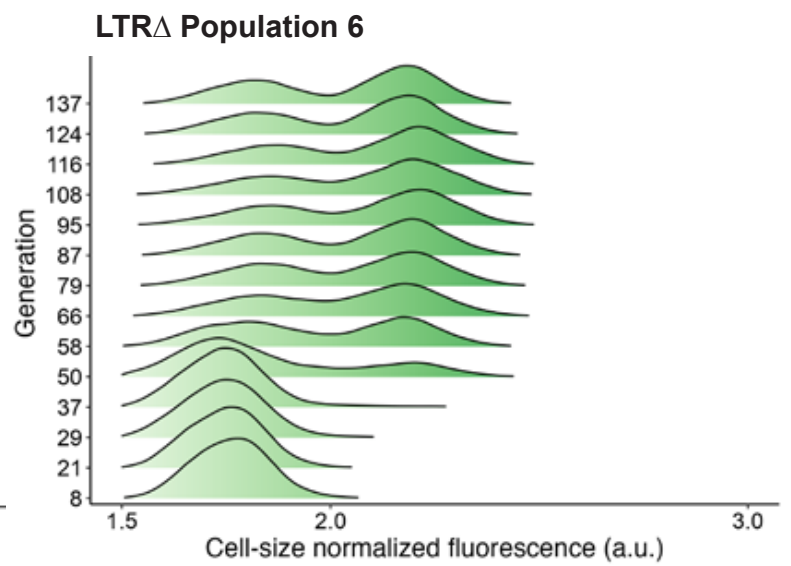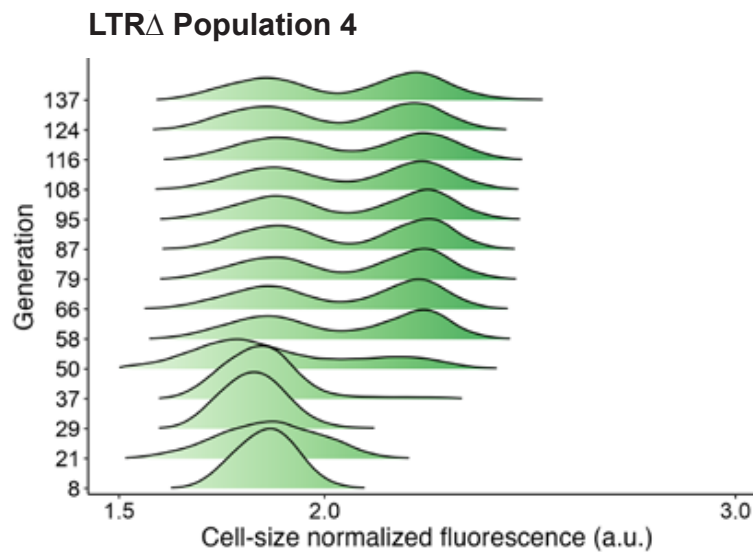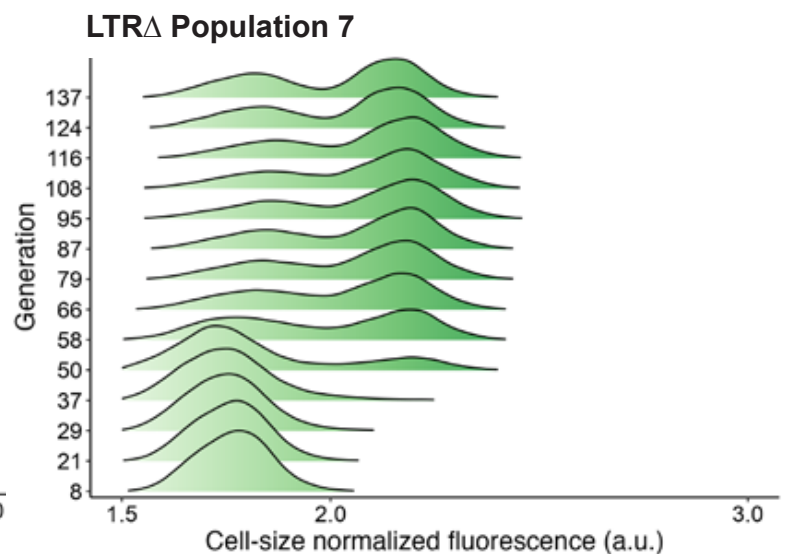

LTRΔ Population 8

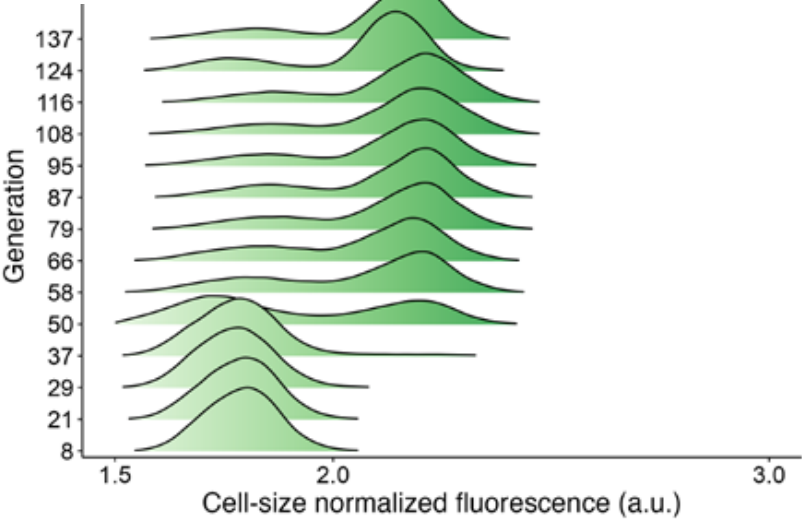

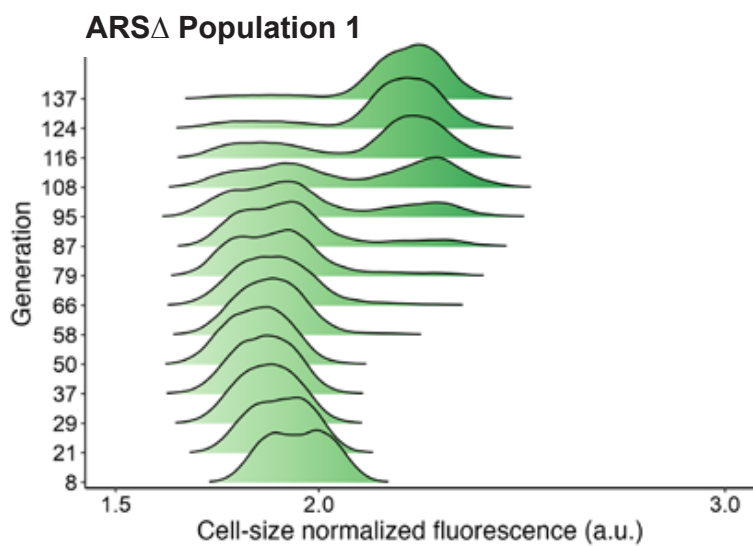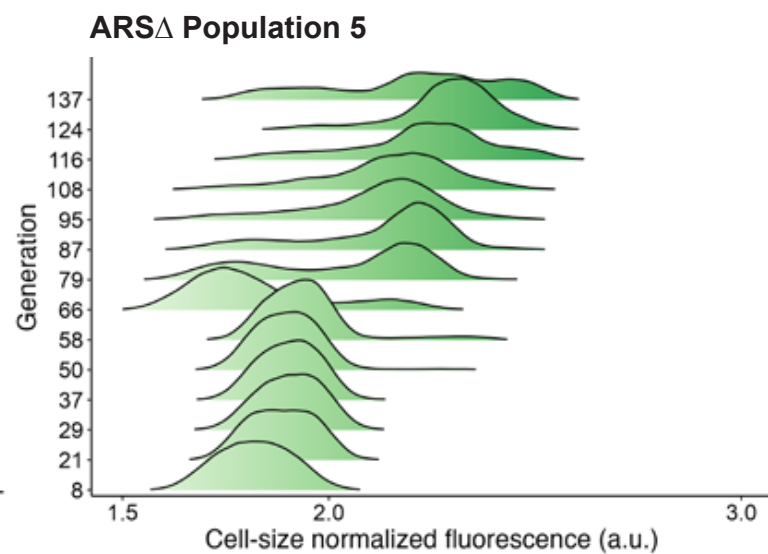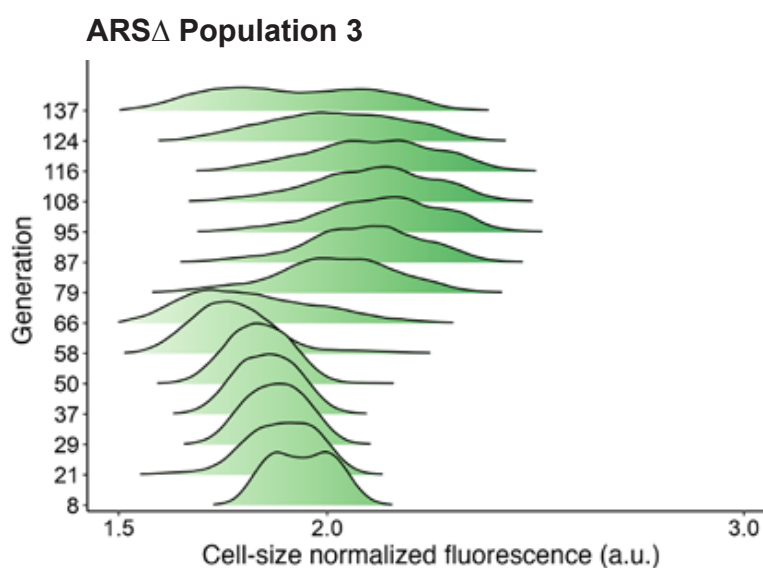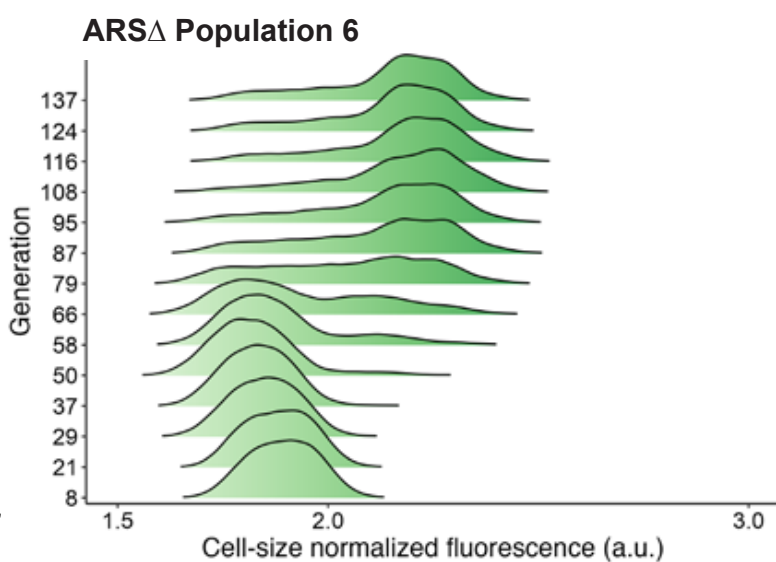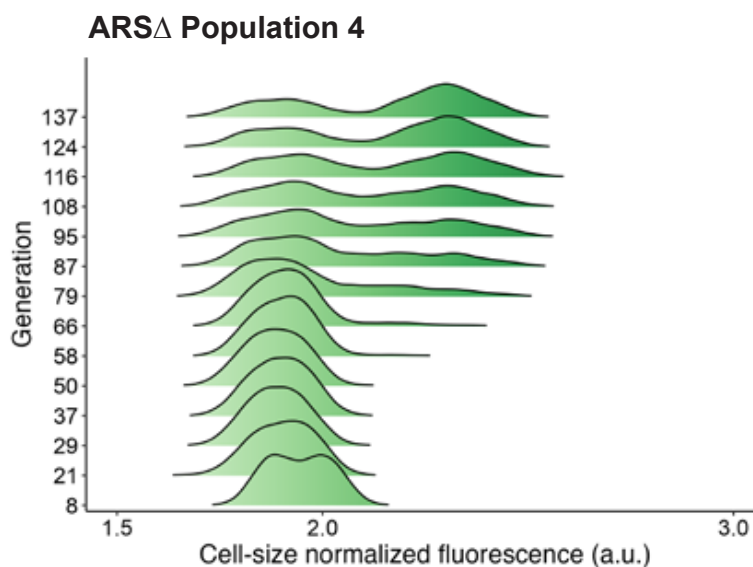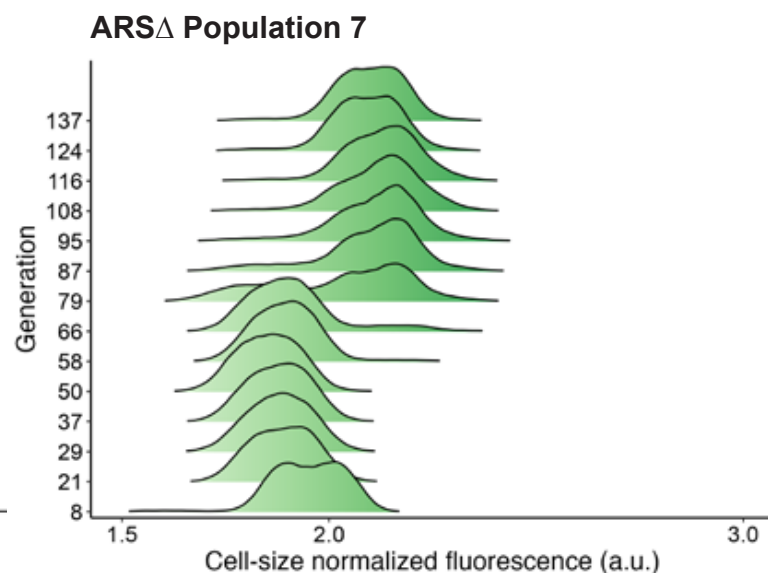

# ARS $\Delta$ Population 8

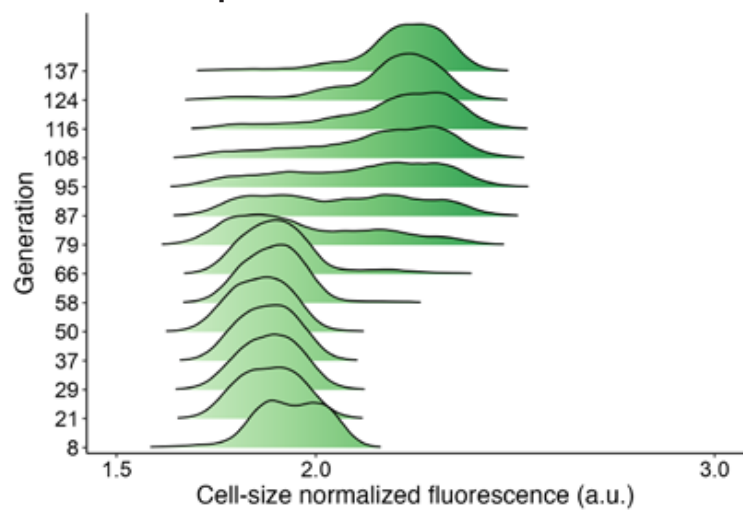

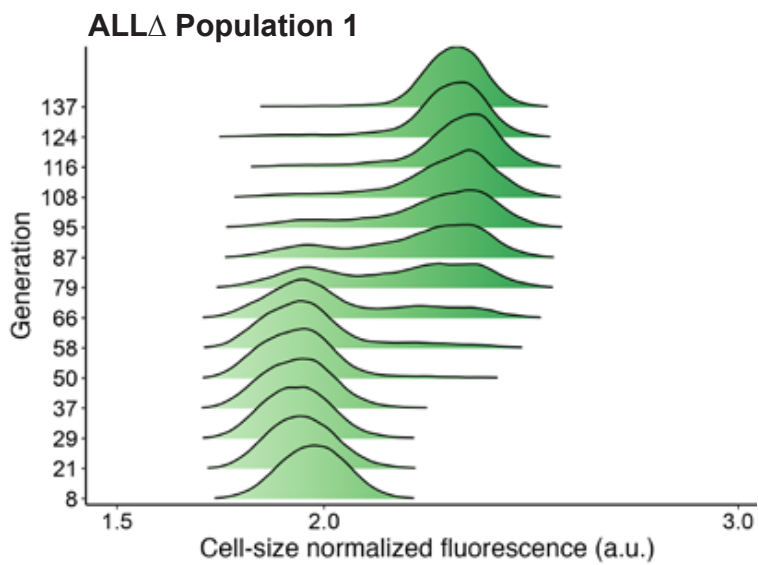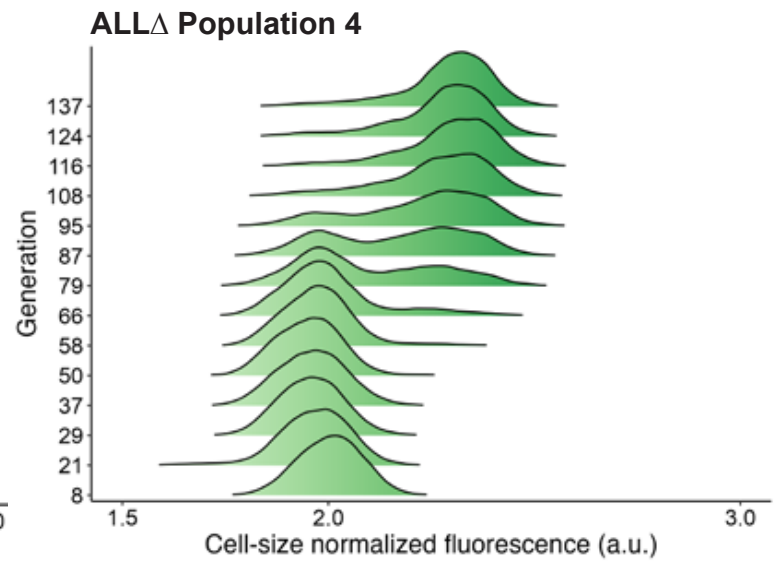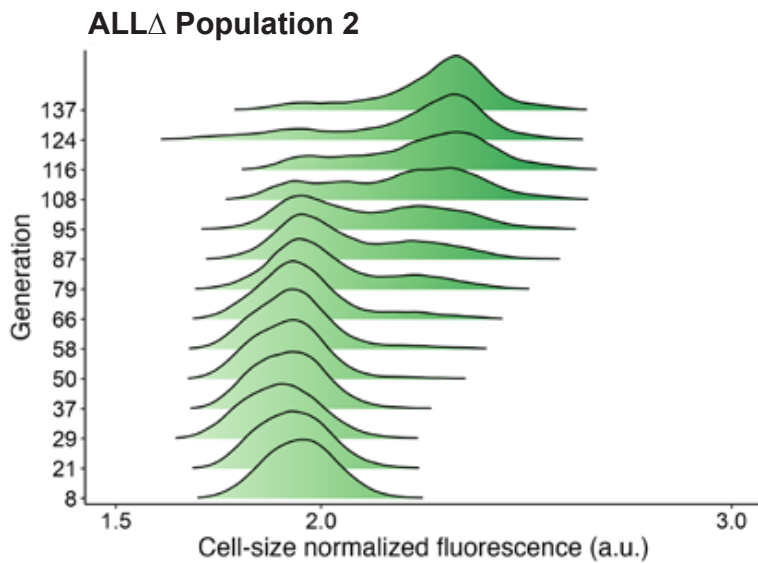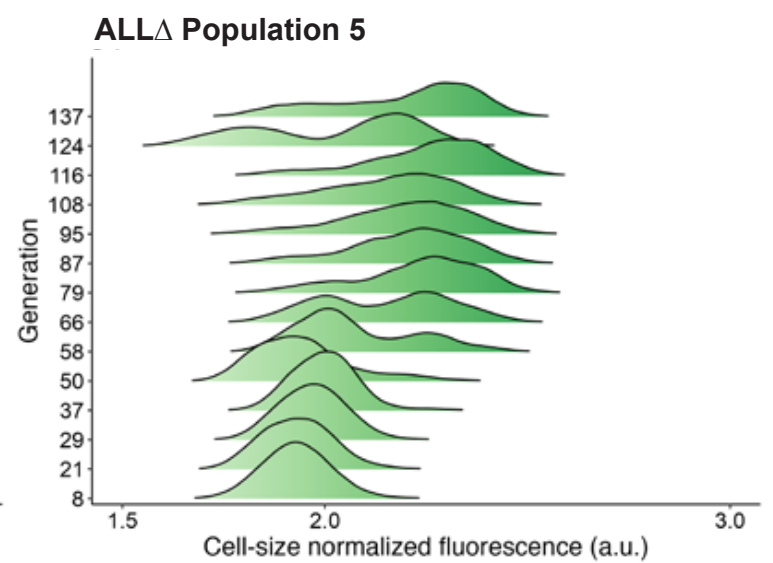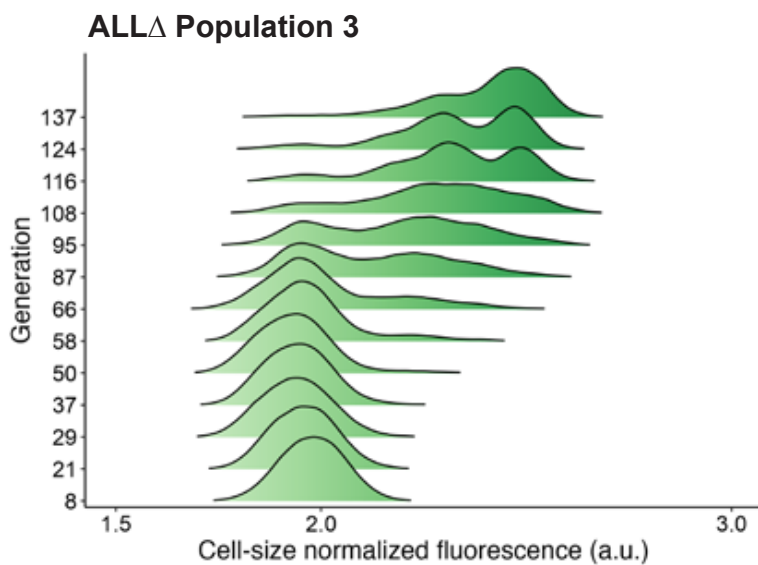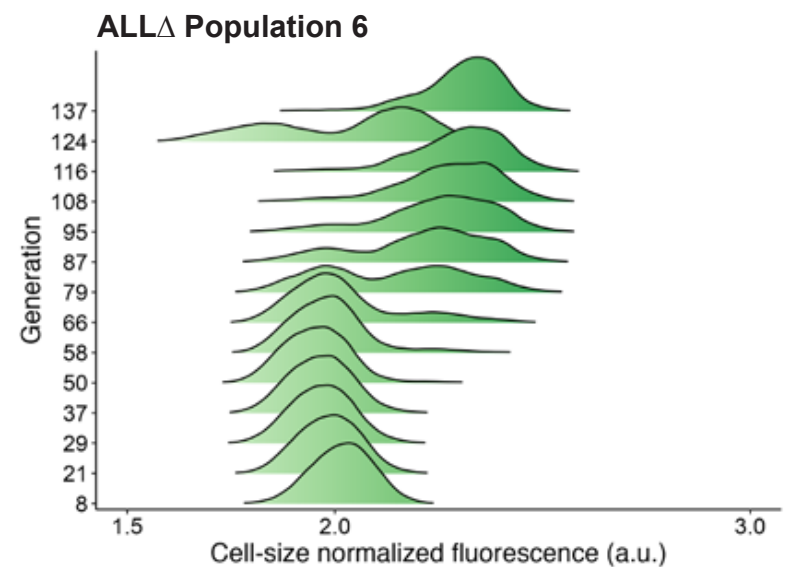

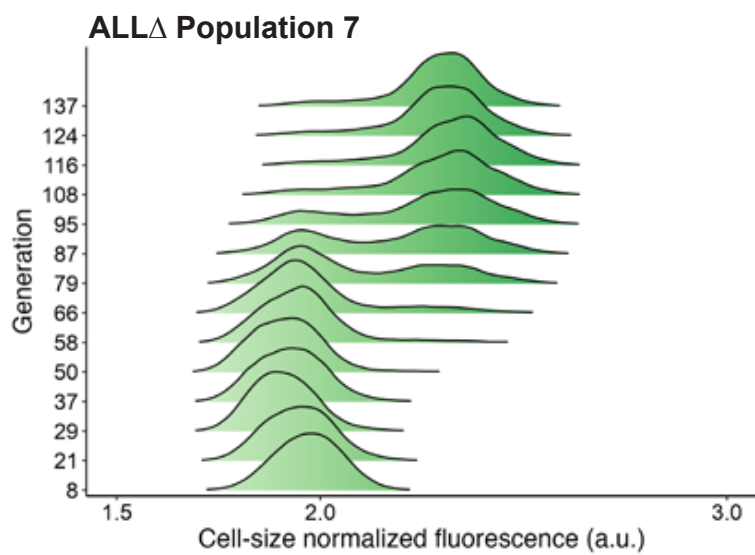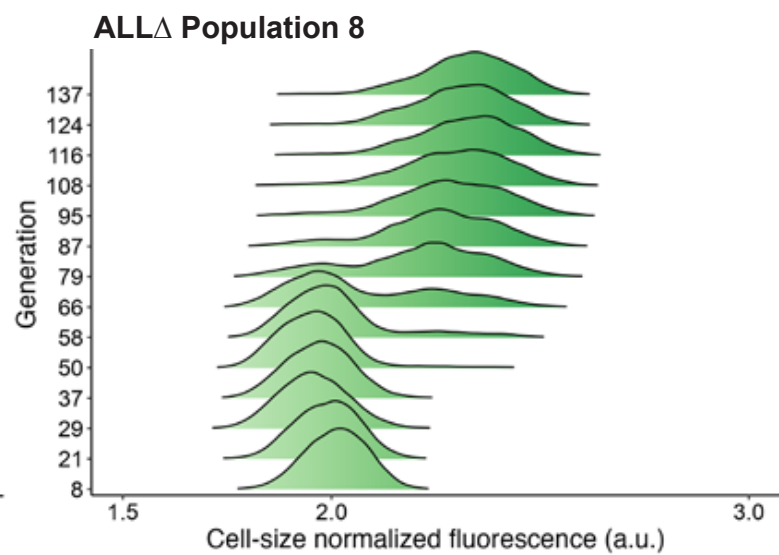

Supplement: Figure 2—source data 2. — Density plots of cell-size normalized GFP fluorescence in arbitrary units (a.u.) for every population and timepoint over the course of long-term experimental evolution in glutamine-limited chemostats. [file elife-98934-fig2-data2.pdf]
