# Supplementary material for: Template switching during DNA replication is a prevalent source of adaptive gene amplification
Source: eLife. 2025 Feb 3;13:RP98934. doi: 10.7554/eLife.98934 (PMC11790251; doi:10.7554/eLife.98934)
Supplement: Supplementary file 3. [file elife-98934-supp3.docx]

**Evolutionary model**

We developed a Wright-Fisher model that describes the evolutionary dynamics, similar to our previous study (Avecilla et al., 2022). This is a discrete-time, non-overlapping generations model with a constant population size. Our model follows the change in frequency of four genotypes (Fig 2A): $A$, the wildtype genotype; $B$, a cell with a non-CNV beneficial mutation; $C^{+}$, a genotype with two copies of GAP1 and two copies of the fluorescent reporter; and $C^{-}$, a genotype with two copies of GAP1 but only a single copy of the fluorescent reporter. CNV and non-CNV mutations are formed at a rate of $\delta_{C}$ and $\delta_{B}$ and have a fitness effect of $s_{C}$ and $s_{B}$, respectively.

Unlike $B$ and $C^{+}$, which may increase in frequency due to both mutation and selection, we assume that the $C^{-}$ genotype is not generated after generation 0 (as experimental results suggest that the reporter is working properly). Hence, the $C^{-}$ genotype only increases in frequency due to selection, with $s_{C}$ as its fitness effect. We assume $C^{-}$ has an initial frequency $X_{C^{-}}=\varphi$.
The model is fully described by the following derivations.

Initial genotype frequencies are:

$$X_{A}^{0}=1-\varphi X_{B}^{0}=0X_{C^{-}}^{0}=\varphi X_{C^{+}}^{0}=0$$

The change in genotype frequencies due to mutation is expressed by

$$X_{A}^{'}=\left( 1-\delta_{C}-\delta_{B} \right)X_{A}^{t}X_{B}^{'}=X_{B}^{t}+\delta_{B}X_{B}^{t}X_{C^{+}}^{'}=X_{C^{+}}^{t}+\delta_{C}X_{C^{+}}^{t}X_{C^{-}}^{'}=X_{C^{-}}^{t}$$

The change in genotype frequencies due to selection is expressed by $X_{i}^{*}=\frac{w_{i}X_{i}}{\underline{w}}$

where $w_{i}=1+s_{i}$ for $i=B,C^{-},C^{+}$ $(w_{A}=1, s_{C^{-}}=s_{C^{+}}=s_{C})$ is the relative fitness of genotype $i$ and $\underline{w}=\sum_{i\in\left\{ A,B,C^{+},C^{-} \right\}} w_{i}X_{i}$ is the population mean fitness.

The change in genotype frequencies due to random genetic drift is given by

$$n_{i}=Multinomial\left( N,\left( X_{A}^{*},X_{B}^{*},X_{C^{-}}^{*}, X_{C^{+}}^{*} \right) \right)$$

where $n_{i}$ is the number of cells with genotype *i*, and therefore the genotype frequencies in the next generation are$X_{i}^{t+1}=\frac{n_{i}}{N}$

where $N$ is the population size, set to be constant at $3.3\times{10}^{8}$, the effective population size in the chemostat (Avecilla et al., 2022). Overall, we have five model parameters: $s_{C},\delta_{C},s_{B},\delta_{B},\varphi$.

The empirical observations show the frequency of cells with multiple GAP1 reporters over time. This is $X_{C^{+}}^{t}$ in our model. Therefore, we can use the evolutionary model to simulate dynamics in the shape of the empirical observations, which we will then use for inference with NPE.

**Competition model.** Similarly, we can simulate a competition assay between two genotypes. The model steps are as follows:

Initial genotype frequencies are:

$$X_{A_{1}}^{0}=X_{A_{2}}^{0}=0.5$$

$$X_{B_{1}}^{0}=X_{B_{2}}^{0}=0X_{C_{1}}^{0}=X_{C_{2}}^{0}=0$$

The change in genotype frequencies due to mutation is expressed by

$$X_{A_{i}}^{'}=\left( 1-\delta_{C_{i}}-\delta_{B} \right)X_{A_{i}}^{t}X_{B_{i}}^{'}=X_{B_{i}}^{t}+\delta_{B}X_{B_{i}}^{t}X_{C_{i}}^{'}=X_{C_{i}}^{t}+\delta_{C_{i}}X_{C_{i}}^{t}$$

The change in genotype frequencies due to selection is expressed by $X_{G_{i}}^{*}=\frac{w_{i}X_{i}}{\underline{w}}$

where $w_{i}=1+s_{G_{i}}$ for $G=B,C$ and $i=1,2$ $(w_{A_{1}}=w_{A_{2}}=1)$ is the relative fitness of genotype $G_{i}$ and $\underline{w}=\sum_{G\in\left\{ A,B,C \right\}, i\in\left\{ 1,2 \right\}} w_{G_{i}}X_{G_{i}}$ is the population mean fitness.

Note that here we have different formation rates and selection coefficients for the two competing genotypes.

Genetic drift will be applied in the same manner as in the original model.

We’re interested in the competition’s outcome, i.e., $X_{i}^{T}=X_{A_{i}}^{T}+X_{B_{i}}^{T}+X_{C_{i}}^{T}$.

**Collective posterior distribution**

The above stochastic evolutionary model can be represented by a likelihood function $P\left( X|\theta\right)$ with *X* as a vector of genotype frequencies and $\theta$ as a vector of model parameters. In our case, *X* is actually a summary of genotype frequencies, as X(t) gives the frequency of cells with two or more GAP1 copies with reporters: $X\left( t \right)=X_{C^{+}}^{t}$.

Simulation-based Bayesian inference methods, such as ABC, produce a posterior distribution over model parameters by comparing model simulations to the empirical data when the likelihood is intractable. However, when a collection of observations is assumed to arrive from the same distribution, such as in repeated biological experiments, we are provided with more information that could be used for collective conclusions.
Dietrich (Dietrich, 2010) showed an axiomatic approach to finding a group belief in a Bayesian setting, that is, a collective belief based on all observations, relying on probability rules to form the probability of a hypothesis H given a collection of beliefs $E_{1},...,E_{n}$ - $P(H|E_{1},...,E_{n})$.

Given an evolutionary model, we could use a similar axiomatic approach to form $P(\theta|X_{1},...,X_{n})$ – a collective posterior distribution for model parameters θ, conditioned on all observations $X_{1},...,X_{n}$.

Let $X_{1},\ldots,X_{n}$be observations from the model such that $X_{i}\sim P\left( X|\theta\right)$. Assuming we have already estimated $P\left( X_{i} \right)$ using NPE, we wish to estimate $P\left( X_{1},\ldots,X_{n} \right)$.

Using Bayes’ theorem,

$$P\left( X_{1},\ldots X_{n} \right)=\frac{P(X_{1},\ldots,X_{n}|\theta)P(\theta)}{P\left( X_{1},\ldots X_{n} \right)} \left( 1 \right)$$

Assuming $X_{i},X_{j}$ are independent after conditioning on $\theta$, i.e., $P\left( \theta\right)=P\left( \theta\right)P\left( \theta\right)$, we have$P\left( X_{1},\ldots,X_{n} \right)=\frac{\Pi_{i}\left[ P\left( \theta\right) \right]}{P\left( X_{1},\ldots X_{n} \right)}P\left( \theta\right) \left( 2 \right)$

Using Bayes’ theorem again:

$$P\left( X_{1},\ldots,X_{n} \right)=\frac{\Pi_{i}\left[ P\left( X_{i} \right)P\left( X_{i} \right) \right]}{P\left( X_{1},\ldots X_{n} \right)}P\left( \theta\right)^{1-n} \left( 3 \right)$$

Next, recalling the law of total probability,

$$P\left( X_{1},\ldots,X_{n} \right)=\int P\left( \theta\right)P\left( \theta\right) d\theta$$

Using Bayes’ theorem once more,$P\left( X_{1},\ldots,X_{n} \right)=\int\frac{P\left( X_{1},\ldots,X_{n} \right)P\left( X_{1},\ldots,X_{n} \right)}{P\left( \theta\right)} P\left( \theta\right) d\theta$

As $P\left( X_{1},\ldots,X_{n} \right)$ is positive and independent of $\theta$, we can take it out of the integral and then cancel it from both sides$,$ giving

$$\int P\left( X_{1},\ldots,X_{n} \right) d\theta=1 \left( 4 \right)$$

Using Bayes’ theorem in eq. 4,

$$\int\frac{\Pi_{i}\left[ P\left( X_{i} \right)P\left( X_{i} \right) \right]}{P\left( X_{1},\ldots X_{n} \right)}P\left( \theta\right)^{1-n} d\theta=1$$

Using commutativity of multiplication,

$$\int\frac{\Pi_{i}\left[ P\left( X_{i} \right) \right]}{P\left( X_{1},\ldots X_{n} \right)}P\left( \theta\right)^{1-n} \Pi_{i}\left[ P\left( X_{i} \right) \right]d\theta=1 \left( 5 \right)$$

where $P(\theta)$, the prior distribution, is known to us (we chose it), and $P(\theta|X_{i})$, the indivdual posteriors, are estimated by NPE. Therefore, only the single and collective marginal likelihoods (i.e., ‘evidence’) $P(X_{i})$ and $P(X_{1},\ldots X_{n})$ are unknown, yet both are positive and independent of the parameters vector $\theta$. We can therefore define $C=\frac{\Pi_{j}P\left( X_{j} \right)}{P\left( X_{1},\ldots X_{n} \right)}$ and write

$$\int C\cdot P\left( \theta\right)^{1-n} \Pi_{i}\left[ P\left( X_{i} \right) \right] d\theta=1\Rightarrow C=\frac{1}{\int P\left( \theta\right)^{1-n}\Pi_{i}\left[ P\left( X_{I} \right) \right] d\theta} \left( 6 \right)$$

Thus, plugging C in eq. 3 (and changing the symbol for the integration variable) will result in the collective posterior,

$$P\left( X_{1},\ldots,X_{n} \right)=\frac{{P\left( \theta\right)^{1-n}\Pi}_{i}\left[ P\left( X_{i} \right) \right]}{\int P\left( \zeta\right)^{1-n}\Pi_{i}\left[ P\left( X_{i} \right) \right] d\zeta} (7)$$

However, as $P\left( X_{i} \right)$ could be infinitesimal, a single idiosyncratic observation could potentially reject a parameter value with a high probability for the other $n-1$ observations. We want the collective posterior to be robust to such unrepresentative or idiosyncratic observations. Therefore, we define $P_{\epsilon}\left( X_{i} \right)= max(\epsilon,P\left( X_{i} \right))$ so that individual posteriors give at least a value of $\epsilon$ to all parameter values. Thus, the collective posterior is

$$P_{\epsilon}\left( X_{1},\ldots,X_{n} \right)=\frac{{P\left( \theta\right)^{1-n}\Pi}_{i}\left[ P_{\epsilon}\left( X_{i} \right) \right]}{\int P\left( \zeta\right)^{1-n}\Pi_{i}\left[ P_{\epsilon}\left( X_{I} \right) \right] d\zeta} (8)$$

For a good choice of $\epsilon$, the mode of the collective posterior distribution should reflect a value with high posterior density for multiple observations, rather than a value that no observation completely rejects (which can occur when the $\epsilon$ correction is not applied).

**Genetic diversity**

We want to estimate the CNV diversity of the population, and specifically, the probability to draw two consecutive cells with identical CNVs from a chemostat, given the collective MAP estimations for the four different genetic backgrounds (${10}^{-6}\leq\delta_{C}\leq{10}^{-4}, {0.1\leq s}_{C}\leq0.2$).

We assume that each newly formed CNV is different in its specific genotype from all CNV formed before it. Thus, *Q(t)* is the number of new CNV cells at generation *t*, that is, the number of CNV cells in generation *t* that have a non-CNV parent cell in generation *t-1*. *P(t)* is the number of cells in the last generation *T* that are descendants of a new CNV cell from generation *t* (i.e., that had a non-CNV parent in generation *t-1*). To compute Q(t) and P(t) given the results of an evolutionary simulation, $X_{A}^{t}$, $X_{B}^{t}$, $X_{C^{+}}^{t}$, and $X_{C^{-}}^{t}$:

At each generation $t=1,\ldots,T$:

1. Formation of new CNVs: $Q(t)=P(t)=\delta_{C}X_{A}^{t}$
2. Computation of mean fitness at generation *t*: $\underline{w}\left( t \right)={\sum_{i\in\left\{ A,B,C^{+},C^{-} \right\}} w_{i}X_{i}}^{t}$
3. For $k=1,\ldots,t$:
   - Application of natural selection: $P(k)=\frac{1+s_{c}}{\underline{w}(t)}P(k)$

Next, we define p(t) as the probability that two randomly chosen cells in the last generation are both descendants of CNV cells that were formed in generation *t* (i.e., have a non-CNV parent in generation *t-1*). We also define *q(t)* as the probability that two randomly chosen cells in the last generation that are both descendants of CNV cells formed in generation *t* are actually descended from the same cell. To compute p(t) and q(t):

For each generation $t=1,\ldots,T$:

1. $p(t)=\frac{P(t)}{\Sigma_{t}P(t)}\cdot\frac{P(t)-1}{\Sigma_{t}P(t)}\approx\left( \frac{P(t)}{\Sigma_{t}P(t)} \right)^{2}$
2. $q(t)\approx\frac{1}{Q(t)}$

Then, we compute S, the probability to draw two cells from the last generation that have the same CNV:

$$S=\sum_{t=1}^{T} p(t)\cdot q(t)\approx\frac{\sum_{t=1}^{T} \frac{{P(t)}^{2}}{Q(t)}}{\left( \sum_{t=1}^{T} P(t) \right)^{2}}$$

Note that the denominator has the square of the total number of CNVs generated over the entire simulation or experiment.

We compute the CNV diversity as follows:

In generation $t$, $Q(t)$ lineages of size $\frac{P\left( t \right)}{Q\left( t \right)}$have formed, resulting in a $P(t)$ CNV cells in generation *T*. The Shannon diversity index, given by ${-\Sigma}_{i=1}^{s}{[f}_{i}\cdot loglog f_{i} ]$ where *f_i_* is the frequency of genotype *I*, (Jost 2006), is therefore

${-\Sigma}_{t=0}^{T}\left[ Q(t)\cdot\frac{\frac{P\left( t \right)}{Q\left( t \right)}}{\sum_{t=1}^{T} P\left( t \right)}\cdot lnln \left( \frac{\frac{P\left( t \right)}{Q\left( t \right)}}{\sum_{t=1}^{T} P\left( t \right)} \right) \right]={-\Sigma}_{t=0}^{T}\left[ \frac{P\left( t \right)}{\sum_{t=1}^{T} P\left( t \right)}\cdot loglog \left( \frac{\frac{P\left( t \right)}{Q\left( t \right)}}{\sum_{t=1}^{T} P\left( t \right)} \right) \right]$
